# A Theoretical Study of the Benzoylformate Decarboxylase Reaction Mechanism

**DOI:** 10.3389/fchem.2018.00205

**Published:** 2018-06-26

**Authors:** Ferran Planas, Xiang Sheng, Michael J. McLeish, Fahmi Himo

**Affiliations:** ^1^Arrhenius Laboratory, Department of Organic Chemistry, Stockholm University, Stockholm, Sweden; ^2^Department of Chemistry and Chemical Biology, Indiana University-Purdue University Indianapolis, Indianapolis, IN, United States

**Keywords:** enzyme mechanism, computational chemistry, active site model, benzoylformate decarboxylase, transition state, catalytic mechanism, potential energy profile, thiamin diphosphate

## Abstract

Density functional theory calculations are used to investigate the detailed reaction mechanism of benzoylformate decarboxylase, a thiamin diphosphate (ThDP)-dependent enzyme that catalyzes the nonoxidative decarboxylation of benzoylformate yielding benzaldehyde and carbon dioxide. A large model of the active site is constructed on the basis of the X-ray structure, and it is used to characterize the involved intermediates and transition states and evaluate their energies. There is generally good agreement between the calculations and available experimental data. The roles of the various active site residues are discussed and the results are compared to mutagenesis experiments. Importantly, the calculations identify off-cycle intermediate species of the ThDP cofactor that can have implications on the kinetics of the reaction.

## Introduction

Benzoylformate decarboxylase (BFDC) is a thiamin diphosphate (ThDP)-dependent enzyme that catalyzes the nonoxidative decarboxylation of benzoylformate generating benzaldehyde and carbon dioxide (Hegeman, 1970). Originally isolated from *Pseudomonas putida*, BFDC was found to be the penultimate enzyme in the mandelate pathway, a secondary metabolic pathway that allows various pseudomonads to grow using *R*-mandelate as their sole source of carbon (Hegeman, 1966, 1970).

Decarboxylation reactions are often catalyzed by ThDP-dependent enzymes, and, in fact, the decarboxylases make up the largest group of this family of enzymes (Andrews and McLeish, 2012). Pyruvate decarboxylase (PDC), the archetypal member of this group, catalyzes the nonoxidative decarboxylation of pyruvate to yield acetaldehyde and carbon dioxide, a reaction critical to the fermentation pathway of several yeast and bacteria (Nichols et al., 2003). X-ray structures of PDCs from a variety of species show that, in addition to ThDP and the almost invariant glutamate, the active site contains two ionizable acidic residues as well as two contiguous histidine residues that are located on an ordered loop (Dyda et al., 1993; Dobritzsch et al., 1998; Kutter et al., 2006; Rother neé Gocke et al., 2011). The latter has been termed the HH-motif, and mutagenesis and kinetic studies have revealed that the histidines and the acidic residues all play significant roles in catalysis (Candy and Duggleby, 1998; Liu et al., 2001; Sergienko and Jordan, 2001). Subsequently, X-ray structures of several other ThDP-dependent decarboxylases show that most possess the HH-motif and the two acidic residues, with differences in substrate specificity being explained by the variety of residues lining the substrate-binding pocket (Schütz et al., 2003; Berthold et al., 2007; Versées et al., 2007).

**Figure 1:**
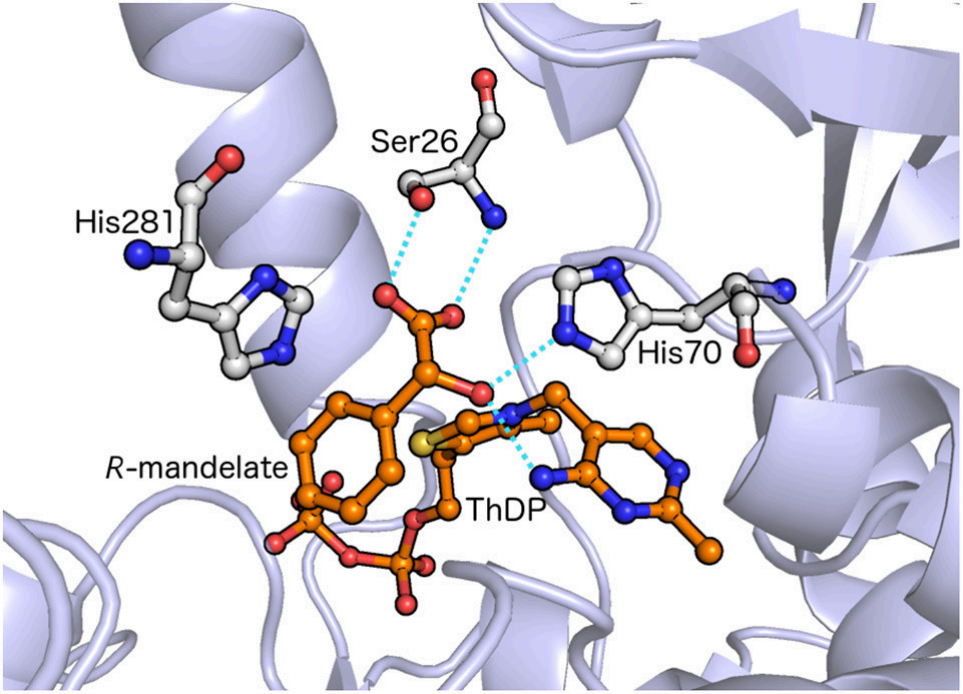
X-ray structure of the BFDC active site with substrate analog *R*-mandelate bound. Potential catalytic residues are indicated. Dashed lines indicate possible hydrogen bonds. Coordinates taken from PDB 1MCZ.

Given the apparently common active site architecture, it was somewhat of a surprise when the first X-ray structure of BFDC showed that the active site contained a single serine residue rather than the two acidic residues found in the PDCs. Further, although there are two histidine residues that are in spatially similar positions to those of the HH-motif decarboxylases, His70 and His281 are located on separate monomers that together comprise the active site (Hasson et al., 1998). Subsequently, the X-ray structure of BFDC in complex with the substrate analog, *R*-mandelate, showed that Ser26, His70 and the 4′-imino group of ThDP all made hydrogen bonds to *R*-mandelate (Figure 1). Further, His281, the other potential acid-base catalyst, was located <3.5 Å from the inhibitor (Polovnikova et al., 2003). Taken together, the indications were that Ser26, His70, and His281 would play important roles in the mechanism of BFDC.

**Scheme 1:**
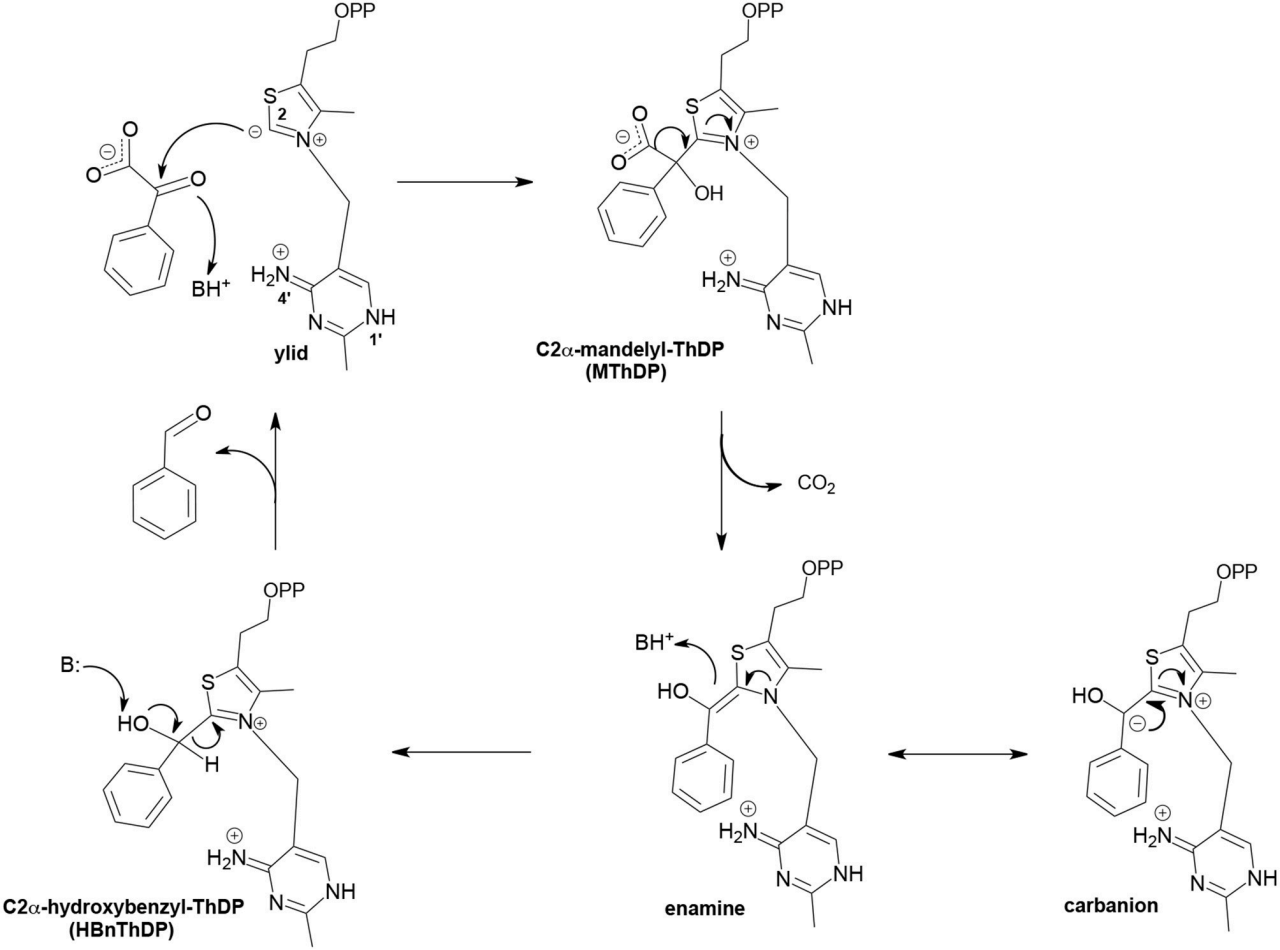
General mechanism for the ThDP-assisted decarboxylation of benzoylformate.

Starting with the formation of a C2-carbanion or ylid intermediate (Breslow, 1958) brought about by the deprotonation of the C2 carbon by the 4′-imino group, a typical ThDP-assisted decarboxylation reaction proceeds through a series of ThDP-bound complexes as shown in Scheme 1. For the reaction catalyzed by BFDC, the ThDP complexes include the pre-decarboxylation tetrahedral intermediate (C2α-mandelyl-ThDP, MThDP), the post-decarboxylation carbanion-enamine (Breslow) intermediate, and a second tetrahedral intermediate (C2α-hydroxybenzyl-ThDP, HBnThDP).

Most of the early studies on the BFDC reaction mechanism were focused on identifying the contributions of the ionizable active site residues to each of the individual steps in the mechanism (Sergienko et al., 2000; Polovnikova et al., 2003). Overall, a combination of X-ray crystallography, site-directed mutagenesis, steady-state, and stopped-flow kinetics (the last employing the slow substrate, p-nitrobenzoylformate) was employed in the studies. A comparison of wild-type BFDC kinetic data with those of the S26A, H70A, and H281A variants was used to allocate mechanistic roles to Ser26, His70, and His281. The results clearly indicated that His70 played a role in decarboxylation and product release by assisting in the protonation of the MThDP intermediate, and the deprotonation of the HBnThDP intermediate, respectively (Scheme 1). His281 was implicated in the protonation of the carbanion/enamine while Ser26 was involved in substrate binding and, in a less defined manner, the other steps in the mechanism (Sergienko et al., 2000; Polovnikova et al., 2003).

More recently, two studies have brought these assignments into question. The first study was focused on the development of a saturation mutagenesis methodology to explore BFDC substrate specificity (Yep et al., 2008). Unexpectedly, it was found that His281 could be replaced by phenylalanine with only a 5-fold decrease in *k*_cat_ value. Subsequent replacement of His281 by Trp and Glu also resulted in <20-fold decrease, yet such substitutions were incompatible with the proposed role of His281 as a proton donor (Yep et al., 2008). Further investigation showed that replacement of His70 with Thr only provided an 8-fold reduction in *k*_cat_ value, while even Leu could be utilized with only a *ca*. 20-fold reduction. None of these results were consistent with the His70 acting as an acid-base catalyst. Finally, Ser26 could be replaced by Leu with only *ca*. 2.5-fold decrease in *k*_cat_ and a marginal increase in the value of *K*_m_. In fact, Ser26 could even be replaced by Met with little effect on *K*_m_. This was perhaps the most surprising as the X-ray structure clearly showed that *R*-mandelate had hydrogen bonding interactions with the side chain of Ser26 (Figure 1). Indeed, for all three residues the results with alanine variants (Sergienko et al., 2000; Polovnikova et al., 2003) seemed to be outliers leading the conclusion that, even when structural information was available, assigning mechanistic roles solely on the basis of alanine variants could be hazardous (Yep et al., 2008).

The second study employed a combined chemical quench/^1^H NMR approach to analyze the steady-state distribution of ThDP, MThDP, and HBnThDP in the wtBFDC reaction and the reaction of BFDC with p-nitrobenzoylformate (Bruning et al., 2009). The study concluded that decarboxylation is the fastest step in the BFDC, and that the formation of MThDP (primarily) and release of benzaldehyde from HBnThDP are rate-limiting for catalysis. Further, the results indicated that the charge-transfer complex, attributed to the corresponding MThDP in the p-nitrobenzoylformate reaction (Sergienko et al., 2000), is more likely to belong to the substrate Michaelis complex. A second charge-transfer complex was assigned to the enamine form of the long-lived HBnThDP intermediate (Bruning et al., 2009). Since the initial allocation of the roles of Ser26, His70, and His281 in the BFDC mechanism was predicated on (seemingly) incorrect identification of charge-transfer complexes (Polovnikova et al., 2003), these roles should now be re-evaluated.

In the present work, quantum chemical methodology has been used to investigate the detailed reaction mechanism of BFDC. A large model of the active site was designed on the basis of the crystal structure, and all intermediates and transition states were located and characterized. The adopted methodology, called the cluster approach, has been very successful in elucidating reaction mechanisms of a wide variety of enzymes (Siegbahn and Himo, 2009, 2011; Siegbahn and Blomberg, 2010; Blomberg et al., 2014; Himo, 2017). A number of previous theoretical studies using a variety of computational techniques have considered aspects of mechanism of a number of ThDP-dependent enzymes (Friedemann et al., 2004; Lie et al., 2005; Wang et al., 2005; Jaña et al., 2010; Topal et al., 2010; Xiong et al., 2010; Paramasivam et al., 2011; Alvarado et al., 2012; Hou et al., 2012; Jaña and Delgado, 2013; Sheng and Liu, 2013; Sheng et al., 2013, 2014; Sánchez et al., 2014; Zhang et al., 2014; Zhu and Liu, 2014; Lizana et al., 2015; Nauton et al., 2016; White et al., 2016). Relevant results from these studies have been incorporated into the discussion of the BFDC mechanism below.

## Computational details

All calculations were performed using the Gaussian 09 program (Frisch et al., 2016) and B3LYP functional (Lee et al., 1988; Becke, 1993). Geometry optimizations and zero-point energy corrections were calculated with 6-31G (d, p) basis set, and the optimizations included the dispersion correction according to the D3 (BJ) method (Grimme et al., 2010, 2011). At the same level of theory, single-point calculations with SMD solvation model (Marenich et al., 2009) and dielectric constant of ε = 4 were carried out to mimic the effect of the rest of protein body. Further single-point energies with a larger basis set 6-311+G (2d, 2p) were performed to gain higher accuracy in energies. The final reported values are thus the large basis set energies corrected for solvation and zero-point energy. The constraints that are imposed in the model introduce small imaginary frequencies in the calculations, all of them under 50*i* cm^−1^. Although it has been demonstrated that entropy only has a small effect on the energies of the chemical step of enzyme reactions (Senn et al., 2005, 2009; Hu and Zhang, 2006; Lonsdale et al., 2012; Kazemi et al., 2016; Mulholland, 2016), its contribution must be considered in the case of a release of a gas molecule, which is relevant for the CO_2_ release considered in the current study. In line with previous studies (Blomberg and Siegbahn, 2012; Lind and Himo, 2014; Sheng et al., 2015, 2017), the entropy gain can be estimated as the translational entropy for the gas molecule (calculated to 11.3 kcal/mol), which is added at all steps after the transition state of CO_2_ formation. Finally, the energies of the transition states corresponding to **Int2** → **Int3a**, **Int4b** → **Int5**, and **Int6** → **Int7** steps were computed to be slightly lower than one or both of the connecting intermediates. The fact that corrections (large basis set, ZPE, and solvation) are added as single-points to the geometry optimized in the gas phase can lead to these transition state artifacts, which have been observed in previous studies (Cassimjee et al., 2015). In these cases, the energy of one of the two connected intermediates is considered to be the effective barrier. Calculated energies and energy corrections, along with the Cartesian coordinates of the stationary points are given in Supplementary Material.

## Active site model

**Figure 2:**
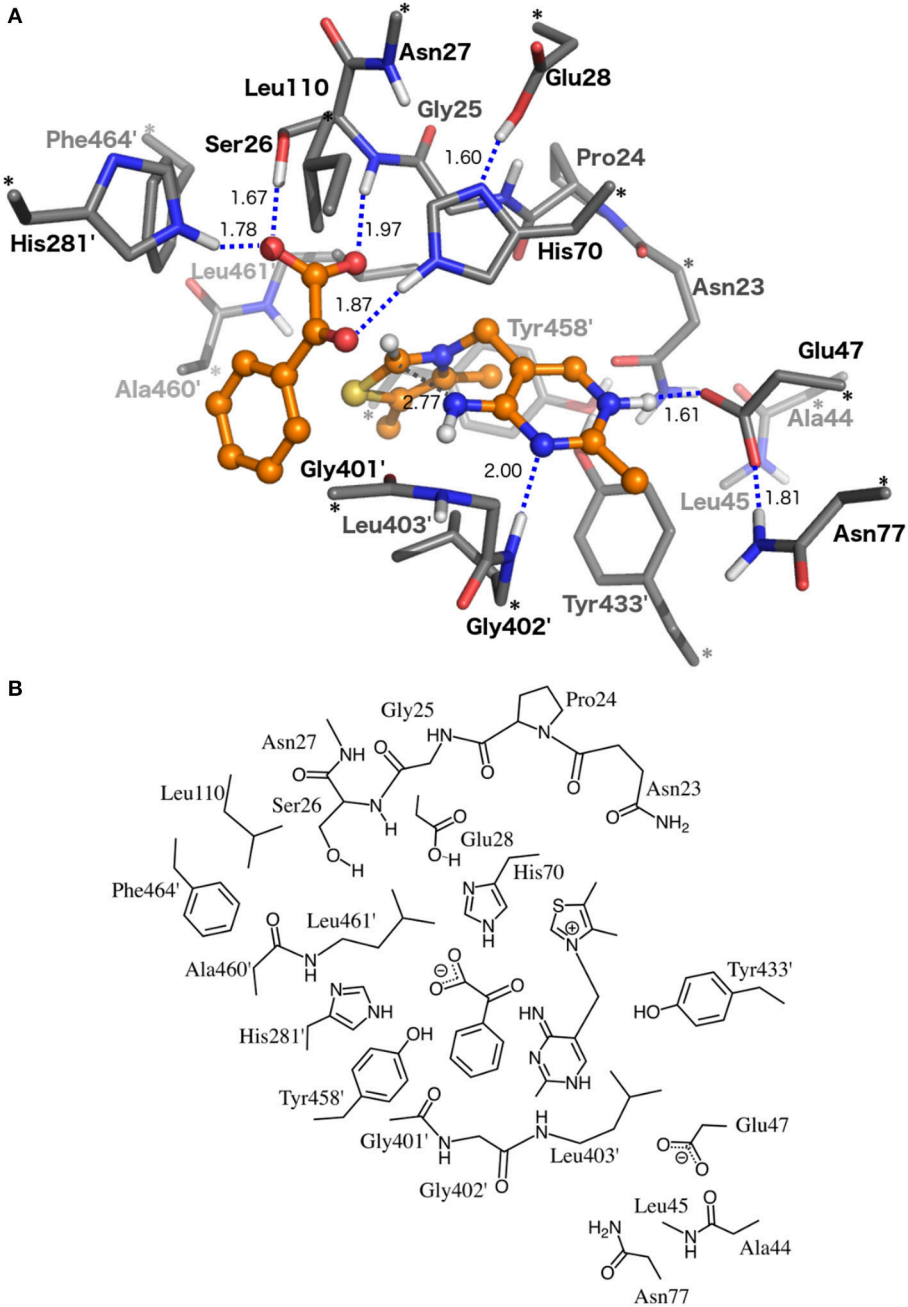
**(A)** Optimized structure of the active site model for the BFDC enzyme-substrate (ES) complex. **(B)** 2D representation of the same structure. Asterisks (*) mark the atoms at which the model is truncated. Primes indicate residues from the second active site monomer. Note that most of the hydrogen atoms in the upper figure are left out for better clarity.

The reaction mechanism was studied using an active site model designed from the crystal structure of benzoylformate decarboxylase in complex with the inhibitor, *R*-mandelate (PDB id 1MCZ; Hasson et al., 1998). The model consists of the residues directly interacting with the substrate and those lining the cofactor-binding pocket. Ser26, His70, His281, Leu110, and Phe464 interact directly with the substrate and, as discussed above, the first three have been shown to be catalytically important (Hasson et al., 1998; Sergienko et al., 2000; Polovnikova et al., 2003). Residues surrounding the cofactor are Asn23, Pro24, Glu28, Gln47, Gly401, Gly402, Leu403, Tyr433, Tyr458, Ala460, and Leu461. In addition, Gly25, Asn27, Glu28, Ala44, Leu45, and Asn77 were also included. Gly25 and Asn27 were added to give more flexibility to residues directly interacting with the substrate, while the others interact with one or more of the residues listed above. The benzoylformate (BF) molecule was built directly into the cavity using the position of the bound *R*-mandelate as a reference. The various residues and the cofactor are truncated as shown in Figure 2.

The protonation states of the various titratable groups were chosen as follows. His281 was modeled in the neutral form with the proton at the ε position. This choice is made because this histidine forms a hydrogen bond to the carboxylate group of the inhibitor *R*-mandelate in the crystal structure and is expected to form the same hydrogen bond to the substrate. His70 was also modeled with the proton at the ε position as it forms a hydrogen bond to the carbonyl oxygen of the substrate. The Glu28 residue was modeled in the protonated form, as it is found to form a hydrogen bond to the δ-nitrogen of His70 in the crystal structure. Finally, Glu47 was modeled in the ionized form as it forms a hydrogen bond to the protonated N1'-nitrogen of the cofactor.

To mimic the constraints that the rest of the protein body imposes on the active site, and to avoid excessive motion of the residues, the atoms where the truncation is made are kept fixed at their crystallographic position in all geometry optimizations. In Figure 2, these atoms are indicated by asterisks. After addition of the hydrogen atoms, the model consists of 307 atoms and has a net charge of −1.

## Results and discussion

### Catalytic cycle

Although the native form of ThDP is the **AP** state (N4′ nitrogen in the NH_2_ form), the **IP** state (N4′ nitrogen in a NH form) is the productive one after the substrate is bound (Kern et al., 1997; Hübner et al., 1998; Nemeria et al., 2007; Patel et al., 2014). This will be the starting point of the computational study of the catalytic cycle in the present study.

In the optimized structure of the enzyme-substrate (**ES**) complex shown in Figure 2, no significant movements of the residues are observed compared to the X-ray structure. The N4′-C2 distance, commonly used in the literature as a measure of the V-shape of the cofactor, is somewhat shorter in the optimized ES compared to the X-ray structure (2.77 vs. 3.27 Å) due to a slight bending of ThDP. The hydroxyl moiety and the backbone NH of Ser26 form hydrogen bonds with the carboxylate of the substrate. His70 and His281, both protonated at Nε position, form hydrogen bonds with the carbonyl group of the benzylic carbon and the carboxylate, respectively.

**Figure 3:**
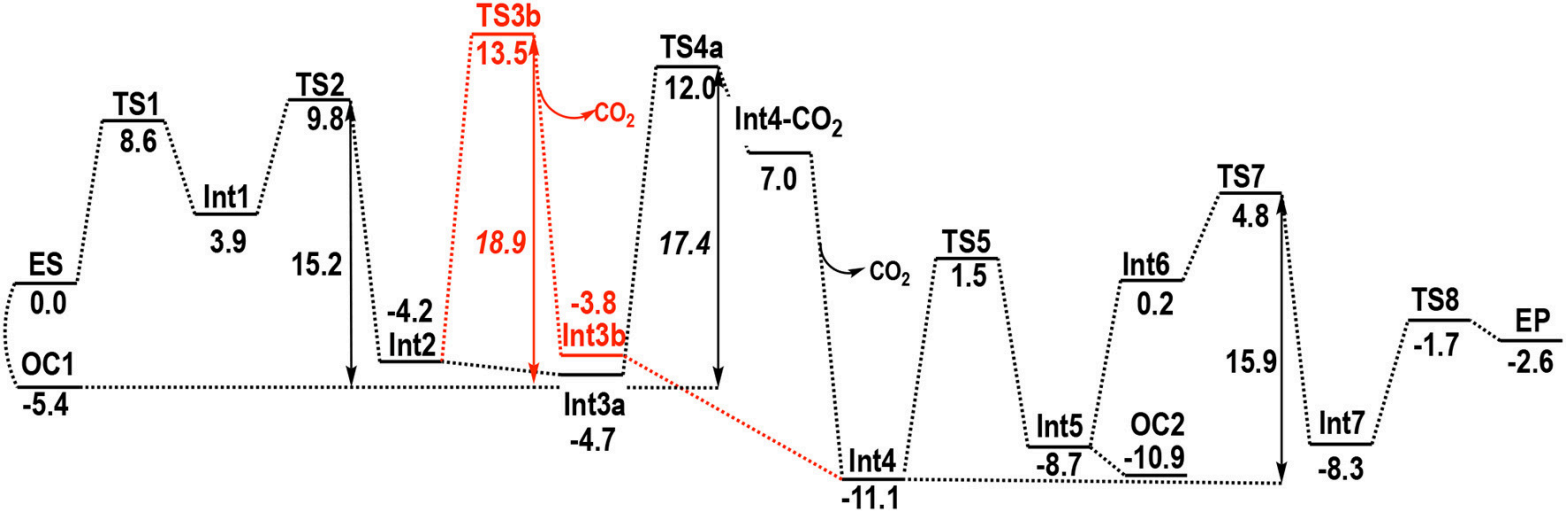
Calculated energy profile for the BFDC-catalyzed decarboxylation of benzoylformate. Energies in kcal/mol. Red color indicates an alternative pathway for these steps as discussed in the text.

**Scheme 2:**
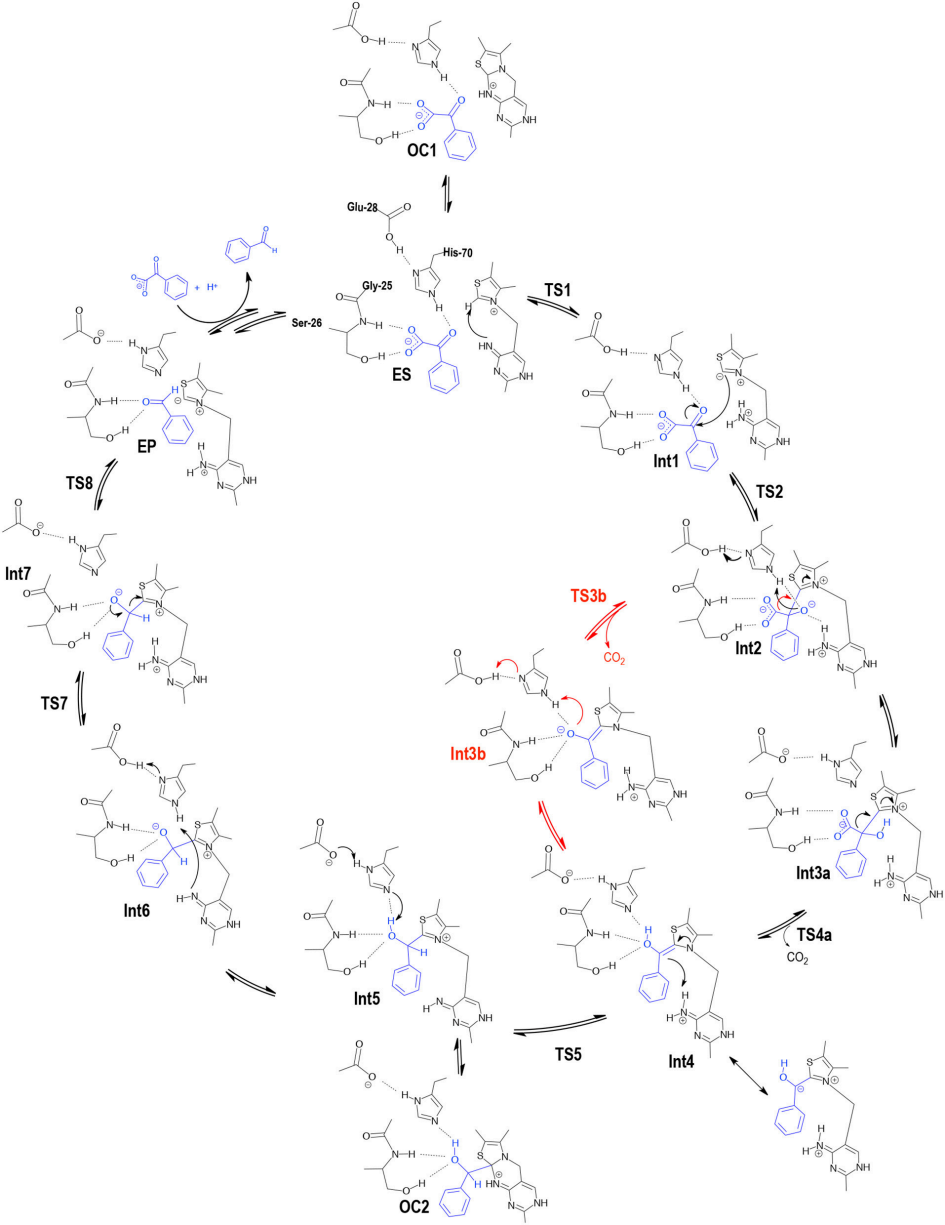
Detailed mechanism for BFDC obtained on the basis of the present calculations. Red color indicates an alternative pathway for these steps as discussed in the text.

**Figure 4:**
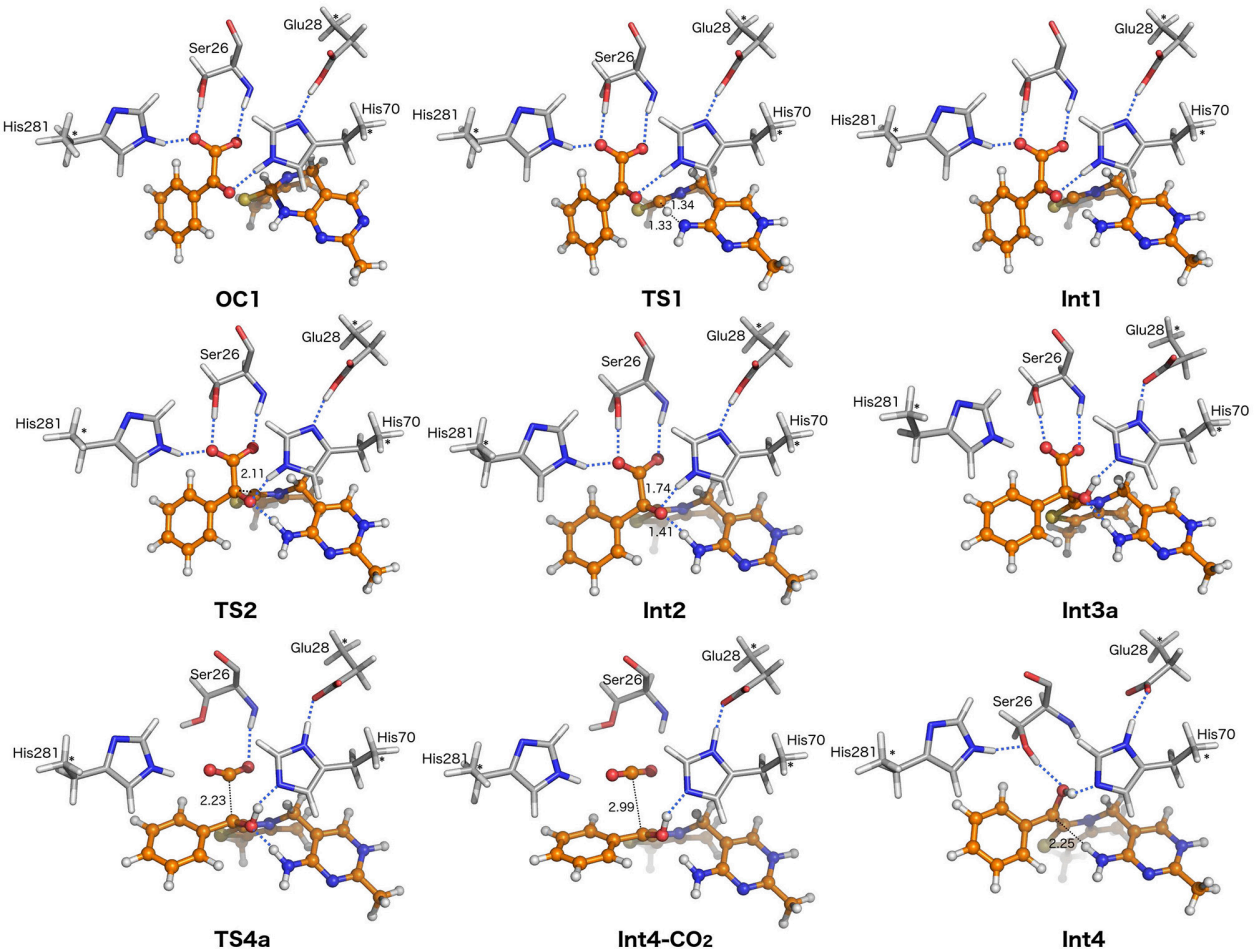
Optimized structures of stationary points along the reaction pathway. For clarity, only a small portion of the model is shown. Distances are given in angstroms.

**Figure 5:**
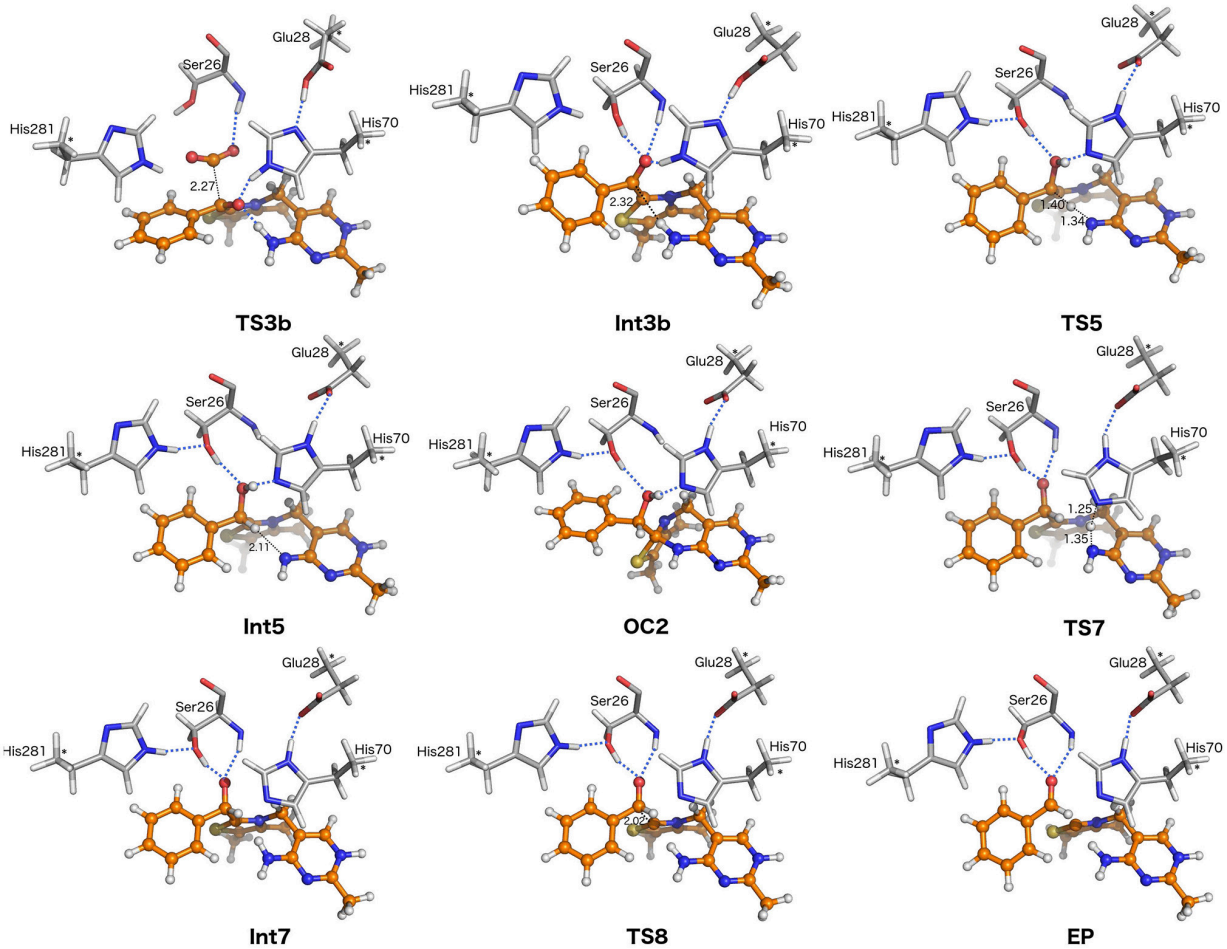
Optimized structures of stationary points along the reaction pathway. For clarity, only a small portion of the model is shown. Distances are given in angstroms.

The energy of the **ES** complex is set to 0.0 kcal/mol and the energies of all transition states and intermediates are referenced to this Michaelis complex in the calculated energy profile shown in Figure 3. The reaction mechanism arising from this study is summarized in Scheme 2 and the optimized structures are presented in Figures 4, 5.

For ThDP to perform nucleophilic/covalent catalysis, it needs to be converted to its active form. This occurs in the first step of the mechanism wherein an intramolecular proton transfer from C2 to N4′ results in the formation of an active ylid (Kern et al., 1997; Hübner et al., 1998). The barrier for this step (**TS1**) is calculated to be 8.6 kcal/mol and the resulting ylid intermediate, **Int1**, is +3.9 kcal/mol in energy compared to **ES**. The N4′-C2 distance is shortened from 2.77 Å in **ES** to 2.57 Å in **TS1** and, again, none of the surrounding residues move significantly.

The activation of the cofactor through an intramolecular proton transfer is consistent with previous computational studies, on PDC (Wang et al., 2005) and acetohydroxyacid synthase (AHAS; Xiong et al., 2010), as well as experimental work (Kern et al., 1997; Hübner et al., 1998; Brandt et al., 2009). It should be noted that theoretical studies on other ThDP-dependent enzymes have suggested that the ylid formation could be achieved by a proton transfer to histidine via a water shuttle (Lie et al., 2005; Nauton et al., 2016; White et al., 2016). While, in unliganded BFDC, there is a water molecule in the active site, that water molecule has been replaced by *R*-mandelate and, presumably, would be also be displaced by bound substrate. Thus, after substrate binding, the water shuttle mechanism is not viable here.

One interesting and potentially important discovery of the current calculations is that, starting from the **ES** complex, an intramolecular cyclization of the cofactor is also possible. The product of this reaction, denoted **OC1** in Figure 3, Scheme 2, has a covalent bond between N4′ and C2 and is 5.4 kcal/mol more stable than **ES**. However, as the mechanism cannot continue from this intermediate, **OC1** is non-productive and must be regarded as an off-cycle species. Nonetheless, its existence will have implications on the energetics of the following steps. For example, it will raise the barrier for the ylid formation from 8.6 to 14.0 kcal/mol.

In the second step, nucleophilic addition of the ylid to benzoylformate provides intermediate **Int2**. In **Int1**, the benzylic carbon of BF is ca 3 Å from the carbanion of the ylid, but at the transition state of the addition (**TS2**) the C-C distance has shortened to 2.11 Å. Concomitantly, the increasing negative charge on the benzylic oxygen is stabilized by an oxyanion hole formed by His70 and the 4′-NH_2_ group from ThDP. These hydrogen bonds remain in **Int2**, with NH^…^O distances of 1.74 Å for His70 and 1.41 Å for ThDP. Although the movement of BF toward ThDP does not break the hydrogen bonds with Ser26, the H-bond with His281 elongates to 2.03 Å. The resulting tetrahedral intermediate, **Int2**, is 4.2 kcal/mol more stable than **ES**. The addition has an energy barrier of 5.9 kcal/mol relative to **Int1**, providing an overall barrier of 15.2 kcal/mol relative to **OC1**.

The two steps following **Int2** comprise a proton transfer between the substrate and His70, and a C-C bond cleavage. Interestingly, two distinct paths could be found for these steps, albeit with very similar energy barriers. The first path (Figure 3, Scheme 2) starts with a concerted proton transfer from Glu28 to His70, and from His70 to the alkoxide. In this proton transfer, corresponding to the **Int2** → **Int3a** transition, His70 acts as a relay and the net proton transfer is thus from Glu28 to the C2α-alkoxide. This first step is calculated to be barrier-less^1^, and results in the formation of **Int3a**, the benzylic alcohol intermediate commonly known as mandelyl-ThDP (MThDP; Scheme 1). The calculated energy of **Int3a** is only 0.5 kcal/mol lower than **Int2**. After formation of **Int3a**, C-C bond cleavage occurs via **TS4a** giving rise to the carbanion/enamine (Breslow) intermediate (**Int4**) and CO_2_. During that process, the breaking C-C bond is elongated from 1.58 Å in **Int3a** to 2.23 Å at **TS4a**. The calculated barrier for the decarboxylation step is 16.7 kcal/mol relative to **Int3a**, i.e., overall 17.4 kcal/mol relative to **OC1**. It must be emphasized that the hydrogen bond between the hydroxyl moiety of Ser26 and the carboxylate group of the substrate must be broken in **TS4a** (Figure 4). All attempts to optimize the geometry of the TS with the hydrogen bond intact led back to the previous intermediate, **Int3a**.

The intermediate resulting from the C-C bond cleavage, still retaining CO_2_ in the active site, is called **Int4-CO**_2_ (Figure 4). This intermediate is 5.0 kcal/mol lower than the transition state **TS4a**, i.e., 11.7 kcal/mol higher in energy than **Int3a**. Protonation of the carbon of the enamine in **Int4-CO**_2_ has a calculated barrier of 37.3 kcal/mol relative to **Int3a**. Therefore, energetically, no further reaction is possible with gaseous CO_2_ in the active site pocket so the CO_2_ must be released. This is likely due to steric reasons, as the presence of the CO_2_ molecule prevents a structural change required for the following proton transfer to take place.

The CO_2_ release results in the enamine/carbanion (Breslow) intermediate **Int4**, which, after the inclusion of an entropy correction discussed in the computational details, is 18.1 kcal/mol lower in energy than **Int4-CO**_2_. Apart from the entropy gain, CO_2_ release also creates more space in the active site cavity, enabling the enamine to rotate and change its conformation (compare **Int4-CO**_2_ and **Int4** in Figure 4). It is interesting to observe that this rotation breaks a hydrogen bond with the imine of ThDP. In doing so the hydrogen bond between Ser26 and the substrate that was lost at the C-C bond cleavage transition state, is re-formed in **Int4** and is maintained in all the following steps. At the same time, the distance between the benzylic carbon and N4′ reduces to 2.25 Å. Furthermore, a hydrogen bond is formed between His281 and Ser26 at **Int4** and is also maintained in the following steps.

In the alternative path toward **Int4** formation (red path in Figure 3, Scheme 2), decarboxylation occurs directly from **Int2**, with a breaking C-C bond distance of 2.27 Å in the transition state **TS3b** (Figure 5). For this step, the calculated barrier is 17.7 kcal/mol relative to **Int2** (18.9 kcal/mol relative to **OC1**). As in the alternative path, in order to break the C-C bond, it is essential that the hydrogen bond between the Ser26 hydroxyl and the carboxylate moiety is also broken. Moreover, at **TS3b** the carboxylate moiety rotates and breaks its interaction with His281 (Figure 5). Once CO_2_ is released and the enamine has rotated, a proton is transferred from Glu28 to the C2α-alkoxide, with His70 acting as a relay, resulting in **Int4**. This proton transfer corresponds to the **Int3b** → **Int4** transition and is exothermic by 7.3 kcal/mol and also found to be barrier-less^1^. Overall the two pathways from **Int2** to **Int4** have very similar energy barriers, differing by only 1.5 kcal/mol. It is therefore not possible to conclusively identify the operative pathway based on the current calculations.

In the following step, the calculations show that protonation of the enamine/carbanion (**Int4**) occurs via an intramolecular proton transfer from the protonated imine of the cofactor to the benzylic carbon (i.e., C2α; **TS5**). This results in the alcohol form of a second tetrahedral intermediate, **Int5**, commonly referred to as hydroxybenzylThDP (HBnThDP). This step has a calculated barrier of 12.6 kcal/mol, relative to **Int4**, and the resulting intermediate (**Int5**) is calculated to be +2.4 kcal/mol compared to **Int4**. Throughout the process, the enamine (**Int4**), the transition state (**TS5**), and the tetrahedral intermediate (**Int5**) maintain the same hydrogen-bonding network with Ser26 and His70, as shown in Figure 5.

Intriguingly, from **Int5**, the formation of a second tricyclic species is also possible, again as a consequence of the nucleophilic attack by N4′ on C2 as described for **OC1**. This second tricyclic intermediate, denoted **OC2**, is also an unproductive off-cycle species and it is calculated to be 2.2 kcal/mol more stable than **Int5**.

In the next step, the benzylic alcohol moiety of the ThDP-substrate adduct transfers its proton to Glu28 (via His70) to form **Int6**. The proton transfer is calculated to be endothermic by 8.9 kcal/mol, and the energy of the resulting intermediate can be considered as the effective barrier for this step^1^.

Although it would appear that C-C bond cleavage from **Int6** could give rise to the product benzaldehyde, the calculations indicate that, from an energetic perspective, this transformation is not feasible at this stage as a scan of the energy along the C-C bond results in a constant increase to high values. Instead, another proton transfer was necessary. In this step a proton transfer from His70 to N4′ results in an intermediate, **Int7**, in which the 4′-imino group is positively charged, i.e., the **APH**+ form of ThDP. The reaction energy is −8.1 kcal/mol relative to **Int6**, while the transition state (**TS7**) has an energy of +15.9 kcal/mol compared to **Int4**, the most stable intermediate.

In the penultimate step, the C-C bond between C2α and ThDP is cleaved and benzaldehyde is formed. During the process the N4′-C2 distance successively decreases reduces from 3.25 Å in **Int7** to 3.05 Å in **TS8** and 2.84 Å in the enzyme-product (**EP**) complex. This reduction illustrates the favorable interaction between the positively charged N4' and the increasing negative charge on C2. Relative to **Int7** the calculated energy barrier for this step (**TS8**) is 6.4 kcal/mol. Overall, the enzyme product complex (**EP**) is 2.6 kcal/mol more stable than the enzyme-substrate (**ES)**.

Closure of the catalytic cycle (Scheme 1) and regeneration of the **ES** form requires the release of benzaldehyde from the active site, protonation of the C2 of ThDP and the binding of a new substrate molecule. These transformations involve two binding/release processes, the energy of which is difficult to estimate with the current methodology. Such calculations would be associated with significant errors, and would not contribute greatly to the discussion.

As a final note, it should be mentioned that the potential for His281 being protonated was also considered in these calculations. To explore this possibility both **Int3a** and **TS4a** were re-optimized. The calculations showed that the barrier with protonated His281 was 4 kcal/mol higher than that with neutral His281, suggesting that the latter was more likely.

Overall, according to the calculations summarized in Figure 3, decarboxylation (**TS4a**, Scheme 2) is expected to be the rate-determining step (RDS), with a barrier of 17.4 kcal/mol. However, it should be noted that calculations of steps involving small molecule release, such as the CO_2_ release in **TS4a**, are subject to some uncertainties due to entropic effects. Considering this, and the fact that the barriers of **TS2** (15.2 kcal/mol) and **TS7** (15.9 kcal/mol) are quite close to that of **TS4a** (17.4 kcal/mol), any statement about which of these steps is rate-limiting would be somewhat speculative.

### Comparison with experimental results

In this section, the results of the calculations discussed above will be compared with experimental data available from kinetics, site-directed mutagenesis and X-ray crystallography.

First, the *k*_cat_ for the BFDC reaction has been reported over the range 320–400 s^−1^ (Yep et al., 2008; Bruning et al., 2009). This can be converted to a rate-limiting barrier of *ca*. 14 kcal/mol. Given the errors inherent in both the kinetic measurements and the calculations, this is in good agreement with 17.4 kcal/mol, the highest barrier obtained from our calculations (corresponding to **OC1** → **TS4a**).

To date there have been two significant studies of the individual catalytic steps of the BFDC reaction. As noted in the introduction, the first of these used a non-native substrate, *p*-nitrobenzoylformate, which gave rise to at least two charge transfer complexes as the reaction progressed. These complexes were assigned to *p*-nitroMThDP and the corresponding enamine (Sergienko et al., 2000). Pre-steady-state kinetic analyses indicated that, while decarboxylation was slower than formation of *p*-nitroMThDP, enamine protonation/product release was rate-limiting (Sergienko et al., 2000; Polovnikova et al., 2003). Unfortunately, the non-native substrate renders the kinetic data unsuitable for true comparative purposes and, furthermore, the spectral assignments have subsequently been brought into question (*vide infra*).

The second study used a combined chemical quench/^1^H NMR technique that provided individual rate constants for several of the intermediate steps of the mechanism (Bruning et al., 2009). In this method the reaction is allowed to reach steady-state before being quenched with perchloric acid. Once the protein precipitate is removed the remaining covalently bound ThDP species are characterized by NMR spectroscopy. For the BFDC reaction, three species have been identified: free ThDP, MThDP (**Int2** in the calculations above), and a HBnThDP species. The last represents all intermediates between the enamine and the benzaldehyde formation as these are indistinguishable at low pH (Bruning et al., 2009). Thus, HBnThDP corresponds to **Int4**-**Int7** in our calculations. From the ratios of the intermediates, it was concluded that the initial nucleophilic attack of the ylid on benzoylformate (**TS2**) and the enamine protonation/benzaldehyde formation (**TS7**) were each partially rate-limiting for the overall reaction (Bruning et al., 2009). On the other hand, the decarboxylation step (**TS4a**) was found to be *ca*. 30-fold faster, which corresponds to a difference in energy barriers of *ca*. 2 kcal/mol.

Both sets of experimental results are seemingly in conflict with the calculations described herein that predict that decarboxylation (**TS4a**) will be rate-determining. However, as discussed above, a closer examination of Figure 3 shows that the barrier for **TS4a** is very similar to those for **TS2** and **TS7** (17.4 vs. 15.2 and 15.9 kcal/mol, respectively). Taking into account the margin for error in both experimental and computational approaches, it may reasonably be argued that the calculations and the experimental findings are largely in agreement.

As noted previously, two early papers on the BFDC mechanism suggested that His281 and His70 are involved in the acid-base chemistry, while Ser26 was found to be play a substantial role in essentially all steps, i.e., substrate binding, decarboxylation, enamine protonation, and benzaldehyde formation (Sergienko et al., 2000; Polovnikova et al., 2003). Two factors seem to mitigate against those results. The first is that acid quench/NMR experiments using p-nitrobenzoylformate as a substrate implied that that the charge-transfer complexes may have been wrongly assigned. Second, a saturation mutagenesis study provided strong evidence that, if indeed His70 and His281 were involved in proton transfer reactions, those reactions were certainly not rate limiting (Yep et al., 2008).

Looking at the experimental (structural and kinetic) observations through the lens of the computational study, a broad consensus can again be found. The most obvious conclusion, based on the **ES** structure (Figure 2), is that all three of His70, His281, and Ser26 are involved in the binding of the substrate through hydrogen bonds.

Focusing initially on Ser26, the calculations confirm that it is involved in substrate binding, through a hydrogen bond with the carboxylate group of benzoylformate. Consistent with the experimental observations (Polovnikova et al., 2003), this residue can be seen to play an unambiguous role in the decarboxylation step, where the hydrogen bond has to rotate away in order for the C-C bond cleavage to occur (Figure 4). By forming a hydrogen bond to the benzylic oxygen in all intermediates and transition states after CO_2_ release (Figures 4, 5), Ser26 also contributes to the protonation of the enamine and formation of benzaldehyde.

Unlike the His70Ala mutation, which resulted in a *ca*. 3,500-fold decrease in *k*_cat_ value, replacement of His70 by Thr or Leu reduced the *k*_cat_ value by only one order of magnitude. This result is incompatible with the behavior expected of an acid/base catalyst (Yep et al., 2008). Happily, the calculations are consistent with the mutagenesis results, in that His70 is proposed to act only as a proton relay and, in the proton transfer events (**Int2** → **Int3a**, **Int3b** → **Int4**, **Int5** → **Int6**, and **Int6** → **Int7**) the net proton transfers are from Glu28. Further, the calculations show that, together with the exocyclic NH_2_ moiety of the cofactor, His70 forms part of an oxyanion hole that stabilizes the alkoxide intermediate (**Int2**, Figure 4) formed by the initial nucleophilic attack of the ylid on the substrate. This accords with structural and kinetic studies describing the importance of both His70 and the exocyclic amino group on the formation of MThDP (Fiedler et al., 2002; Polovnikova et al., 2003; Tittmann et al., 2003; Schütz et al., 2005; Wille et al., 2006).

Turning to His281, the computational results suggest that this residue is involved solely in substrate binding and plays no acid/base role in the reactions. Although the H281A variant showed a 170-fold decrease in *k*_cat_ value (Polovnikova et al., 2003), mutation to Trp and Glu saw only a 20-fold decrease in *k*_cat_ (Yep et al., 2008). His281 had been implicated in the protonation of the enamine, but an important mechanistic result of our calculations is that the enamine is protonated by the APH+ form of the cofactor rather than either of the two histidines, as previously suggested (Kluger et al., 2006; Bruning et al., 2009).

A final note concerns the low energy, off-cycle species **OC1** and **OC2** shown in Scheme 2. Such cyclic derivatives of ThDP have never been brought up in the context of the BFDC reaction. However, tricyclic species derived from ThDP have been found in solution (Metzler and Maier, 1962; Washabaugh et al., 1993). Further, species analogous to **OC2** have been observed in the X-ray structures the ThDP-dependent enzymes, acetolactate synthase (Pang et al., 2004), and phosphoketolase (Suzuki et al., 2010). In both cases it was described as a non-productive intermediate.

## Conclusions

In this paper we have investigated the reaction mechanism of benzoylformate decarboxylase using DFT calculations in conjunction with a large model of the active site consisting of more than 300 atoms. The detailed mechanism is presented in Scheme 2 and the calculated energy profile is given in Figure 3. The geometries of the various intermediates and transition states along the reaction path have been characterized and are shown in Figures 2, 4, 5.

The calculated overall energy barrier is found to be in good agreement with the experimental kinetic data. A very interesting finding of the current study is that the ThDP cofactor can form low-energy off-cycle cyclic intermediates that may have implication for the kinetics of the enzyme. In addition, the roles of the various active site residues suggested on the basis of the calculations are discussed and compared to available mutagenesis experiments.

We believe that the detailed mechanistic insight provided by the present calculations will be very valuable also for the understanding of the mechanisms of other ThDP-dependent enzymes. The information can also be used in the design of BFDC variants for biocatalytic applications.

## Author contributions

FP, XS, MM, and FH designed the project. FP performed the calculations. FP, XS, MM, and FH analyzed the results. FP, MM, and FH wrote the paper.

### Conflict of interest statement

The authors declare that the research was conducted in the absence of any commercial or financial relationships that could be construed as a potential conflict of interest.

## Footnotes

^1^The transition state geometries corresponding to the barrier-less transitions were successfully optimized and their electronic energies in the gas-phase were somewhat higher than the connecting intermediates. However, after adding the basis set and solvation corrections, the energies were lowered and became slightly lower than the connecting intermediates.
