# Supplementary material for: A Theoretical Study of the Benzoylformate Decarboxylase Reaction Mechanism
Source: Front Chem. 2018 Jun 26;6:205. doi: 10.3389/fchem.2018.00205 (PMC6028569; doi:10.3389/fchem.2018.00205)
Supplement: Supplementary file 1 [file Table_1.PDF]

Supporting Information

A Theoretical Study of the Benzoylformate Decarboxylase Reaction Mechanism

Ferran Planas[a], Xiang Sheng[a], Michael J McLeish[b] and Fahmi Himo[a*]*

[a]Department of Organic Chemistry, Arrhenius Laboratory, Stockholm University, SE-10691, Stockholm (Sweden)

[b]Department of Chemistry and Chemical Biology, Indiana University-Purdue University Indianapolis, IN 46202, Indianapolis (USA)

E-mail: fahmi.himo@su.se; mcleish@iupui.edu

Table of contents:

1. Calculated Energies and Energy Corrections. S2

2. Cartesian Coordinates of Stationary Points S3

1. Calculated Energies and Energy Corrections.

Stationary point	E_{el} (a.u.)	E_{bb} (a.u.)	E_{solv} (a.u.)	ZPE (a.u.)	E_{tot} (a.u.)	E_{rel} (kcal/mol)
**ES**	-6914.00520	-6915.86876	-6914.13032	2.63584	-6913.35804	**0.0**
**OC1**	-6914.02416	-6915.88291	-6914.14564	2.63779	-6913.36581	**-5.4**
**TS1**	-6913.99158	-6915.85211	-6914.11524	2.63148	-6913.34428	**8.6**
**Int1**	-6914.00338	-6915.86418	-6914.12611	2.63507	-6913.35183	**3.9**
**TS2**	-6913.99122	-6915.85191	-6914.11710	2.63530	-6913.34249	**9.8**
**Int2**	-6914.00133	-6915.87149	-6914.13898	2.63629	-6913.36470	**-4.2**
**TS3b**	-6913.98007	-6915.84747	-6914.10130	2.63215	-6913.33655	**13.5**
**Int3b**	-6913.98656	-6915.85444	-6914.10987	2.61976	-6913.34641	**-3.8**
**Int3a**	-6914.00267	-6915.86530	-6914.13949	2.63654	-6913.36559	**-4.7**
**TS4a**	-6913.97611	-6915.84236	-6914.10640	2.63382	-6913.33884	**12.0**
**Int4-CO_2**	-6913.98462	-6915.85382	-6914.11110	2.63342	-6913.34688	**7.0**
**Int4**	-6914.00138	-6915.86840	-6914.12559	2.62296	-6913.35806	**-11.1**
**TS5**	-6913.97667	-6915.84279	-6914.10174	2.61823	-6913.33804	**1.5**
**Int5**	-6913.99583	-6915.86266	-6914.12341	2.62441	-6913.35424	**-8.7**
**OC2**	-6913.98662	-6915.84894	-6914.11621	2.63585	-6913.34268	**-10.9**
**Int6**	-6913.99089	-6915.85499	-6914.11277	2.62519	-6913.34007	**0.2**
**TS7**	-6913.96752	-6915.83509	-6914.09672	2.61993	-6913.33277	**4.8**
**Int7**	-6913.98010	-6915.84968	-6914.11681	2.62129	-6913.35351	**-8.3**
**TS8**	-6913.97689	-6915.84281	-6914.10969	2.62102	-6913.34299	**-1.7**
**EP**	-6913.98503	-6915.85104	-6914.11138	2.62125	-6913.34456	**-2.6**

E_{el} = Energy of the optimized geometry at B3LYP/6-31G(d,p) level.

E_{bb} = Single-point energy at the B3LYP/6-311+G(2d,2p) level.

E_{solv} = Single-point solvation energy calculated at the B3LYP/6-31G(d,p) level using the SMD method with ε=4.

E_{tot} = E_{bb} + (E_{solv} – E_{el}) + ZPE

E_{rel} = Final relative energies

2. Cartesian Coordinates of Stationary Points

ES

C	11.4745	5.2491	-0.5542
C	11.0395	4.0006	-1.3344
C	9.8386	3.3377	-0.7329
C	8.5601	3.2468	-1.2379
N	9.8973	2.7288	0.5079
C	8.6748	2.2766	0.7369
N	7.8264	2.5719	-0.2867
H	10.6853	6.0081	-0.5662
H	11.8711	3.2848	-1.3558
H	10.8232	4.2650	-2.3767
H	8.1194	3.5988	-2.1583
H	8.3461	1.7067	1.5959
H	6.8681	2.2259	-0.3663
C	-9.4427	2.3967	-4.4220
C	-8.1971	2.9033	-3.6769
C	-7.3096	1.7810	-3.1866
C	-6.5489	1.0241	-4.0890
C	-7.2356	1.4477	-1.8297
C	-5.7469	-0.0276	-3.6558
C	-6.4280	0.4038	-1.3749
C	-5.6787	-0.3384	-2.2924
O	-4.8784	-1.3845	-1.9250
H	-10.0514	1.7651	-3.7667
H	-8.5097	3.5168	-2.8241
H	-7.6268	3.5636	-4.3437
H	-6.5751	1.2683	-5.1488
H	-7.7895	2.0069	-1.0796
H	-5.1498	-0.6060	-4.3531
H	-6.3695	0.2212	-0.3056
H	-4.7177	-1.3988	-0.9464
C	-0.3575	-6.5269	-5.2229
C	-0.4230	-6.2001	-3.7256
C	-1.7849	-5.7848	-3.1975
C	-2.0821	-5.9352	-1.8363
C	-2.7758	-5.2193	-4.0127
C	-3.3120	-5.5513	-1.3064
C	-4.0117	-4.8299	-3.4998
C	-4.2911	-5.0046	-2.1426
O	-5.5381	-4.6682	-1.7023
H	-0.5612	-5.6459	-5.8400
H	-0.0831	-7.0718	-3.1537
H	0.3063	-5.4080	-3.5026
H	-1.3384	-6.3686	-1.1735
H	-2.5896	-5.0808	-5.0728
H	-3.5133	-5.6805	-0.2460
H	-4.7696	-4.3852	-4.1348
H	-5.5543	-4.6280	-0.7342
C	4.8954	-4.1754	-5.6242
C	5.1305	-4.9627	-4.3577
O	6.2075	-5.4991	-4.1016
C	5.4985	-2.7664	-5.5227
H	5.3683	-4.7215	-6.4455
H	6.5784	-2.8249	-5.3688
H	5.0664	-2.2150	-4.6823
H	5.3097	-2.1971	-6.4377
N	4.0793	-4.9866	-3.5023
C	4.2344	-5.5279	-2.1683
C	2.9337	-5.4678	-1.3796
C	2.3362	-4.0617	-1.1943
C	1.1097	-4.1618	-0.2876
C	3.3610	-3.0570	-0.6516
H	3.2662	-4.4261	-3.7041
H	5.0212	-4.9810	-1.6402
H	3.1313	-5.8919	-0.3874
H	2.1845	-6.1169	-1.8522
H	1.9860	-3.6922	-2.1727
H	1.3990	-4.4649	0.7248
H	0.5827	-3.2147	-0.1977
H	0.3892	-4.8933	-0.6669
H	4.1979	-2.9035	-1.3403
H	3.7751	-3.4051	0.3012
H	2.9131	-2.0767	-0.4723
C	9.1723	-5.4223	-0.9215
C	7.8212	-4.6960	-0.9311
C	7.9374	-3.2218	-1.2532
C	7.4288	-2.7150	-2.4543
C	8.5689	-2.3342	-0.3712
C	7.5358	-1.3561	-2.7589
C	8.6781	-0.9774	-0.6710
C	8.1563	-0.4811	-1.8679
H	9.8551	-4.9883	-0.1837
H	7.3394	-4.8181	0.0484
H	7.1760	-5.1733	-1.6732
H	6.9750	-3.4066	-3.1543
H	8.9692	-2.7085	0.5673
H	7.1461	-0.9835	-3.7024
H	9.1617	-0.3004	0.0246
H	8.2421	0.5758	-2.0948
C	-2.8677	0.7102	0.6451
C	-1.6629	0.9257	0.0604
C	-1.4285	2.1780	-0.6552
N	-0.2425	2.4519	-1.0994
N	-2.5298	3.0398	-0.8064
C	-3.6660	2.7580	-0.2186
N	-3.8666	1.6338	0.5216
S	1.4900	-0.5505	-3.0829
C	1.0811	0.0603	-1.5547
C	-4.8245	3.7032	-0.3201
N	0.0080	-0.5193	-1.0511
C	-0.5853	-1.4879	-1.8734
C	-1.8313	-2.1825	-1.4499
C	0.1081	-1.6341	-3.0395
C	-0.2092	-2.5053	-4.2113
C	-0.5446	-0.0438	0.2482
H	1.6482	0.7823	-0.9976
H	-0.2038	3.3568	-1.5736
H	-0.8546	-0.9223	0.8123
H	0.2925	0.4327	0.7575
H	-5.7300	3.1665	-0.6075
H	-5.0263	4.1651	0.6513
H	-4.6037	4.4890	-1.0418
H	-3.1131	-0.1737	1.2224
H	-1.7942	-2.4735	-0.3980
H	-1.9805	-3.0820	-2.0448
H	-2.6971	-1.5367	-1.6051
H	-1.0124	-3.1981	-3.9582
H	0.6601	-3.0917	-4.5277
C	1.8120	-2.8502	7.3370
C	1.0839	-1.5210	7.5807
C	1.6969	-0.3649	6.8122
O	1.5731	-0.5206	5.4892
O	2.2260	0.6001	7.3314
C	-8.2808	2.8322	4.0679
C	-6.8917	2.5654	3.4812
C	-6.9157	2.0067	2.0455
O	-7.6721	2.5667	1.2127
O	-6.1259	1.0373	1.7983
H	-8.2293	3.4437	4.9749
H	-8.9071	3.3394	3.3346
H	-6.3120	1.8888	4.1147
H	-6.3287	3.5088	3.4306
C	-0.7921	1.9686	5.4605
C	-0.7288	1.4445	4.0214
C	0.5391	1.8252	3.3241
C	0.7150	2.5660	2.1808
N	1.7894	1.4315	3.7910
C	2.6834	1.9162	2.9378
N	2.0739	2.6181	1.9554
H	0.0776	1.6573	6.0419
H	-0.8397	0.3554	4.0188
H	-1.5747	1.8288	3.4419
H	0.0161	3.0284	1.5046
H	3.7499	1.7584	2.9796
H	2.5132	2.9733	1.1013
C	5.5323	7.0787	3.0243
C	5.6074	5.7767	3.8291
C	6.0687	4.5368	3.0395
C	6.0756	3.3035	3.9549
C	5.1967	4.2933	1.8025
H	4.7780	7.0210	2.2339
H	4.6177	5.5623	4.2573
H	6.2823	5.9180	4.6836
H	7.0993	4.7093	2.6962
H	5.0770	3.1299	4.3747
H	6.3764	2.3962	3.4208
H	6.7605	3.4408	4.7991
H	4.1377	4.2750	2.0798
H	5.4420	3.3393	1.3305
H	5.3234	5.0732	1.0463
H	-0.5402	-1.9062	-5.0667
H	-8.7763	1.8899	4.3285
H	11.6671	4.9862	0.4895
H	12.3840	5.6914	-0.9766
H	-10.0650	3.2275	-4.7720
H	-9.1591	1.7943	-5.2914
H	-0.8150	3.0633	5.4725
H	-1.6932	1.6044	5.9641
H	6.4936	7.3054	2.5484
H	5.2714	7.9246	3.6691
H	0.0373	-1.6083	7.2632
H	1.0959	-1.2532	8.6395
H	1.8835	-3.0719	6.2685
H	1.2903	-3.6734	7.8344
H	2.8328	-2.8089	7.7283
H	9.0456	-6.4832	-0.6816
H	9.6524	-5.3499	-1.9024
H	4.5833	-6.5622	-2.2530
H	0.6394	-6.8897	-5.4898
H	-1.0863	-7.2979	-5.4914
H	3.8234	-4.1097	-5.8469
H	1.8167	0.3273	4.9448
H	-4.7931	1.4379	0.9868
C	2.2289	6.9954	-2.7962
C	0.9785	6.2413	-2.3846
O	0.8112	5.0523	-2.6579
N	0.0482	6.9506	-1.6841
C	-1.2819	6.3816	-1.5397
C	-1.9260	6.1847	-2.9236
O	-1.7353	7.0014	-3.8229
N	-2.6928	5.0798	-3.0360
C	-3.2511	4.6847	-4.3233
C	-3.3314	3.1606	-4.4405
C	-1.9940	2.4217	-4.2628
C	-2.2141	0.9081	-4.3404
C	-0.9217	2.8852	-5.2546
C	3.7606	2.0362	-2.6029
C	2.9341	2.7946	-3.4484
C	2.9701	2.5696	-4.8215
C	3.8317	1.6059	-5.3536
C	4.6611	0.8593	-4.5127
C	4.6228	1.0620	-3.1352
C	3.6576	2.2441	-1.1396
O	3.1034	3.2297	-0.6533
C	4.2136	1.1544	-0.2181
O	5.4703	1.1535	-0.1006
O	3.3851	0.4016	0.3395
H	3.1010	6.4024	-2.5112
H	2.2293	7.0961	-3.8853
H	-1.2270	5.4405	-0.9896
H	-1.8907	7.0786	-0.9571
H	-4.0486	2.7769	-3.7040
H	-3.7535	2.9186	-5.4258
H	-1.6133	2.6384	-3.2622
H	-0.0097	2.2919	-5.1292
H	-1.2603	2.7711	-6.2929
H	-0.6508	3.9320	-5.0902
H	-2.6067	0.6135	-5.3226
H	-2.9286	0.5693	-3.5840
H	-1.2733	0.3744	-4.1798
H	-2.6218	5.1201	-5.1040
H	2.2628	3.5347	-3.0229
H	2.3206	3.1444	-5.4738
H	3.8580	1.4365	-6.4265
H	5.3373	0.1214	-4.9307
H	5.2678	0.4888	-2.4773
C	-5.0447	-3.1354	3.4494
C	-3.6208	-2.6093	3.6442
O	-3.3805	-1.4086	3.7536
C	-5.9259	-2.1889	2.6154
C	-5.5922	-2.2256	1.1392
N	-6.5014	-2.7813	0.3088
O	-4.5180	-1.7781	0.7014
N	-2.6439	-3.5454	3.8057
C	-1.2881	-3.1194	4.1174
C	-0.5709	-2.5592	2.8868

O	−0.9003	−2.8861	1.7421
C	−0.5933	−4.4321	4.5340
C	−1.2678	−5.4634	3.6163
C	−2.7277	−4.9848	3.5265
N	0.4825	−1.7779	3.1882
C	1.5847	−1.5421	2.2843
C	2.8611	−2.1727	2.8515
O	2.8326	−3.2006	3.5280
N	3.9887	−1.4914	2.5406
C	5.3066	−1.9696	2.9230
C	5.7198	−1.6349	4.3645
O	6.6999	−2.1880	4.8576
C	6.3666	−1.3514	2.0002
O	6.3847	0.0585	2.1383
N	5.0049	−0.6692	4.9847
C	5.4790	−0.0501	6.2079
H	−6.9776	−2.4507	2.7605
H	−5.7719	−1.1523	2.9311
H	−5.0513	−4.1411	3.0213
H	−0.8103	−4.6411	5.5868
H	0.4898	−4.3766	4.4022
H	−1.1909	−6.4879	3.9886
H	−0.8113	−5.4196	2.6246
H	−3.3634	−5.4880	4.2670
H	−3.1487	−5.1686	2.5330
H	−1.3024	−2.3683	4.9113
H	1.7358	−0.4787	2.1073
H	1.3509	−2.0181	1.3331
H	7.3356	−1.7664	2.2938
H	6.1664	−1.6426	0.9625
H	6.0576	0.4434	1.2939
H	5.3379	−3.0605	2.8418
H	6.2370	0.7157	5.9997
H	2.3015	7.9875	−2.3445
H	−4.2535	5.1153	−4.4510
H	−2.6497	4.3853	−2.2835
H	0.0965	7.9553	−1.7659
H	5.9348	−0.8196	6.8330
H	4.6369	0.4048	6.7323
H	4.2601	−0.2342	4.4635
H	0.6808	−1.5762	4.1651
H	−7.4003	−3.1444	0.6227
H	−6.3308	−2.6933	−0.6871
H	3.9228	−0.7308	1.8603
H	−5.4770	−3.2233	4.4537
C	−11.0188	−4.9732	1.6547
C	−10.0143	−4.3290	0.7225
O	−8.8317	−4.2061	1.0567
C	−11.2236	−4.0478	2.8582
N	−10.4984	−3.8461	−0.4422
C	−9.6675	−3.0883	−1.3609
C	−8.8135	7.0937	3.2660
C	−8.5852	5.8894	2.3628
C	−8.2244	6.2596	0.9241
N	−7.8512	5.1942	0.1697
O	−8.2834	7.4111	0.4989
H	−10.2649	−3.8527	3.3439
H	−11.9006	−4.4969	3.5903
H	−11.6474	−3.0876	2.5478
H	−11.9690	−5.1755	1.1478
H	−8.7939	−3.6758	−1.6587
H	−7.7950	5.2462	2.7601
H	−9.4826	5.2598	2.3251
H	−7.8062	4.2433	0.5506
H	−7.6100	5.3667	−0.7940
H	−9.5924	7.7425	2.8574
H	−9.1098	6.7737	4.2699
H	−7.9055	7.6977	3.3501
H	−10.6031	−5.9310	1.9809
H	−9.3140	−2.1562	−0.9082
H	−10.2487	−2.8482	−2.2516
H	−11.4833	−3.9374	−0.6329

TS1

C	11.4253	5.3191	−0.3911
C	10.8531	4.2001	−1.2743
C	9.6624	3.5149	−0.6717
C	8.3853	3.4017	−1.1775
N	9.7425	2.8631	0.5460
C	8.5349	2.3657	0.7613
N	7.6748	2.6692	−0.2504
H	10.6904	6.1188	−0.2507
H	11.6405	3.4555	−1.4530
H	10.5757	4.6007	−2.2571
H	7.9315	3.7699	−2.0851
H	8.2239	1.7592	1.6018
H	6.7235	2.3045	−0.3357
C	−9.4362	2.3290	−4.4507
C	−8.2290	2.8651	−3.6600
C	−7.2900	1.7832	−3.1665
C	−6.5387	1.0156	−4.0688
C	−7.1353	1.5144	−1.8020
C	−5.6715	0.0202	−3.6293
C	−6.2642	0.5246	−1.3406
C	−5.5237	−0.2274	−2.2583
O	−4.6598	−1.2202	−1.8895
H	−10.0367	1.6567	−3.8294
H	−8.5879	3.4449	−2.8018
H	−7.6761	3.5686	−4.2972
H	−6.6235	1.2071	−5.1363
H	−7.6812	2.0814	−1.0533
H	−5.0844	−0.5641	−4.3296
H	−6.1620	0.3751	−0.2699
H	−4.5341	−1.2649	−0.9079
C	−0.2705	−6.5107	−5.2626
C	−0.3822	−6.3770	−3.7407
C	−1.7224	−5.8918	−3.2167
C	−2.0385	−6.0519	−1.8602
C	−2.6727	−5.2579	−4.0282
C	−3.2485	−5.6089	−1.3322
C	−3.8880	−4.8076	−3.5164
C	−4.1865	−4.9889	−2.1653
O	−5.4149	−4.5845	−1.7268
H	−0.3637	−5.5411	−5.7625
H	−0.1599	−7.3465	−3.2783
H	0.4081	−5.7009	−3.3853
H	−1.3233	−6.5361	−1.2006
H	−2.4697	−5.1091	−5.0834
H	−3.4676	−5.7488	−0.2766
H	−4.6137	−4.3073	−4.1480
H	−5.4311	−4.5391	−0.7591
C	4.9655	−4.1119	−5.6004
C	5.1841	−4.9214	−4.3434
O	6.2576	−5.4619	−4.0834
C	5.4329	−2.6582	−5.4249
H	5.5338	−4.5909	−6.4021
H	6.5042	−2.6243	−5.2136
H	4.9094	−2.1685	−4.5981
H	5.2430	−2.0799	−6.3339
N	4.1192	−4.9681	−3.5006
C	4.2888	−5.5021	−2.1626
C	2.9918	−5.5133	−1.3674
C	2.3003	−4.1486	−1.1930
C	1.1494	−4.3059	−0.1980
C	3.2632	−3.0339	−0.7618
H	3.3292	−4.3668	−3.6799
H	5.0452	−4.9168	−1.6289
H	3.2295	−5.9103	−0.3726
H	2.2814	−6.2177	−1.8208
H	1.8575	−3.8532	−2.1574
H	1.5353	−4.5470	0.7998
H	0.5575	−3.3987	−0.1036
H	0.4667	−5.1076	−0.5003
H	4.0665	−2.8724	−1.4879
H	3.7333	−3.2738	0.1984
H	2.7452	−2.0786	−0.6423
C	9.2156	−5.3672	−0.8758
C	7.8562	−4.6564	−0.8813
C	7.9522	−3.1752	−1.1787
C	7.4004	−2.6509	−2.3530
C	8.5986	−2.2964	−0.2987
C	7.4797	−1.2851	−2.6327
C	8.6810	−0.9323	−0.5745
C	8.1157	−0.4191	−1.7440
H	9.8895	−4.9381	−0.1271
H	7.3707	−4.8008	0.0935
H	7.2193	−5.1293	−1.6338
H	6.9335	−3.3333	−3.0535
H	9.0299	−2.6828	0.6211
H	7.0520	−0.8986	−3.5535
H	9.1742	−0.2620	0.1211
H	8.1762	0.6435	−1.9514
C	−2.7568	0.7383	0.5693
C	−1.5656	0.9661	−0.0462
C	−1.3458	2.2643	−0.6533
N	−0.1661	2.5877	−1.1313
N	−2.4017	3.1584	−0.6955
C	−3.5377	2.8532	−0.1003
N	−3.7364	1.6856	0.5515
S	1.4031	−0.3149	−3.3943
C	0.9206	0.3744	−1.9069
C	−4.6693	3.8336	−0.1038
N	0.0253	−0.4348	−1.3323
C	−0.3777	−1.5786	−2.0409
C	−1.5216	−2.4161	−1.5712
C	0.3167	−1.6844	−3.2099
C	0.1855	−2.7203	−4.2797
C	−0.4643	−0.0452	0.0137
H	0.6764	1.6165	−1.4796
H	−0.0813	3.5174	−1.5456
H	−0.7763	−0.9435	0.5450
H	0.4052	0.3730	0.5236
H	−5.6307	3.3193	−0.1192
H	−4.6398	4.4370	0.8103
H	−4.5766	4.5080	−0.9554
H	−2.9998	−0.1797	1.0931
H	−1.4895	−2.6137	−0.4992
H	−1.5238	−3.3750	−2.0865
H	−2.4658	−1.9119	−1.8007
H	−0.5422	−3.4775	−3.9867
H	1.1395	−3.2228	−4.4789
C	1.7695	−2.9338	7.3477
C	1.0496	−1.6035	7.6067
C	1.6387	−0.4459	6.8217
O	1.5223	−0.6227	5.5015
O	2.1409	0.5399	7.3296
C	−8.3447	2.6948	4.0517
C	−6.8926	2.4892	3.6149
C	−6.7295	2.0289	2.1566
O	−7.4123	2.6042	1.2728
O	−5.8601	1.1192	1.9457
H	−8.4099	3.2513	4.9924
H	−8.8969	3.2346	3.2834
H	−6.3705	1.7847	4.2673
H	−6.3508	3.4434	3.6868
C	−0.8602	1.8805	5.4955
C	−0.8046	1.3327	4.0663
C	0.4502	1.7237	3.3536
C	0.6064	2.4792	2.2184
N	1.7086	1.3304	3.7965
C	2.5870	1.8273	2.9332
N	1.9609	2.5396	1.9697
H	0.0212	1.5925	6.0716
H	−0.8958	0.2420	4.0829
H	−1.6638	1.6945	3.4913
H	−0.1059	2.9700	1.5788
H	3.6540	1.6689	2.9530
H	2.3786	2.8835	1.0973
C	5.4400	7.0657	3.1571
C	5.4698	5.7336	3.9144
C	5.9027	4.5079	3.0871
C	5.8938	3.2514	3.9703
C	5.0183	4.3137	1.8491
H	4.6966	7.0563	2.3540
H	4.4710	5.5331	4.3280
H	6.1414	5.8259	4.7782
H	6.9340	4.6711	2.7412
H	4.8920	3.0780	4.3824
H	6.1890	2.3544	3.4163
H	6.5773	3.3616	4.8197
H	3.9603	4.2990	2.1322
H	5.2473	3.3719	1.3443
H	5.1516	5.1157	1.1170
H	−0.1538	−2.2758	−5.2218
H	−8.8440	1.7304	4.1995
H	11.6753	4.9210	0.5959
H	12.3282	5.7547	−0.8333
H	−10.0797	3.1464	−4.7931
H	−9.1097	1.7637	−5.3297
H	−0.8993	2.9749	5.4890
H	−1.7497	1.5118	6.0163
H	6.4140	7.2840	2.7033

H	5.1913	7.8943	3.8286
H	−0.0052	−1.6907	7.3166
H	1.0878	−1.3334	8.6644
H	1.8051	−3.1638	6.2792
H	1.2656	−3.7534	7.8688
H	2.8033	−2.8898	7.7030
H	9.0992	−6.4331	−0.6535
H	9.7004	−5.2741	−1.8527
H	4.6924	−6.5152	−2.2514
H	0.7029	−6.9275	−5.5366
H	−1.0486	−7.1687	−5.6624
H	3.9066	−4.1307	−5.8872
H	1.7502	0.2245	4.9485
H	−4.6594	1.4785	1.0606
C	2.1832	7.0094	−2.6900
C	0.9067	6.2960	−2.2947
O	0.7311	5.0974	−2.5247
N	−0.0369	7.0470	−1.6639
C	−1.3787	6.5043	−1.5264
C	−1.9935	6.2495	−2.9140
O	−1.8222	7.0494	−3.8308
N	−2.7191	5.1143	−3.0057
C	−3.2651	4.6675	−4.2819
C	−3.3434	3.1408	−4.3386
C	−1.9983	2.4114	−4.1798
C	−2.2253	0.8985	−4.1111
C	−0.9919	2.7824	−5.2748
C	3.7093	2.0673	−2.5995
C	2.9475	2.8726	−3.4615
C	3.0898	2.7317	−4.8390
C	4.0000	1.8072	−5.3599
C	4.7700	1.0187	−4.5018
C	4.6217	1.1372	−3.1219
C	3.4888	2.1850	−1.1376
O	2.8784	3.1367	−0.6478
C	4.0640	1.1057	−0.2182
O	5.3214	1.1895	−0.0809
O	3.2800	0.3084	0.3315
H	3.0326	6.4181	−2.3401
H	2.2317	7.0539	−3.7818
H	−1.3527	5.5921	−0.9272
H	−1.9909	7.2396	−0.9975
H	−4.0366	2.7852	−3.5657
H	−3.7984	2.8580	−5.2974
H	−1.5538	2.7186	−3.2302
H	−0.0613	2.2201	−5.1452
H	−1.3883	2.5557	−6.2731
H	−0.7366	3.8456	−5.2397
H	−2.7147	0.5322	−5.0224
H	−2.8625	0.6248	−3.2654
H	−1.2778	0.3656	−4.0076
H	−2.6323	5.0739	−5.0755
H	2.2453	3.5877	−3.0436
H	2.4860	3.3400	−5.5054
H	4.1098	1.7018	−6.4355
H	5.4832	0.3094	−4.9090
H	5.2166	0.5270	−2.4512
C	−5.0539	−3.2396	3.4033
C	−3.6324	−2.7348	3.6580
O	−3.4047	−1.5515	3.9004
C	−5.8936	−2.2317	2.5954
C	−5.5134	−2.1906	1.1295
N	−6.4007	−2.7019	0.2482
O	−4.4265	−1.7250	0.7459
N	−2.6471	−3.6736	3.7115
C	−1.2898	−3.2612	4.0295
C	−0.6090	−2.5917	2.8325
O	−0.9755	−2.8206	1.6749
C	−0.5763	−4.5991	4.3099
C	−1.2466	−5.5440	3.2993
C	−2.7103	−5.0650	3.2446
N	0.4624	−1.8526	3.1664
C	1.5590	−1.5807	2.2643
C	2.8447	−2.1839	2.8420
O	2.8316	−3.2163	3.5136
N	3.9577	−1.4716	2.5518
C	5.2855	−1.9276	2.9283
C	5.7010	−1.6038	4.3722
O	6.6972	−2.1443	4.8481
C	6.3285	−1.2845	2.0026
O	6.3135	0.1258	2.1349
N	4.9705	−0.6684	5.0196
C	5.4216	−0.0929	6.2737
H	−6.9535	−2.4814	2.6920
H	−5.7252	−1.2207	2.9787
H	−5.0648	−4.2217	2.9241
H	−0.7849	−4.9121	5.3383
H	0.5053	−4.5168	4.1791
H	−1.1685	−6.5974	3.5797
H	−0.7854	−5.4131	2.3178
H	−3.3565	−5.6641	3.8988
H	−3.1076	−5.1168	2.2257
H	−1.2925	−2.5827	4.8860
H	1.6834	−0.5151	2.0865
H	1.3442	−2.0583	1.3093
H	7.3081	−1.6741	2.2944
H	6.1329	−1.5836	0.9663
H	5.9506	0.4996	1.2980
H	5.3376	−3.0172	2.8383
H	6.1650	0.6968	6.1059
H	2.2535	8.0219	−2.2862
H	−4.2669	5.0919	−4.4309
H	−2.6409	4.4409	−2.2412
H	0.0370	8.0479	−1.7674
H	5.8884	−0.8792	6.8692
H	4.5658	0.3235	6.8077
H	4.2018	−0.2466	4.5224
H	0.6755	−1.7171	4.1508
H	−7.3113	−3.0648	0.5279
H	−6.1969	−2.5827	−0.7376
H	3.8750	−0.7311	1.8490
H	−5.5162	−3.3663	4.3896
C	−10.8226	−5.1308	1.6061
C	−9.8848	−4.3836	0.6807
O	−8.7458	−4.0786	1.0467
C	−11.2200	−4.1978	2.7548
N	−10.3899	−4.0155	−0.5170
C	−9.6390	−3.1860	−1.4427
C	−8.9068	6.9590	3.2852
C	−8.6718	5.7845	2.3457
C	−8.3366	6.2006	0.9135
N	−7.8597	5.1792	0.1555
O	−8.5037	7.3430	0.4941
H	−10.3251	−3.8365	3.2665
H	−11.8526	−4.7152	3.4814
H	−11.7711	−3.3290	2.3817
H	−11.7077	−5.4966	1.0734
H	−8.7282	−3.6958	−1.7732
H	−7.8727	5.1357	2.7142
H	−9.5658	5.1500	2.2969
H	−7.7194	4.2430	0.5428
H	−7.6419	5.3712	−0.8099
H	−9.6825	7.6192	2.8895
H	−9.2102	6.6079	4.2764
H	−7.9995	7.5602	3.3969
H	−10.2832	−5.9996	1.9944
H	−9.3492	−2.2398	−0.9757
H	−10.2588	−2.9740	−2.3143
H	−11.3338	−4.2804	−0.7480

Int1

C	11.3963	5.3047	−0.3983
C	10.8238	4.1850	−1.2800
C	9.6300	3.5032	−0.6792
C	8.3667	3.3498	−1.2093
N	9.6946	2.8912	0.5596
C	8.4918	2.3773	0.7636
N	7.6494	2.6324	−0.2758
H	10.6640	6.1077	−0.2634
H	11.6109	3.4387	−1.4546
H	10.5505	4.5826	−2.2651
H	7.9266	3.6793	−2.1382
H	8.1734	1.7925	1.6165
H	6.7066	2.2469	−0.3702
C	−9.4414	2.2637	−4.5413
C	−8.2642	2.8843	−3.7671
C	−7.2985	1.8573	−3.2142
C	−6.5306	1.0608	−4.0765
C	−7.1451	1.6578	−1.8380
C	−5.6577	0.0952	−3.5878
C	−6.2676	0.6991	−1.3261
C	−5.5223	−0.0932	−2.2059
O	−4.6688	−1.0736	−1.7920
H	−10.0220	1.5995	−3.8929
H	−8.6521	3.4931	−2.9425
H	−7.7291	3.5737	−4.4341
H	−6.6120	1.2024	−5.1519
H	−7.7086	2.2516	−1.1250
H	−5.0617	−0.5170	−4.2562
H	−6.1684	0.5944	−0.2505
H	−4.5582	−1.0880	−0.8050
C	−0.2757	−6.5913	−5.1643
C	−0.4241	−6.4318	−3.6477
C	−1.7792	−5.9562	−3.1521
C	−2.0635	−5.9843	−1.7792
C	−2.7725	−5.4549	−4.0029
C	−3.2788	−5.5327	−1.2717
C	−3.9952	−4.9983	−3.5117
C	−4.2566	−5.0360	−2.1413
O	−5.4845	−4.6146	−1.7138
H	−0.3950	−5.6357	−5.6845
H	−0.1928	−7.3888	−3.1632
H	0.3444	−5.7339	−3.2885
H	−1.3169	−6.3669	−1.0888
H	−2.5967	−5.4122	−5.0726
H	−3.4687	−5.5642	−0.2017
H	−4.7539	−4.6016	−4.1774
H	−5.4741	−4.4652	−0.7554
C	4.9635	−4.2003	−5.5057
C	5.1833	−4.9893	−4.2352
O	6.2496	−5.5410	−3.9671
C	5.1404	−2.6873	−5.2906
H	5.6835	−4.5630	−6.2430
H	6.1672	−2.4591	−4.9964
H	4.4756	−2.3044	−4.5102
H	4.9216	−2.1394	−6.2119
N	4.1150	−5.0162	−3.3964
C	4.2649	−5.5386	−2.0517
C	2.9608	−5.4911	−1.2683
C	2.3165	−4.0996	−1.1306
C	1.1173	−4.2028	−0.1875
C	3.3057	−3.0235	−0.6638
H	3.3356	−4.4030	−3.5838
H	5.0395	−4.9733	−1.5230
H	3.1720	−5.8772	−0.2631
H	2.2320	−6.1788	−1.7185
H	1.9249	−3.7893	−2.1121
H	1.4455	−4.4477	0.8297
H	0.5578	−3.2717	−0.1344
H	0.4183	−4.9800	−0.5140
H	4.1387	−2.8950	−1.3625
H	3.7312	−3.2827	0.3124
H	2.8205	−2.0491	−0.5649
C	9.1837	−5.3867	−0.7367
C	7.8266	−4.6780	−0.8406
C	7.9463	−3.2016	−1.1523
C	7.4927	−2.6914	−2.3739
C	8.5239	−2.3140	−0.2341
C	7.6001	−1.3288	−2.6611
C	8.6331	−0.9538	−0.5174
C	8.1657	−0.4536	−1.7347
H	9.8074	−4.9440	0.0468
H	7.2825	−4.8062	0.1051
H	7.2357	−5.1620	−1.6228
H	7.0777	−3.3806	−3.1012
H	8.8791	−2.6918	0.7212
H	7.2483	−0.9485	−3.6161
H	9.0702	−0.2767	0.2083
H	8.2457	0.6062	−1.9496
C	−2.5483	0.6801	0.5488
C	−1.3902	0.9125	−0.1405
C	−1.2505	2.2064	−0.7477
N	−0.1099	2.5990	−1.2994
N	−2.2915	3.0907	−0.7339
C	−3.3941	2.7784	−0.0719
N	−3.5419	1.6107	0.5864
S	1.5518	−0.6626	−3.5086
C	1.2174	0.1440	−2.0220
C	−4.5162	3.7642	−0.0101
N	0.1855	−0.5153	−1.4630
C	−0.3949	−1.6092	−2.1469
C	−1.6201	−2.2845	−1.6248

C	0.2615	−1.8445	−3.3145
C	−0.0221	−2.8762	−4.3574
C	−0.2585	−0.0765	−0.1209
H	0.6542	1.9213	−1.4630
H	−0.0083	3.5325	−1.7005
H	−0.5423	−0.9520	0.4633
H	0.6268	0.3723	0.3340
H	−5.4814	3.2616	0.0631
H	−4.4049	4.3879	0.8843
H	−4.4837	4.4184	−0.8819
H	−2.7414	−0.2345	1.0972
H	−1.5418	−2.5374	−0.5655
H	−1.8047	−3.2078	−2.1727
H	−2.4951	−1.6427	−1.7609
H	−0.8620	−3.5062	−4.0617
H	0.8440	−3.5274	−4.5274
C	1.6884	−2.8271	7.4034
C	0.9812	−1.4875	7.6481
C	1.5652	−0.3442	6.8385
O	1.4528	−0.5506	5.5224
O	2.0572	0.6571	7.3259
C	−8.4021	2.7562	3.9612
C	−6.9022	2.6065	3.6835
C	−6.5436	2.1203	2.2677
O	−7.2082	2.5590	1.2950
O	−5.5407	1.3369	2.1877
H	−8.5894	3.3699	4.8480
H	−8.9007	3.2090	3.1053
H	−6.4196	1.9481	4.4104
H	−6.4119	3.5859	3.7844
C	−0.9271	1.9602	5.4632
C	−0.8725	1.3880	4.0434
C	0.3815	1.7743	3.3277
C	0.5449	2.5191	2.1864
N	1.6369	1.3860	3.7825
C	2.5200	1.8738	2.9198
N	1.9022	2.5795	1.9460
H	−0.0411	1.6887	6.0402
H	−0.9537	0.2969	4.0804
H	−1.7362	1.7335	3.4648
H	−0.1666	3.0181	1.5513
H	3.5858	1.7099	2.9439
H	2.3273	2.8841	1.0615
C	5.3902	7.1069	3.0864
C	5.4027	5.7784	3.8508
C	5.8503	4.5467	3.0403
C	5.8332	3.2989	3.9358
C	4.9841	4.3370	1.7916
H	4.6641	7.0944	2.2677
H	4.3958	5.5818	4.2463
H	6.0574	5.8749	4.7270
H	6.8857	4.7099	2.7069
H	4.8277	3.1299	4.3406
H	6.1330	2.3961	3.3941
H	6.5090	3.4179	4.7902
H	3.9226	4.3146	2.0606
H	5.2289	3.3940	1.2964
H	5.1209	5.1355	1.0563
H	−0.2722	−2.4114	−5.3177
H	−8.8633	1.7763	4.1286
H	11.6401	4.9087	0.5910
H	12.3028	5.7353	−0.8381
H	−10.1122	3.0378	−4.9288
H	−9.0845	1.6697	−5.3888
H	−0.9745	3.0540	5.4376
H	−1.8119	1.5941	5.9937
H	6.3738	7.3218	2.6523
H	5.1282	7.9391	3.7484
H	−0.0776	−1.5719	7.3717
H	1.0326	−1.2002	8.7006
H	1.7089	−3.0777	6.3391
H	1.1860	−3.6334	7.9463
H	2.7274	−2.7842	7.7435
H	9.0547	−6.4494	−0.5059
H	9.7303	−5.3073	−1.6817
H	4.6320	−6.5672	−2.1238
H	0.7181	−6.9775	−5.4083
H	−1.0191	−7.2868	−5.5671
H	3.9571	−4.4006	−5.8944
H	1.6773	0.2855	4.9562
H	−4.4453	1.4421	1.1887
C	2.1695	6.9646	−2.7791
C	0.9036	6.2450	−2.3661
O	0.7450	5.0386	−2.5753
N	−0.0490	6.9936	−1.7494
C	−1.3878	6.4442	−1.6098
C	−1.9996	6.1851	−2.9983
O	−1.8189	6.9797	−3.9170
N	−2.7336	5.0539	−3.0893
C	−3.2702	4.6017	−4.3694
C	−3.2945	3.0736	−4.4464
C	−1.9272	2.3929	−4.2707
C	−2.0905	0.8715	−4.2130
C	−0.9082	2.8118	−5.3359
C	3.7716	1.9974	−2.6011
C	3.0913	2.8447	−3.4900
C	3.3046	2.7256	−4.8591
C	4.2049	1.7742	−5.3490
C	4.8931	0.9417	−4.4652
C	4.6761	1.0441	−3.0923
C	3.4689	2.1020	−1.1531
O	2.8185	3.0459	−0.6950
C	4.0502	1.0688	−0.1834
O	5.3136	1.1369	−0.0883
O	3.2739	0.3363	0.4583
H	3.0275	6.3885	−2.4251
H	2.2107	6.9909	−3.8718
H	−1.3578	5.5343	−1.0076
H	−2.0039	7.1775	−1.0827
H	−3.9873	2.6848	−3.6892
H	−3.7241	2.7888	−5.4161
H	−1.5176	2.7096	−3.3099
H	0.0402	2.2865	−5.1819
H	−1.2676	2.5746	−6.3454
H	−0.6975	3.8845	−5.2891
H	−2.5250	0.4857	−5.1440
H	−2.7507	0.5704	−3.3937
H	−1.1251	0.3815	−4.0686
H	−2.6529	5.0406	−5.1573
H	2.3882	3.5729	−3.0988
H	2.7625	3.3692	−5.5455
H	4.3686	1.6821	−6.4190
H	5.5970	0.2085	−4.8449
H	5.2145	0.4012	−2.4056
C	−5.1106	−3.1887	3.4220
C	−3.6838	−2.7077	3.6924
O	−3.4477	−1.5368	3.9814
C	−5.9339	−2.1422	2.6439
C	−5.5348	−2.0533	1.1853
N	−6.3409	−2.6602	0.2810
O	−4.4969	−1.4730	0.8290
N	−2.7046	−3.6533	3.6976
C	−1.3430	−3.2611	4.0225
C	−0.6639	−2.5460	2.8501
O	−1.0406	−2.7178	1.6854
C	−0.6353	−4.6134	4.2422
C	−1.3165	−5.5100	3.1959
C	−2.7795	−5.0262	3.1806
N	0.4215	−1.8422	3.2112
C	1.5282	−1.5584	2.3250
C	2.8088	−2.1583	2.9165
O	2.7886	−3.1819	3.6019
N	3.9242	−1.4554	2.6177
C	5.2492	−1.9056	3.0069
C	5.6524	−1.5558	4.4471
O	6.6445	−2.0838	4.9450
C	6.2966	−1.2769	2.0759
O	6.2730	0.1361	2.1746
N	4.9116	−0.6095	5.0666
C	5.3487	−0.0042	6.3096
H	−6.9963	−2.3897	2.7178
H	−5.7543	−1.1510	3.0701
H	−5.1348	−4.1544	2.9112
H	−0.8418	−4.9691	5.2572
H	0.4464	−4.5304	4.1124
H	−1.2379	−6.5755	3.4256
H	−0.8662	−5.3332	2.2166
H	−3.4173	−5.6477	3.8222
H	−3.1931	−5.0397	2.1671
H	−1.3359	−2.6191	4.9067
H	1.6481	−0.4921	2.1524
H	1.3307	−2.0331	1.3652
H	7.2756	−1.6545	2.3859
H	6.1120	−1.6010	1.0454
H	5.9238	0.4887	1.3219
H	5.3027	−2.9965	2.9349
H	6.0916	0.7838	6.1314
H	2.2299	7.9845	−2.3931
H	−4.2865	4.9931	−4.5082
H	−2.6551	4.3823	−2.3268
H	0.0174	7.9939	−1.8647
H	5.8123	−0.7750	6.9276
H	4.4873	0.4220	6.8268
H	4.1527	−0.1963	4.5478
H	0.6292	−1.7331	4.1993
H	−7.2596	−3.0240	0.5357
H	−6.1365	−2.4882	−0.6980
H	3.8455	−0.7127	1.9146
H	−5.5780	−3.3388	4.4025
C	−10.7555	−5.1021	1.6624
C	−9.8578	−4.3240	0.7221
O	−8.7547	−3.9129	1.0945
C	−11.2732	−4.1537	2.7499
N	−10.3663	−4.0480	−0.4989
C	−9.6658	−3.2055	−1.4525
C	−8.9572	7.0087	3.1276
C	−8.6496	5.7965	2.2596
C	−8.2570	6.1500	0.8250
N	−7.7537	5.0973	0.1286
O	−8.4066	7.2729	0.3508
H	−10.4323	−3.6891	3.2698
H	−11.8818	−4.6912	3.4823
H	−11.8861	−3.3573	2.3165
H	−11.5873	−5.5721	1.1257
H	−8.7506	−3.6890	−1.8113
H	−7.8523	5.1902	2.6985
H	−9.5243	5.1368	2.2034
H	−7.5991	4.1836	0.5626
H	−7.4803	5.2552	−0.8289
H	−9.7340	7.6243	2.6677
H	−9.2923	6.6982	4.1221
H	−8.0729	7.6420	3.2447
H	−10.1545	−5.8985	2.1107
H	−9.3892	−2.2527	−0.9924
H	−10.3190	−3.0115	−2.3037
H	−11.2711	−4.4178	−0.7419

TS2

C	11.3265	5.2948	0.3330
C	10.6029	4.4420	−0.7255
C	9.4066	3.6796	−0.2222
C	8.1353	3.6263	−0.7559
N	9.4881	2.8323	0.8691
C	8.2894	2.2825	0.9858
N	7.4316	2.7319	0.0257
H	10.6712	6.0848	0.7149
H	11.3256	3.7251	−1.1401
H	10.2883	5.0758	−1.5640
H	7.6813	4.1270	−1.5975
H	7.9810	1.5436	1.7137
H	6.4797	2.3901	−0.1149
C	−9.4591	2.6366	−4.3057
C	−8.4017	3.2245	−3.3510
C	−7.3508	2.2246	−2.9142
C	−6.3070	1.8500	−3.7756
C	−7.3801	1.6393	−1.6442
C	−5.3498	0.9142	−3.3949
C	−6.4267	0.7025	−1.2416
C	−5.4090	0.3238	−2.1236
O	−4.4591	−0.6024	−1.8195
H	−9.9924	1.8085	−3.8275
H	−8.9040	3.6284	−2.4642
H	−7.9157	4.0755	−3.8456
H	−6.2385	2.3039	−4.7619
H	−8.1300	1.9361	−0.9176
H	−4.5424	0.6322	−4.0627
H	−6.4522	0.3363	−0.2215
H	−4.4874	−0.8936	−0.8728
C	−0.2276	−6.0276	−5.8938
C	−0.3053	−5.8029	−4.3786
C	−1.6428	−5.3170	−3.8490
C	−2.0076	−5.5831	−2.5228

C	−2.5437	−4.5744	−4.6254
C	−3.2132	−5.1351	−1.9886
C	−3.7547	−4.1182	−4.1076
C	−4.0994	−4.4021	−2.7843
O	−5.3190	−3.9811	−2.3366
H	−0.3461	−5.0914	−6.4485
H	−0.0500	−6.7367	−3.8639
H	0.4783	−5.0898	−4.0866
H	−1.3380	−6.1617	−1.8926
H	−2.3048	−4.3460	−5.6591
H	−3.4717	−5.3667	−0.9590
H	−4.4425	−3.5391	−4.7139
H	−5.3735	−4.0750	−1.3730
C	4.9972	−3.5812	−5.9093
C	5.2068	−4.5325	−4.7522
O	6.2625	−5.1310	−4.5582
C	5.3200	−2.1297	−5.5145
H	5.6541	−3.9034	−6.7210
H	6.3651	−2.0420	−5.2081
H	4.6959	−1.7907	−4.6811
H	5.1515	−1.4513	−6.3557
N	4.1415	−4.6388	−3.9140
C	4.2830	−5.3214	−2.6426
C	2.9669	−5.3648	−1.8756
C	2.3409	−3.9921	−1.5679
C	1.1043	−4.1884	−0.6888
C	3.3360	−3.0202	−0.9238
H	3.3764	−3.9900	−4.0210
H	5.0495	−4.8249	−2.0384
H	3.1572	−5.8792	−0.9252
H	2.2394	−5.9784	−2.4237
H	1.9934	−3.5414	−2.5112
H	1.3923	−4.5788	0.2947
H	0.5640	−3.2570	−0.5250
H	0.4011	−4.8945	−1.1419
H	4.1811	−2.7944	−1.5813
H	3.7428	−3.4402	0.0032
H	2.8650	−2.0682	−0.6709
C	9.1910	−5.2955	−1.2796
C	7.8313	−4.6048	−1.1172
C	7.8828	−3.1022	−1.2955
C	7.1951	−2.4908	−2.3498
C	8.6085	−2.2864	−0.4165
C	7.2125	−1.1041	−2.5082
C	8.6319	−0.9007	−0.5732
C	7.9244	−0.3017	−1.6177
H	9.9220	−4.9159	−0.5584
H	7.4254	−4.8379	−0.1235
H	7.1409	−5.0250	−1.8538
H	6.6654	−3.1175	−3.0576
H	9.1459	−2.7397	0.4128
H	6.6688	−0.6529	−3.3323
H	9.1838	−0.2795	0.1243
H	7.9289	0.7770	−1.7270
C	−2.4614	0.5636	0.6323
C	−1.3155	0.9973	0.0177
C	−1.3533	2.3272	−0.5266
N	−0.2739	2.9393	−0.9797
N	−2.5279	3.0186	−0.5820
C	−3.5989	2.5269	0.0124
N	−3.5844	1.3414	0.6604
S	1.9753	−0.2252	−3.3324
C	1.6274	0.4134	−1.7779
C	−4.8563	3.3343	−0.0119
N	0.4754	−0.1268	−1.3517
C	−0.1630	−1.0623	−2.2045
C	−1.4521	−1.7149	−1.8246
C	0.5431	−1.2334	−3.3542
C	0.2126	−2.0842	−4.5383
C	−0.0536	0.1678	0.0049
H	0.6994	2.6550	−0.7710
H	−0.3388	3.8944	−1.3270
H	−0.2499	−0.7832	0.5017
H	0.7628	0.6588	0.5309
H	−5.6914	2.8213	0.4619
H	−4.6812	4.2934	0.4848
H	−5.1198	3.5419	−1.0527
H	−2.5486	−0.4038	1.1149
H	−1.4415	−2.0807	−0.7954
H	−1.6358	−2.5729	−2.4720
H	−2.2974	−1.0319	−1.9394
H	−0.6139	−2.7585	−4.3113
H	1.0704	−2.6917	−4.8484
C	1.6194	−3.7582	7.0450
C	0.9001	−2.4620	7.4362
C	1.4845	−1.2283	6.7732
O	1.4664	−1.3195	5.4408
O	1.8976	−0.2576	7.3824
C	−8.4847	2.1303	4.2038
C	−6.9864	1.9783	3.9096
C	−6.6443	1.5901	2.4582
O	−7.3784	2.0374	1.5376
O	−5.5946	0.8897	2.2947
H	−8.6662	2.7386	5.0953
H	−8.9865	2.5861	3.3517
H	−6.5061	1.2671	4.5868
H	−6.4784	2.9402	4.0729
C	−1.0153	1.2083	5.6612
C	−0.8802	0.7663	4.2032
C	0.3779	1.2681	3.5731
C	0.5425	2.1718	2.5549
N	1.6338	0.8217	3.9692
C	2.5174	1.4235	3.1815
N	1.9002	2.2655	2.3218
H	−0.1456	0.9107	6.2514
H	−0.9058	−0.3268	4.1435
H	−1.7371	1.1234	3.6214
H	−0.1704	2.7481	1.9891
H	3.5823	1.2495	3.1765
H	2.2815	2.6197	1.4300
C	5.2830	6.6374	3.9575
C	5.3938	5.2222	4.5333
C	5.7688	4.1198	3.5237
C	5.8163	2.7606	4.2366
C	4.8035	4.0882	2.3315
H	4.4728	6.7126	3.2258
H	4.4354	4.9492	4.9981
H	6.1339	5.2200	5.3444
H	6.7755	4.3332	3.1358
H	4.8435	2.5292	4.6880
H	6.0733	1.9432	3.5563
H	6.5548	2.7664	5.0463
H	3.7660	4.0530	2.6807
H	4.9781	3.2125	1.7006
H	4.9096	4.9739	1.6976
H	−0.0799	−1.4691	−5.3969
H	−8.9464	1.1500	4.3682
H	11.6185	4.6680	1.1796
H	12.2238	5.7623	−0.0864
H	−10.1928	3.3952	−4.5977
H	−8.9896	2.2471	−5.2150
H	−1.0966	2.2985	5.7282
H	−1.9098	0.7720	6.1172
H	6.2110	6.9334	3.4540
H	5.0842	7.3698	4.7474
H	−0.1547	−2.5172	7.1367
H	0.9327	−2.2996	8.5158
H	1.6559	−3.8859	5.9596
H	1.1189	−4.6263	7.4847
H	2.6544	−3.7488	7.4001
H	9.1004	−6.3765	−1.1307
H	9.5910	−5.1222	−2.2834
H	4.6500	−6.3335	−2.8377
H	0.7452	−6.4471	−6.1661
H	−1.0068	−6.7177	−6.2324
H	3.9627	−3.6485	−6.2691
H	1.6677	−0.4161	4.9743
H	−4.4543	1.0577	1.2378
C	2.1058	7.1676	−1.9095
C	0.7796	6.5021	−1.6450
O	0.5585	5.3497	−2.0264
N	−0.1588	7.2119	−0.9687
C	−1.5186	6.7001	−0.9181
C	−2.0710	6.5002	−2.3423
O	−1.8391	7.3329	−3.2157
N	−2.8160	5.3846	−2.5018
C	−3.3057	4.9754	−3.8104
C	−2.7153	3.6281	−4.2502
C	−1.1820	3.5853	−4.3412
C	−0.6960	2.1561	−4.6031
C	−0.6246	4.5680	−5.3767
C	3.5708	2.2355	−2.3108
C	3.0131	3.3142	−3.0085
C	3.5088	3.6643	−4.2622
C	4.5714	2.9515	−4.8245
C	5.1410	1.8885	−4.1223
C	4.6417	1.5273	−2.8706
C	2.9703	1.8418	−0.9861
O	2.3009	2.7256	−0.3465
C	3.7731	0.8907	−0.0717
O	4.9930	1.1961	0.0766
O	3.1435	−0.0047	0.5357
H	2.9031	6.4746	−1.6320
H	2.1878	7.3572	−2.9838
H	−1.5464	5.7703	−0.3434
H	−2.1389	7.4341	−0.3978
H	−3.0563	2.8541	−3.5509
H	−3.1526	3.3693	−5.2253
H	−0.7808	3.8920	−3.3739
H	0.4660	4.4829	−5.4293
H	−1.0255	4.3615	−6.3778
H	−0.8628	5.6024	−5.1137
H	−1.0585	1.7825	−5.5699
H	−1.0470	1.4676	−3.8265
H	0.3970	2.1126	−4.6133
H	−3.0525	5.7768	−4.5058
H	2.1947	3.8706	−2.5659
H	3.0652	4.4978	−4.7999
H	4.9583	3.2283	−5.8012
H	5.9792	1.3420	−4.5452
H	5.0929	0.7123	−2.3184
C	−5.1479	−3.6895	2.9945
C	−3.7108	−3.3023	3.3397
O	−3.4588	−2.2537	3.9282
C	−5.9290	−2.5045	2.3876
C	−5.5344	−2.1915	0.9572
N	−6.3975	−2.5797	−0.0143
O	−4.4684	−1.6229	0.6749
N	−2.7394	−4.2130	3.0583
C	−1.3702	−3.9374	3.4540
C	−0.6794	−2.9543	2.5039
O	−1.0663	−2.7860	1.3409
C	−0.6894	−5.3134	3.3091
C	−1.3858	−5.8888	2.0646
C	−2.8389	−5.3886	2.1843
N	0.4410	−2.4262	3.0173
C	1.5410	−1.9436	2.2163
C	2.8411	−2.5878	2.7134
O	2.8514	−3.6864	3.2722
N	3.9263	−1.8191	2.4844
C	5.2814	−2.2389	2.7850
C	5.7083	−2.0084	4.2425
O	6.7687	−2.4767	4.6521
C	6.2577	−1.4671	1.8825
O	6.1644	−0.0729	2.1155
N	4.8920	−1.2339	4.9897
C	5.2678	−0.8039	6.3206
H	−7.0000	−2.7193	2.4289
H	−5.7168	−1.5991	2.9635
H	−5.2063	−4.5637	2.3420
H	−0.9089	−5.9197	4.1942
H	0.3947	−5.2225	3.2092
H	−1.3295	−6.9787	2.0060
H	−0.9311	−5.4708	1.1638
H	−3.4920	−6.1497	2.6296
H	−3.2462	−5.1094	1.2078
H	−1.3460	−3.5472	4.4744
H	1.6213	−0.8611	2.2411
H	1.3712	−2.2382	1.1815
H	7.2671	−1.8120	2.1222
H	6.0513	−1.7063	0.8328
H	5.7293	0.3534	1.3376
H	5.3871	−3.3137	2.6036
H	6.0103	0.0029	6.2849
H	2.2335	8.1088	−1.3696
H	−4.4001	4.8930	−3.7804
H	−2.7870	4.6699	−1.7732
H	−0.0307	8.2097	−0.8992
H	5.7105	−1.6442	6.8606
H	4.3789	−0.4514	6.8464
H	4.0476	−0.8843	4.5658
H	0.6517	−2.5522	4.0006
H	−7.3152	−2.9701	0.1986

H	-6.2195	-2.2442	-0.9531
H	3.7993	-1.0111	1.8614
H	-5.6257	-3.9638	3.9418
C	-10.6672	-5.4075	1.0202
C	-9.8178	-4.4749	0.1807
O	-8.7689	-3.9998	0.6253
C	-11.2986	-4.6061	2.1642
N	-10.3139	-4.1419	-1.0317
C	-9.6676	-3.1597	-1.8842
C	-9.0633	6.4480	3.8722
C	-8.7712	5.2862	2.9316
C	-8.4475	5.7124	1.4993
N	-8.0126	4.6939	0.7112
O	-8.5908	6.8659	1.1015
H	-10.5182	-4.1145	2.7495
H	-11.8745	-5.2568	2.8281
H	-11.9704	-3.8335	1.7776
H	-11.4378	-5.9006	0.4169
H	-8.7099	-3.5308	-2.2656
H	-7.9424	4.6787	3.3075
H	-9.6335	4.6108	2.8855
H	-7.8432	3.7474	1.0647
H	-7.7773	4.9140	-0.2445
H	-9.8798	7.0627	3.4853
H	-9.3368	6.0804	4.8661
H	-8.1909	7.1000	3.9733
H	-10.0092	-6.1836	1.4217
H	-9.4779	-2.2373	-1.3283
H	-10.3198	-2.9367	-2.7291
H	-11.1729	-4.5695	-1.3381

Int2

C	11.3333	5.1690	0.1150
C	10.5309	4.3294	-0.9006
C	9.3374	3.5975	-0.3427
C	8.0415	3.5741	-0.8191
N	9.4472	2.7484	0.7453
C	8.2420	2.2294	0.9198
N	7.3535	2.6963	-0.0037
H	10.7208	5.9787	0.5253
H	11.2136	3.5922	-1.3464
H	10.1941	4.9688	-1.7262
H	7.5586	4.0792	-1.6416
H	7.9461	1.5051	1.6672
H	6.3797	2.4050	-0.0807
C	-9.5063	2.5546	-4.3007
C	-8.4469	3.1005	-3.3230
C	-7.3900	2.0932	-2.9120
C	-6.3766	1.7006	-3.8012
C	-7.3652	1.5413	-1.6267
C	-5.3929	0.7886	-3.4286
C	-6.3828	0.6309	-1.2313
C	-5.3883	0.2432	-2.1359
O	-4.4007	-0.6458	-1.8332
H	-10.0463	1.7126	-3.8552
H	-8.9494	3.4746	-2.4234
H	-7.9601	3.9690	-3.7859
H	-6.3480	2.1259	-4.8021
H	-8.0898	1.8485	-0.8791
H	-4.6072	0.4992	-4.1192
H	-6.3687	0.2981	-0.1994
H	-4.4086	-0.9149	-0.8800
C	-0.3563	-6.2253	-5.7150
C	-0.2989	-5.5183	-4.3530
C	-1.6512	-5.0997	-3.8155
C	-2.1703	-5.6786	-2.6542
C	-2.4344	-4.1253	-4.4569
C	-3.4163	-5.3094	-2.1486
C	-3.6687	-3.7249	-3.9515
C	-4.1662	-4.3213	-2.7886
O	-5.3988	-3.9335	-2.3396
H	-0.8026	-5.5795	-6.4781
H	0.1811	-6.1785	-3.6229
H	0.3541	-4.6406	-4.4366
H	-1.5943	-6.4391	-2.1349
H	-2.0687	-3.6599	-5.3684
H	-3.8076	-5.7931	-1.2591
H	-4.2505	-2.9447	-4.4291
H	-5.5132	-4.2156	-1.4197
C	4.8876	-3.8232	-5.8351
C	5.1283	-4.7130	-4.6365
O	6.2244	-5.2036	-4.3758
C	5.1995	-2.3548	-5.4970
H	5.5407	-4.1672	-6.6411
H	6.2449	-2.2475	-5.1975
H	4.5772	-1.9951	-4.6703
H	5.0220	-1.7083	-6.3611
N	4.0474	-4.8695	-3.8304
C	4.1835	-5.4670	-2.5168
C	2.8687	-5.4097	-1.7496
C	2.3063	-3.9937	-1.5344
C	1.0367	-4.0809	-0.6872
C	3.3372	-3.0493	-0.9112
H	3.2087	-4.3480	-4.0323
H	4.9693	-4.9496	-1.9596
H	3.0367	-5.8683	-0.7674
H	2.1177	-6.0289	-2.2590
H	2.0097	-3.5740	-2.5096
H	1.2659	-4.4892	0.3034
H	0.5787	-3.1033	-0.5371
H	0.2913	-4.7285	-1.1601
H	4.2005	-2.8868	-1.5635
H	3.7136	-3.4535	0.0356
H	2.9065	-2.0717	-0.6909
C	9.1014	-5.4438	-1.1899
C	7.7621	-4.7046	-1.0687
C	7.8542	-3.2034	-1.2566
C	7.1565	-2.5791	-2.2976
C	8.6142	-2.3995	-0.3953
C	7.1971	-1.1930	-2.4614
C	8.6639	-1.0148	-0.5588
C	7.9468	-0.4031	-1.5896
H	9.8273	-5.0853	-0.4529
H	7.3250	-4.9164	-0.0833
H	7.0781	-5.1101	-1.8189
H	6.5988	-3.1968	-2.9915
H	9.1584	-2.8598	0.4255
H	6.6418	-0.7330	-3.2740
H	9.2427	-0.4015	0.1241
H	7.9746	0.6758	-1.7006
C	-2.3536	0.6400	0.7190
C	-1.2310	1.1085	0.0920
C	-1.3304	2.4050	-0.5322
N	-0.2765	3.0124	-1.0271
N	-2.5448	3.0316	-0.6170
C	-3.5844	2.5249	0.0135
N	-3.5146	1.3673	0.7103
S	2.1442	0.0016	-3.2517
C	1.7976	0.6085	-1.6860
C	-4.8719	3.2838	-0.0214
N	0.6470	0.0916	-1.2304
C	0.0092	-0.8190	-2.0977
C	-1.2871	-1.4724	-1.7402
C	0.7092	-0.9825	-3.2544
C	0.3431	-1.7792	-4.4628
C	0.0827	0.3642	0.1271
H	0.7684	2.7690	-0.7552
H	-0.3955	3.9206	-1.4701
H	-0.0483	-0.5961	0.6267
H	0.8452	0.9270	0.6509
H	-5.6919	2.7456	0.4500
H	-4.7344	4.2488	0.4762
H	-5.1344	3.4824	-1.0638
H	-2.3923	-0.3152	1.2320
H	-1.3163	-1.7958	-0.6975
H	-1.4322	-2.3564	-2.3627
H	-2.1392	-0.8097	-1.9140
H	-0.4367	-2.4987	-4.2180
H	1.2052	-2.3236	-4.8619
C	1.6037	-3.6162	7.1428
C	0.7839	-2.3408	7.3669
C	1.4156	-1.1356	6.6981
O	1.4634	-1.2761	5.3742
O	1.8207	-0.1541	7.3002
C	-8.4737	2.2745	4.2123
C	-6.9759	2.0949	3.9218
C	-6.6338	1.6215	2.4936
O	-7.3790	2.0057	1.5520
O	-5.5749	0.9298	2.3637
H	-8.6481	2.9456	5.0588
H	-8.9777	2.6697	3.3316
H	-6.4992	1.4213	4.6392
H	-6.4602	3.0605	4.0300
C	-1.0014	1.3318	5.6414
C	-0.8156	0.8489	4.1999
C	0.4380	1.3663	3.5689
C	0.5978	2.3276	2.6042
N	1.6926	0.8576	3.8893
C	2.5657	1.4774	3.1064
N	1.9504	2.3952	2.3247
H	-0.1388	1.0732	6.2601
H	-0.8008	-0.2468	4.1788
H	-1.6721	1.1599	3.5909
H	-0.1171	2.9459	2.0859
H	3.6233	1.2707	3.0521
H	2.2930	2.7166	1.3982
C	5.3274	6.6602	3.7440
C	5.4490	5.2979	4.4306
C	5.8200	4.1322	3.4955
C	5.8100	2.8112	4.2772
C	4.8836	4.0731	2.2826
H	4.5074	6.6733	3.0202
H	4.4931	5.0568	4.9176
H	6.1928	5.3596	5.2361
H	6.8423	4.2982	3.1249
H	4.8114	2.6125	4.6855
H	6.0921	1.9596	3.6513
H	6.5047	2.8503	5.1242
H	3.8386	4.1275	2.6050
H	5.0097	3.1468	1.7197
H	5.0618	4.9027	1.5915
H	-0.0370	-1.1283	-5.2584
H	-8.9383	1.3104	4.4483
H	11.6535	4.5393	0.9492
H	12.2186	5.6087	-0.3562
H	-10.2337	3.3293	-4.5640
H	-9.0394	2.1971	-5.2245
H	-1.1144	2.4208	5.6702
H	-1.8953	0.8843	6.0878
H	6.2477	6.9153	3.2048
H	5.1382	7.4557	4.4733
H	-0.2182	-2.4639	6.9386
H	0.6796	-2.1163	8.4309
H	1.7938	-3.7759	6.0781
H	1.0844	-4.4912	7.5466
H	2.5774	-3.5441	7.6376
H	8.9666	-6.5191	-1.0340
H	9.5332	-5.2952	-2.1845
H	4.5162	-6.5045	-2.6307
H	0.6469	-6.5035	-6.0531
H	-0.9630	-7.1343	-5.6561
H	3.8510	-3.9182	-6.1824
H	1.7061	-0.3948	4.9110
H	-4.3647	1.0713	1.2841
C	2.1117	7.0548	-2.1126
C	0.7676	6.4146	-1.8888
O	0.5098	5.3012	-2.3540
N	-0.1390	7.0999	-1.1487
C	-1.5075	6.6150	-1.0723
C	-2.0852	6.4022	-2.4844
O	-1.8848	7.2321	-3.3678
N	-2.8116	5.2722	-2.6221
C	-3.3310	4.8556	-3.9139
C	-2.8333	3.4647	-4.3267
C	-1.3082	3.3063	-4.4257
C	-0.9539	1.8618	-4.7972
C	-0.6672	4.3076	-5.3918
C	3.6538	2.2078	-2.1350
C	3.2106	3.3690	-2.7770
C	3.9631	3.9328	-3.8058
C	5.1724	3.3513	-4.1963
C	5.6190	2.1943	-3.5573
C	4.8599	1.6206	-2.5352
C	2.7773	1.6122	-1.0180
O	2.1003	2.5831	-0.3253
C	3.6282	0.7948	0.0236
O	4.7540	1.2643	0.3350
O	3.0562	-0.1979	0.5392
H	2.8885	6.3392	-1.8325
H	2.2213	7.2630	-3.1808
H	-1.5504	5.6960	-0.4807
H	-2.1059	7.3713	-0.5586

H	-3.2251	2.7252	-3.6170
H	-3.2911	3.2241	-5.2973
H	-0.8825	3.5000	-3.4396
H	0.4136	4.1411	-5.4486
H	-1.0805	4.2028	-6.4039
H	-0.8217	5.3368	-5.0586
H	-1.3494	1.5990	-5.7871
H	-1.3681	1.1521	-4.0720
H	0.1318	1.7220	-4.8208
H	-3.0360	5.6213	-4.6329
H	2.2825	3.8320	-2.4627
H	3.6033	4.8310	-4.3002
H	5.7639	3.7988	-4.9901
H	6.5646	1.7416	-3.8414
H	5.2266	0.7366	-2.0287
C	-5.1925	-3.6036	3.1402
C	-3.6926	-3.5000	3.3904
O	-3.2477	-2.8135	4.3059
C	-5.7955	-2.3203	2.5166
C	-5.4348	-2.1183	1.0577
N	-6.3094	-2.5951	0.1351
O	-4.3763	-1.5713	0.7111
N	-2.8826	-4.2680	2.6099
C	-1.4639	-4.3570	2.9000
C	-0.6692	-3.1588	2.3552
O	-1.1222	-2.3926	1.4933
C	-1.0660	-5.6515	2.1656
C	-1.9220	-5.5794	0.8941
C	-3.2480	-4.9576	1.3692
N	0.5731	-3.0910	2.8422
C	1.5797	-2.1760	2.3706
C	2.9328	-2.6903	2.8638
O	3.0198	-3.7334	3.5192
N	3.9649	-1.9002	2.5180
C	5.3455	-2.2300	2.8227
C	5.7483	-1.9763	4.2807
O	6.8049	-2.4321	4.7135
C	6.2771	-1.3927	1.9315
O	6.1455	-0.0116	2.2098
N	4.9171	-1.1875	4.9925
C	5.2701	-0.7130	6.3113
H	-6.8845	-2.3688	2.6081
H	-5.4407	-1.4347	3.0488
H	-5.4624	-4.4751	2.5382
H	-1.3473	-6.5146	2.7780
H	0.0048	-5.7101	1.9616
H	-2.0653	-6.5543	0.4214
H	-1.4540	-4.9155	0.1674
H	-4.0048	-5.7294	1.5613
H	-3.6442	-4.2503	0.6360
H	-1.3165	-4.4104	3.9814
H	1.4142	-1.1630	2.7407
H	1.5789	-2.1250	1.2809
H	7.3014	-1.7059	2.1492
H	6.0698	-1.6098	0.8765
H	5.6095	0.4164	1.4989
H	5.5253	-3.2945	2.6377
H	5.9835	0.1192	6.2605
H	2.2528	7.9819	-1.5517
H	-4.4290	4.8401	-3.8818
H	-2.7902	4.5752	-1.8746
H	0.0317	8.0778	-0.9749
H	5.7383	-1.5252	6.8719
H	4.3665	-0.3798	6.8256
H	4.0892	-0.8351	4.5397
H	0.9279	-3.7648	3.5145
H	-7.2473	-2.9010	0.3922
H	-6.1459	-2.3200	-0.8260
H	3.7666	-1.1418	1.8495
H	-5.6408	-3.7293	4.1292
C	-10.5934	-5.3024	1.3235
C	-9.8076	-4.3558	0.4361
O	-8.8071	-3.7710	0.8594
C	-11.3563	-4.4921	2.3793
N	-10.3147	-4.1406	-0.8001
C	-9.7435	-3.1711	-1.7190
C	-9.0201	6.5860	3.7660
C	-8.7335	5.3737	2.8897
C	-8.4244	5.7188	1.4326
N	-8.0030	4.6587	0.6935
O	-8.5651	6.8507	0.9757
H	-10.6590	-3.8768	2.9526
H	-11.8876	-5.1523	3.0702
H	-12.0887	-3.8281	1.9099
H	-11.2821	-5.9214	0.7370
H	-8.8262	-3.5459	-2.1880
H	-7.8995	4.7909	3.2922
H	-9.5942	4.6951	2.8907
H	-7.8425	3.7242	1.0831
H	-7.7816	4.8297	-0.2755
H	-9.8432	7.1749	3.3535
H	-9.2819	6.2745	4.7819
H	-8.1495	7.2459	3.8207
H	-9.8753	-5.9697	1.8084
H	-9.4984	-2.2517	-1.1820
H	-10.4710	-2.9447	-2.4994
H	-11.1211	-4.6707	-1.0893

Int3a

C	11.3150	5.1902	0.2912
C	10.4638	4.4415	-0.7542
C	9.3327	3.6189	-0.1947
C	8.0088	3.5978	-0.5844
N	9.5399	2.6768	0.7984
C	8.3647	2.1042	1.0035
N	7.4036	2.6239	0.1871
H	10.7132	5.9300	0.8291
H	11.1302	3.7783	-1.3241
H	10.0530	5.1552	-1.4793
H	7.4569	4.1630	-1.3197
H	8.1379	1.3035	1.6946
H	6.4345	2.3179	0.1488
C	-9.4969	2.6242	-4.2811
C	-8.4581	3.1756	-3.2838
C	-7.3862	2.1816	-2.8808
C	-6.3116	1.8852	-3.7352
C	-7.4131	1.5401	-1.6380
C	-5.3202	0.9774	-3.3722
C	-6.4254	0.6314	-1.2532
C	-5.3709	0.3367	-2.1247
O	-4.3743	-0.5421	-1.8273
H	-10.0206	1.7616	-3.8563
H	-8.9783	3.5233	-2.3835
H	-7.9846	4.0614	-3.7264
H	-6.2441	2.3816	-4.7009
H	-8.1875	1.7760	-0.9144
H	-4.4884	0.7621	-4.0351
H	-6.4539	0.2274	-0.2480
H	-4.4374	-0.8919	-0.9016
C	-0.3227	-6.1094	-5.8203
C	-0.2435	-5.4631	-4.4296
C	-1.5864	-5.0499	-3.8657
C	-2.1039	-5.6658	-2.7226
C	-2.3640	-4.0468	-4.4678
C	-3.3443	-5.3052	-2.1981
C	-3.5922	-3.6540	-3.9419
C	-4.0887	-4.2889	-2.7995
O	-5.3158	-3.9061	-2.3313
H	-0.7653	-5.4247	-6.5509
H	0.2322	-6.1610	-3.7328
H	0.4226	-4.5925	-4.4802
H	-1.5317	-6.4485	-2.2331
H	-1.9998	-3.5543	-5.3655
H	-3.7350	-5.8176	-1.3247
H	-4.1701	-2.8525	-4.3879
H	-5.4419	-4.2440	-1.4323
C	4.9171	-3.6957	-5.8666
C	5.1465	-4.6221	-4.6935
O	6.2297	-5.1526	-4.4605
C	5.2027	-2.2327	-5.4862
H	5.5867	-4.0093	-6.6711
H	6.2433	-2.1149	-5.1750
H	4.5686	-1.9046	-4.6552
H	5.0214	-1.5667	-6.3347
N	4.0685	-4.7695	-3.8810
C	4.1994	-5.4054	-2.5847
C	2.8796	-5.3874	-1.8261
C	2.2937	-3.9860	-1.5818
C	1.0305	-4.1155	-0.7304
C	3.3134	-3.0363	-0.9478
H	3.2472	-4.2119	-4.0561
H	4.9760	-4.8993	-2.0041
H	3.0493	-5.8647	-0.8533
H	2.1413	-6.0058	-2.3540
H	1.9839	-3.5557	-2.5490
H	1.2756	-4.5296	0.2538
H	0.5458	-3.1533	-0.5649
H	0.3023	-4.7779	-1.2098
H	4.1658	-2.8430	-1.6064
H	3.7077	-3.4531	-0.0147
H	2.8672	-2.0731	-0.6981
C	9.1105	-5.3991	-1.2325
C	7.7629	-4.6691	-1.1951
C	7.8583	-3.1641	-1.3419
C	7.1302	-2.5079	-2.3407
C	8.6443	-2.3871	-0.4796
C	7.1697	-1.1177	-2.4652
C	8.6900	-0.9989	-0.6008
C	7.9472	-0.3558	-1.5934
H	9.7750	-5.0671	-0.4285
H	7.2557	-4.9034	-0.2485
H	7.1355	-5.0608	-2.0004
H	6.5489	-3.1036	-3.0338
H	9.2121	-2.8704	0.3105
H	6.5939	-0.6339	-3.2493
H	9.2895	-0.4087	0.0834
H	7.9771	0.7259	-1.6726
C	-2.4305	0.6129	0.7703
C	-1.3127	1.0978	0.1379
C	-1.4510	2.3790	-0.4951
N	-0.4209	3.0015	-1.0458
N	-2.6580	3.0015	-0.5604
C	-3.6870	2.4837	0.0843
N	-3.5970	1.3232	0.7695
S	2.1535	0.0930	-3.1558
C	1.7740	0.6680	-1.5877
C	-4.9691	3.2487	0.0902
N	0.6184	0.1354	-1.1592
C	0.0054	-0.7634	-2.0552
C	-1.2867	-1.4410	-1.7324
C	0.7294	-0.8992	-3.2019
C	0.4006	-1.6899	-4.4254
C	0.0133	0.3698	0.1872
H	0.5502	2.7932	-0.7581
H	-0.5369	3.9301	-1.4416
H	-0.1206	-0.6060	0.6590
H	0.7473	0.9145	0.7668
H	-5.8054	2.6649	0.4689
H	-4.8485	4.1444	0.7088
H	-5.1884	3.5753	-0.9290
H	-2.4518	-0.3443	1.2821
H	-1.3182	-1.8032	-0.7023
H	-1.4152	-2.3029	-2.3881
H	-2.1440	-0.7813	-1.8918
H	-0.3287	-2.4632	-4.1890
H	1.2915	-2.1689	-4.8435
C	1.5671	-3.7489	7.0960
C	0.6993	-2.5066	6.8482
C	1.5319	-1.3187	6.3414
O	1.9221	-1.4102	5.1081
O	1.7745	-0.3694	7.1012
C	-8.5068	2.1794	4.2300
C	-7.0080	2.0130	3.9479
C	-6.6604	1.5261	2.5289
O	-7.4159	1.8662	1.5807
O	-5.5781	0.8646	2.4129
H	-8.6920	2.8563	5.0693
H	-9.0143	2.5592	3.3445
H	-6.5258	1.3529	4.6738
H	-6.5001	2.9838	4.0454
C	-1.0400	1.2227	5.6784
C	-0.8467	0.7388	4.2372
C	0.3765	1.3051	3.5932
C	0.5408	2.3010	2.6600
N	1.6498	0.8351	3.8571
C	2.5124	1.5060	3.0709
N	1.8811	2.4120	2.3210
H	-0.1903	0.9321	6.3029
H	-0.7905	-0.3559	4.2200
H	-1.7161	1.0164	3.6300
H	-0.2117	2.9250	2.1997
H	3.5698	1.2970	3.0352

H	2.1644	2.6102	0.8332
C	5.2879	6.5989	3.9180
C	5.4689	5.1637	4.4145
C	5.7894	4.1327	3.3174
C	5.8815	2.7331	3.9399
C	4.7587	4.1850	2.1827
H	4.4159	6.6910	3.2639
H	4.5515	4.8448	4.9293
H	6.2658	5.1362	5.1693
H	6.7738	4.3784	2.8916
H	4.9416	2.4690	4.4379
H	6.0978	1.9570	3.2017
H	6.6705	2.6922	4.6993
H	3.7389	4.1089	2.5724
H	4.8928	3.3587	1.4830
H	4.8337	5.1182	1.6141
H	−0.0285	−1.0475	−5.2027
H	−8.9610	1.2124	4.4742
H	11.7076	4.4835	1.0269
H	12.1543	5.7067	−0.1864
H	−10.2399	3.3869	−4.5357
H	−9.0131	2.2954	−5.2069
H	−1.1326	2.3135	5.7081
H	−1.9481	0.7884	6.1088
H	6.1642	6.9350	3.3501
H	5.1483	7.2931	4.7542
H	−0.0671	−2.7343	6.0964
H	0.1957	−2.2023	7.7702
H	2.0953	−4.0306	6.1791
H	0.9647	−4.6014	7.4302
H	2.3203	−3.5446	7.8643
H	8.9686	−6.4792	−1.1234
H	9.6206	−5.2176	−2.1837
H	4.5442	−6.4344	−2.7310
H	0.6736	−6.3873	−6.1784
H	−0.9425	−7.0109	−5.7950
H	3.8872	−3.7940	−6.2331
H	1.8719	−0.0182	4.4834
H	−4.4540	1.0146	1.3586
C	2.1011	7.1022	−1.9461
C	0.7647	6.4626	−1.6890
O	0.5296	5.3155	−2.0803
N	−0.1639	7.1824	−1.0141
C	−1.5309	6.6927	−0.9562
C	−2.0764	6.4634	−2.3787
O	−1.8215	7.2639	−3.2747
N	−2.8391	5.3563	−2.5160
C	−3.3281	4.9287	−3.8178
C	−2.6793	3.6158	−4.2797
C	−1.1557	3.6704	−4.4696
C	−0.5925	2.2638	−4.7008
C	−0.7407	4.6347	−5.5863
C	3.6544	2.2488	−1.9837
C	3.2116	3.4138	−2.6220
C	3.9773	3.9896	−3.6343
C	5.1918	3.4145	−4.0152
C	5.6332	2.2524	−3.3828
C	4.8662	1.6670	−2.3742
C	2.7728	1.6215	−0.8997
O	2.0884	2.6427	−0.2169
C	3.5985	0.7686	0.1432
O	4.7311	1.2056	0.4486
O	2.9946	−0.2220	0.6084
H	2.8868	6.4043	−1.6479
H	2.1975	7.2743	−3.0222
H	−1.5774	5.7792	−0.3565
H	−2.1429	7.4493	−0.4594
H	−2.9283	2.8353	−3.5496
H	−3.1581	3.3138	−5.2225
H	−0.7116	4.0503	−3.5476
H	0.3472	4.6255	−5.7138
H	−1.1930	4.3520	−6.5460
H	−1.0349	5.6610	−5.3507
H	−0.9935	1.8192	−5.6214
H	−0.8438	1.5982	−3.8678
H	0.4987	2.2917	−4.7881
H	−3.1324	5.7461	−4.5125
H	2.2781	3.8767	−2.3238
H	3.6216	4.8920	−4.1233
H	5.7919	3.8709	−4.7969
H	6.5824	1.8043	−3.6595
H	5.2304	0.7797	−1.8735
C	−5.2087	−3.6704	3.0597
C	−3.7124	−3.5276	3.3136
O	−3.2886	−2.8024	4.2082
C	−5.8396	−2.3881	2.4651
C	−5.4922	−2.1563	1.0075
N	−6.3740	−2.6135	0.0865
O	−4.4384	−1.5969	0.6614
N	−2.8847	−4.3048	2.5625
C	−1.4693	−4.3748	2.8739
C	−0.6719	−3.1836	2.3196
O	−1.1188	−2.4299	1.4391
C	−1.0493	−5.6779	2.1664
C	−1.8877	−5.6323	0.8815
C	−3.2280	−5.0247	1.3338
N	0.5637	−3.1143	2.8144
C	1.5972	−2.2492	2.3081
C	2.9385	−2.8335	2.7575
O	3.0035	−3.9520	3.2764
N	3.9842	−2.0241	2.5183
C	5.3558	−2.3558	2.8520
C	5.7867	−1.9364	4.2682
O	6.9473	−2.1412	4.6260
C	6.3246	−1.6897	1.8626
O	6.3573	−0.2826	2.0144
N	4.8669	−1.3075	5.0253
C	5.2319	−0.8232	6.3402
H	−6.9273	−2.4536	2.5633
H	−5.4922	−1.5090	3.0128
H	−5.4568	−4.5353	2.4396
H	−1.3335	−6.5336	2.7878
H	0.0253	−5.7293	1.9825
H	−2.0115	−6.6134	0.4155
H	−1.4196	−4.9677	0.1555
H	−3.9729	−5.8063	1.5335
H	−3.6310	−4.3379	0.5839
H	−1.3361	−4.4105	3.9580
H	1.4875	−1.2343	2.6820
H	1.5574	−2.2087	1.2192
H	7.3224	−2.0694	2.0875
H	6.0643	−1.9791	0.8360
H	5.7059	0.1458	1.4203
H	5.4716	−3.4433	2.7925
H	6.0249	−0.0725	6.2611
H	2.2391	8.0502	−1.4206
H	−4.4148	4.7863	−3.7635
H	−2.8631	4.6679	−1.7622
H	−0.0099	8.1730	−0.9091
H	5.6065	−1.6332	6.9777
H	4.3435	−0.3817	6.7959
H	3.8722	−1.3480	4.8083
H	0.9018	−3.7778	3.5047
H	−7.2909	−2.9821	0.3352
H	−6.2030	−2.3604	−0.8787
H	3.7962	−1.1843	1.9678
H	−5.6542	−3.8291	4.0456
C	−10.7137	−5.3684	1.1254
C	−9.8639	−4.4486	0.2699
O	−8.7996	−3.9885	0.6919
C	−11.3668	−4.5554	2.2502
N	−10.3782	−4.1149	−0.9365
C	−9.7359	−3.1513	−1.8132
C	−9.0594	6.4978	3.8653
C	−8.7721	5.2818	2.9945
C	−8.4804	5.6163	1.5317
N	−8.0942	4.5463	0.7856
O	−8.6035	6.7492	1.0731
H	−10.5990	−4.0448	2.8362
H	−11.9407	−5.2032	2.9183
H	−12.0443	−3.7977	1.8444
H	−11.4738	−5.8816	0.5253
H	−8.8066	−3.5500	−2.2352
H	−7.9275	4.7112	3.3927
H	−9.6256	4.5950	3.0116
H	−7.9330	3.6099	1.1686
H	−7.8774	4.7164	−0.1847
H	−9.8986	7.0718	3.4646
H	−9.2978	6.1909	4.8883
H	−8.1974	7.1701	3.8980
H	−10.0514	−6.1295	1.5474
H	−9.4934	−2.2383	−1.2625
H	−10.4149	−2.9037	−2.6297
H	−11.2490	−4.5328	−1.2226

TS4a

C	11.4117	5.1273	−0.1450
C	10.6107	4.2384	−1.1134
C	9.4951	3.4648	−0.4675
C	8.1606	3.4282	−0.8121
N	9.7257	2.6139	0.6013
C	8.5522	2.0813	0.8924
N	7.5708	2.5377	0.0635
H	10.7726	5.9009	0.2935
H	11.3035	3.5297	−1.5880
H	10.1928	4.8488	−1.9231
H	7.5877	3.9307	−1.5748
H	8.3495	1.3673	1.6780
H	6.6033	2.2363	0.0663
C	−9.5050	2.6987	−4.2943
C	−8.3912	3.1883	−3.3481
C	−7.3813	2.1248	−2.9611
C	−6.4017	1.6896	−3.8674
C	−7.3662	1.5612	−1.6802
C	−5.4552	0.7310	−3.5140
C	−6.4218	0.6039	−1.3049
C	−5.4556	0.1779	−2.2248
O	−4.4994	−0.7501	−1.9352
H	−10.0795	1.8909	−3.8292
H	−8.8478	3.5927	−2.4371
H	−7.8695	4.0267	−3.8282
H	−6.3664	2.1205	−4.8656
H	−8.0627	1.8995	−0.9188
H	−4.6922	0.4133	−4.2176
H	−6.4140	0.2528	−0.2799
H	−4.4966	−0.9911	−0.9698
C	−0.4952	−6.2473	−5.5589
C	−0.6246	−6.0139	−4.0415
C	−2.0072	−5.6233	−3.5532
C	−2.8544	−6.5565	−2.9466
C	−2.4706	−4.3034	−3.6464
C	−4.0963	−6.1846	−2.4260
C	−3.6887	−3.9011	−3.1057
C	−4.5004	−4.8491	−2.4808
O	−5.6869	−4.4104	−1.9401
H	−0.7709	−5.3476	−6.1190
H	−0.3121	−6.9231	−3.5151
H	0.0891	−5.2366	−3.7408
H	−2.5339	−7.5914	−2.8588
H	−1.8566	−3.5589	−4.1384
H	−4.7331	−6.9273	−1.9510
H	−4.0008	−2.8611	−3.1185
H	−6.0680	−5.1089	−1.3908
C	4.7811	−3.9255	−5.7923
C	5.0225	−4.7597	−4.5564
O	6.1288	−5.2119	−4.2645
C	5.1209	−2.4496	−5.5178
H	5.4214	−4.3144	−6.5883
H	6.1706	−2.3503	−5.2291
H	4.5088	−2.0461	−4.7037
H	4.9497	−1.8364	−6.4068
N	3.9412	−4.9057	−3.7513
C	4.0860	−5.4701	−2.4247
C	2.7824	−5.4070	−1.6420
C	2.2169	−3.9911	−1.4433
C	0.9626	−4.0678	−0.5744
C	3.2418	−3.0226	−0.8480
H	3.0845	−4.4272	−3.9822
H	4.8742	−4.9332	−1.8874
H	2.9655	−5.8520	−0.6562
H	2.0258	−6.0358	−2.1316
H	1.9121	−3.5914	−2.4214
H	1.2181	−4.4478	0.4203
H	0.5017	−3.0904	−0.4374
H	0.2118	−4.7358	−1.0111
H	4.1071	−2.8742	−1.5009
H	3.6109	−3.3824	0.1184
H	2.7910	−2.0452	−0.6761
C	9.0164	−5.4830	−1.1450
C	7.6757	−4.7457	−1.0344
C	7.7925	−3.2495	−1.2267
C	7.1150	−2.6154	−2.2740

C	8.5791	−2.4626	−0.3725
C	7.2023	−1.2342	−2.4548
C	8.6783	−1.0817	−0.5553
C	7.9810	−0.4586	−1.5956
H	9.7352	−5.1285	−0.4002
H	7.2318	−4.9512	−0.0509
H	6.9943	−5.1457	−1.7901
H	6.5388	−3.2233	−2.9612
H	9.1133	−2.9313	0.4494
H	6.6633	−0.7682	−3.2741
H	9.2929	−0.4841	0.1095
H	8.0514	0.6156	−1.7243
C	−2.4010	0.7643	0.6846
C	−1.2618	1.2481	0.0947
C	−1.3800	2.5358	−0.5352
N	−0.3274	3.1782	−0.9983
N	−2.5922	3.1406	−0.6764
C	−3.6456	2.6179	−0.0792
N	−3.5735	1.4696	0.6281
S	2.2958	−0.0355	−3.1383
C	1.8701	0.7449	−1.6202
C	−4.9442	3.3498	−0.1718
N	0.6837	0.2182	−1.1582
C	0.1123	−0.7727	−1.9892
C	−1.2113	−1.3848	−1.6609
C	0.8615	−1.0446	−3.0815
C	0.5904	−1.9883	−4.2095
C	0.0636	0.5099	0.1628
H	0.6377	2.9268	−0.7195
H	−0.4154	4.0684	−1.4811
H	−0.0875	−0.4382	0.6831
H	0.7865	1.0776	0.7356
H	−5.7652	2.8152	0.3015
H	−4.8362	4.3350	0.2923
H	−5.1851	3.5036	−1.2273
H	−2.4457	−0.1910	1.1977
H	−1.2925	−1.6905	−0.6154
H	−1.3539	−2.2768	−2.2690
H	−2.0418	−0.7076	−1.8821
H	0.0454	−2.8649	−3.8569
H	1.5263	−2.3355	−4.6610
C	1.6268	−3.3261	7.2057
C	0.8955	−1.9823	7.2319
C	1.6917	−0.8753	6.5349
O	2.0506	−1.1474	5.3160
O	1.9255	0.1913	7.1209
C	−8.3936	2.6301	4.2131
C	−6.9161	2.3998	3.8602
C	−6.6495	1.8552	2.4403
O	−7.4257	2.2138	1.5141
O	−5.6110	1.1344	2.3004
H	−8.5113	3.3409	5.0366
H	−8.9301	3.0002	3.3404
H	−6.4215	1.7471	4.5847
H	−6.3736	3.3558	3.9007
C	−0.9220	1.6175	5.5970
C	−0.6906	1.0174	4.2047
C	0.5707	1.5084	3.5725
C	0.8044	2.4757	2.6227
N	1.8146	1.0041	3.8946
C	2.7271	1.6338	3.1329
N	2.1595	2.5394	2.3356
H	−0.0975	1.3627	6.2690
H	−0.6630	−0.0765	4.2723
H	−1.5279	1.2674	3.5431
H	0.0905	3.1079	2.1139
H	3.7784	1.3961	3.1598
H	2.3756	2.6625	0.7589
C	5.4632	6.8013	3.4989
C	5.6476	5.3407	3.9089
C	5.9584	4.3713	2.7538
C	6.1072	2.9524	3.3224
C	4.8911	4.4412	1.6541
H	4.5833	6.9337	2.8624
H	4.7360	4.9919	4.4131
H	6.4525	5.2706	4.6526
H	6.9215	4.6619	2.3085
H	5.2515	2.7055	3.9589
H	6.1628	2.1949	2.5376
H	7.0071	2.8663	3.9408
H	3.8956	4.2307	2.0551
H	5.0797	3.7087	0.8663
H	4.8699	5.4282	1.1803
H	0.0004	−1.5168	−5.0050
H	−8.8693	1.6893	4.5130
H	11.8104	4.5224	0.6737
H	12.2451	5.6176	−0.6588
H	−10.1945	3.5115	−4.5449
H	−9.0822	2.3097	−5.2266
H	−0.9912	2.7087	5.5367
H	−1.8531	1.2377	6.0300
H	6.3333	7.1703	2.9421
H	5.3340	7.4444	4.3762
H	−0.0625	−2.0796	6.7036
H	0.6826	−1.6615	8.2556
H	1.8685	−3.6052	6.1766
H	1.0220	−4.1219	7.6552
H	2.5694	−3.2686	7.7620
H	8.8805	−6.5590	−0.9951
H	9.4566	−5.3309	−2.1351
H	4.4291	−6.5071	−2.5156
H	0.5335	−6.5123	−5.8228
H	−1.1544	−7.0565	−5.8875
H	3.7399	−4.0173	−6.1258
H	2.0027	0.1854	4.5677
H	−4.4321	1.1818	1.1966
C	2.1966	7.0867	−2.3359
C	0.8512	6.4787	−2.0448
O	0.6048	5.3152	−2.3705
N	−0.0529	7.2402	−1.3771
C	−1.4232	6.7659	−1.2670
C	−1.9992	6.4826	−2.6667
O	−1.7596	7.2500	−3.5958
N	−2.7629	5.3729	−2.7510
C	−3.2941	4.9195	−4.0268
C	−2.9778	3.4444	−4.2972
C	−1.4847	3.0840	−4.3115
C	−1.3081	1.5713	−4.4875
C	−0.6946	3.8708	−5.3621
C	3.7868	2.2911	−1.9506
C	3.6698	3.6305	−2.3552
C	4.6216	4.2037	−3.1945
C	5.7081	3.4527	−3.6484
C	5.8356	2.1223	−3.2485
C	4.8893	1.5483	−2.4002
C	2.7323	1.7170	−1.0597
O	2.1422	2.6810	−0.2406
C	3.8524	0.5402	0.4640
O	4.8974	1.1089	0.5265
O	3.0660	−0.2750	0.8496
H	2.9526	6.5732	−1.7345
H	2.4311	6.9105	−3.3877
H	−1.4662	5.8838	−0.6225
H	−2.0169	7.5515	−0.7933
H	−3.4889	2.8268	−3.5488
H	−3.4291	3.1785	−5.2637
H	−1.0665	3.3465	−3.3380
H	0.3564	3.5646	−5.3575
H	−1.0946	3.6968	−6.3698
H	−0.7210	4.9452	−5.1616
H	−1.6802	1.2417	−5.4665
H	−1.8582	1.0158	−3.7199
H	−0.2533	1.2886	−4.4148
H	−2.8725	5.5726	−4.7922
H	2.8092	4.1986	−2.0250
H	4.5094	5.2385	−3.5056
H	6.4509	3.9000	−4.3024
H	6.6878	1.5367	−3.5788
H	5.0272	0.5279	−2.0642
C	−5.2070	−3.3213	3.2677
C	−3.6946	−3.4212	3.3510
O	−3.0674	−2.8484	4.2385
C	−5.7075	−2.0039	2.6241
C	−5.4645	−1.9578	1.1298
N	−6.3844	−2.5437	0.3401
O	−4.4393	−1.4382	0.6513
N	−3.0722	−4.2311	2.4508
C	−1.6512	−4.4795	2.6154
C	−0.7982	−3.2478	2.2816
O	−1.1605	−2.3695	1.4876
C	−1.3946	−5.6379	1.6330
C	−2.3762	−5.3407	0.4943
C	−3.6237	−4.8289	1.2282
N	0.4004	−3.2817	2.8718
C	1.4740	−2.3659	2.5919
C	2.7628	−3.0386	3.0585
O	2.7459	−4.1335	3.6289
N	3.8758	−2.3348	2.7860
C	5.2089	−2.7427	3.1758
C	5.7877	−1.9151	4.3453
O	6.9977	−1.9481	4.5759
C	6.1398	−2.6751	1.9701
O	6.0071	−1.3767	1.4100
N	4.8986	−1.2156	5.0748
C	5.3259	−0.4994	6.2626
H	−6.7816	−1.9061	2.8115
H	−5.2126	−1.1386	3.0658
H	−5.6477	−4.1800	2.7508
H	−1.6502	−6.5844	2.1226
H	−0.3548	−5.6941	1.3112
H	−2.5884	−6.2058	−0.1342
H	−1.9850	−4.5470	−0.1469
H	−4.3074	−5.6549	1.4746
H	−4.1670	−4.0956	0.6327
H	−1.4488	−4.7575	3.6537
H	1.3529	−1.4316	3.1413
H	1.5214	−2.1390	1.5253
H	7.1607	−2.8667	2.3175
H	5.8486	−3.4496	1.2450
H	6.6437	−1.2880	0.6873
H	5.1398	−3.7779	3.5241
H	6.1014	0.2300	6.0085
H	2.2490	8.1578	−2.1258
H	−4.3839	5.0583	−4.0454
H	−2.7885	4.7188	−1.9670
H	0.0981	8.2368	−1.3607
H	5.7465	−1.1811	7.0117
H	4.4560	0.0111	6.6795
H	3.8885	−1.3217	4.9537
H	0.6752	−4.0444	3.4837
H	−7.2212	−3.0012	0.6848
H	−6.1681	−2.6506	−0.6427
H	3.7913	−1.4872	2.2367
H	−5.5554	−3.3387	4.3032
C	−10.9132	−5.0412	1.4375
C	−9.9728	−4.2950	0.5025
O	−8.7701	−4.1788	0.7497
C	−11.3900	−4.1404	2.5885
N	−10.5463	−3.7512	−0.5986
C	−9.7985	−2.9523	−1.5585
C	−8.8830	6.9357	3.6569
C	−8.6283	5.6897	2.8187
C	−8.3396	5.9791	1.3457
N	−7.9574	4.8866	0.6329
O	−8.4601	7.0991	0.8547
H	−10.5333	−3.7468	3.1418
H	−12.0201	−4.7026	3.2828
H	−11.9688	−3.2909	2.2130
H	−11.7726	−5.4386	0.8848
H	−8.9166	−3.5013	−1.9010
H	−7.7953	5.1099	3.2274
H	−9.4977	5.0236	2.8584
H	−7.8336	3.9534	1.0392
H	−7.7657	5.0212	−0.3481
H	−9.7060	7.5209	3.2393
H	−9.1299	6.6629	4.6876
H	−8.0032	7.5853	3.6728
H	−10.3532	−5.8912	1.8360
H	−9.4558	−2.0091	−1.1203
H	−10.4377	−2.7362	−2.4149
H	−11.5413	−3.8518	−0.7220

Int4–CO_2

C	11.4723	5.1059	−0.1307
C	10.8977	4.0152	−1.0474
C	9.7172	3.3193	−0.4422
C	8.3923	3.4032	−0.8038
N	9.8547	2.4889	0.6583
C	8.6334	2.0820	0.9501
N	7.7105	2.6103	0.0961
H	10.7288	5.8883	0.0532
H	11.6793	3.2753	−1.2599

H	10.6040	4.4514	−2.0096
H	7.8775	3.9331	−1.5875
H	8.3568	1.4111	1.7507
H	6.7270	2.3867	0.0787
C	−9.4779	2.8160	−4.1894
C	−8.3770	3.2977	−3.2257
C	−7.3698	2.2253	−2.8645
C	−6.3480	1.8625	−3.7559
C	−7.4133	1.5678	−1.6305
C	−5.4200	0.8751	−3.4363
C	−6.4894	0.5789	−1.2906
C	−5.4882	0.2160	−2.2000
O	−4.5615	−0.7503	−1.9485
H	−10.0438	1.9901	−3.7462
H	−8.8435	3.6762	−2.3084
H	−7.8576	4.1497	−3.6831
H	−6.2684	2.3714	−4.7142
H	−8.1370	1.8582	−0.8750
H	−4.6234	0.6112	−4.1245
H	−6.5227	0.1501	−0.2963
H	−4.5537	−1.0188	−0.9914
C	−0.5177	−6.1596	−5.5897
C	−0.7319	−6.0544	−4.0679
C	−2.1289	−5.6703	−3.6103
C	−2.9776	−6.6033	−3.0069
C	−2.5957	−4.3493	−3.7048
C	−4.2195	−6.2324	−2.4838
C	−3.8133	−3.9481	−3.1612
C	−4.6228	−4.8971	−2.5319
O	−5.8051	−4.4594	−1.9780
H	−0.7410	−5.2090	−6.0852
H	−0.4666	−7.0119	−3.6052
H	−0.0203	−5.3225	−3.6648
H	−2.6576	−7.6385	−2.9194
H	−1.9811	−3.6027	−4.1952
H	−4.8547	−6.9769	−2.0090
H	−4.1240	−2.9078	−3.1717
H	−6.1882	−5.1611	−1.4350
C	4.7682	−3.8604	−5.8292
C	5.0164	−4.7099	−4.6037
O	6.1257	−5.1575	−4.3153
C	5.0765	−2.3835	−5.5236
H	5.4231	−4.2233	−6.6255
H	6.1175	−2.2725	−5.2083
H	4.4367	−2.0033	−4.7198
H	4.9156	−1.7588	−6.4065
N	3.9347	−4.8738	−3.8014
C	4.0840	−5.4343	−2.4730
C	2.7641	−5.4248	−1.7122
C	2.1270	−4.0358	−1.5397
C	0.8583	−4.1564	−0.6979
C	3.0839	−3.0107	−0.9273
H	3.0839	−4.3783	−4.0198
H	4.8392	−4.8676	−1.9199
H	2.9498	−5.8521	−0.7188
H	2.0467	−6.0923	−2.2091
H	1.8265	−3.6557	−2.5263
H	1.1111	−4.5021	0.3103
H	0.3497	−3.1983	−0.5934
H	0.1502	−4.8695	−1.1346
H	3.9698	−2.8369	−1.5446
H	3.4251	−3.3307	0.0638
H	2.5781	−2.0516	−0.8127
C	9.0211	−5.4828	−1.2204
C	7.6737	−4.7729	−1.0425
C	7.7471	−3.2692	−1.2040
C	6.9931	−2.6264	−2.1928
C	8.5629	−2.4814	−0.3778
C	7.0305	−1.2392	−2.3399
C	8.6151	−1.0928	−0.5300
C	7.8378	−0.4633	−1.5087
H	9.7624	−5.1337	−0.4943
H	7.2702	−5.0096	−0.0485
H	6.9724	−5.1704	−1.7802
H	6.3943	−3.2302	−2.8634
H	9.1616	−2.9551	0.3954
H	6.4358	−0.7697	−3.1168
H	9.2588	−0.4961	0.1082
H	7.8710	0.6144	−1.6191
C	−2.5045	0.7378	0.6052
C	−1.3805	1.2286	−0.0034
C	−1.5163	2.5318	−0.6004
N	−0.4815	3.1823	−1.0886
N	−2.7256	3.1540	−0.6789
C	−3.7648	2.6165	−0.0727
N	−3.6773	1.4461	0.5948
S	2.2199	−0.1355	−3.2682
C	1.7531	0.7508	−1.7900
C	−5.0596	3.3587	−0.1067
N	0.5307	0.2257	−1.3262
C	−0.0167	−0.7859	−2.1448
C	−1.3469	−1.3848	−1.8133
C	0.7428	−1.1007	−3.2137
C	0.4911	−2.0669	−4.3243
C	−0.0573	0.4753	0.0081
H	0.4844	2.9258	−0.8351
H	−0.5801	4.1058	−1.4938
H	−0.2099	−0.4805	0.5163
H	0.6753	1.0265	0.5854
H	−5.8797	2.8008	0.3385
H	−4.9429	4.3124	0.4172
H	−5.3057	3.5789	−1.1487
H	−2.5403	−0.2271	1.0992
H	−1.4229	−1.6998	−0.7697
H	−1.5105	−2.2693	−2.4259
H	−2.1702	−0.6936	−2.0151
H	−0.1386	−2.8909	−3.9878
H	1.4330	−2.4997	−4.6843
C	1.6871	−3.3724	7.1909
C	0.9962	−2.0117	7.2925
C	1.7772	−0.9119	6.5709
O	2.1110	−1.1971	5.3467
O	2.0237	0.1610	7.1387
C	−8.3210	2.6596	4.3108
C	−6.8735	2.4000	3.8745
C	−6.7151	1.8232	2.4526
O	−7.5341	2.1973	1.5708
O	−5.7155	1.0612	2.2623
H	−8.3794	3.3761	5.1359
H	−8.9001	3.0349	3.4683
H	−6.3448	1.7523	4.5791
H	−6.3151	3.3477	3.8602
C	−0.8468	1.5985	5.6441
C	−0.6597	0.9658	4.2566
C	0.5913	1.4129	3.5698
C	0.8091	2.3194	2.5570
N	1.8396	0.9190	3.8899
C	2.7368	1.4996	3.0707
N	2.1562	2.3582	2.2336
H	−0.0110	1.3439	6.3019
H	−0.6526	−0.1268	4.3493
H	−1.5104	1.2150	3.6122
H	0.0872	2.9268	2.0303
H	3.7918	1.2777	3.1142
H	2.2926	2.5240	0.6119
C	5.5515	6.7724	3.5615
C	5.6625	5.3005	3.9627
C	5.9442	4.3257	2.8048
C	6.0859	2.9052	3.3701
C	4.8621	4.3978	1.7194
H	4.6944	6.9494	2.9050
H	4.7324	4.9891	4.4576
H	6.4571	5.1912	4.7128
H	6.9043	4.6054	2.3465
H	5.2184	2.6508	3.9876
H	6.1625	2.1537	2.5798
H	6.9748	2.8158	4.0032
H	3.8733	4.1679	2.1269
H	5.0473	3.6802	0.9151
H	4.8218	5.3890	1.2566
H	0.0070	−1.5935	−5.1881
H	−8.7952	1.7288	4.6426
H	11.7480	4.6744	0.8354
H	12.3609	5.5718	−0.5708
H	−10.1778	3.6239	−4.4274
H	−9.0424	2.4533	−5.1263
H	−0.9015	2.6893	5.5645
H	−1.7733	1.2405	6.1043
H	6.4512	7.1062	3.0306
H	5.4285	7.4125	4.4416
H	0.0057	−2.0705	6.8212
H	0.8519	−1.7079	8.3332
H	1.8564	−3.6351	6.1438
H	1.0905	−4.1622	7.6614
H	2.6641	−3.3526	7.6871
H	8.9088	−6.5640	−1.0912
H	9.4207	−5.2998	−2.2223
H	4.4695	−6.4558	−2.5620
H	0.5205	−6.4240	−5.8137
H	−1.1697	−6.9222	−6.0268
H	3.7317	−3.9662	−6.1721
H	2.0411	0.1249	4.5879
H	−4.5233	1.1382	1.1607
C	2.2549	7.1298	−2.2523
C	0.9054	6.5493	−1.9248
O	0.6661	5.3607	−2.1304
N	−0.0039	7.3741	−1.3321
C	−1.3758	6.9065	−1.2132
C	−1.9454	6.5857	−2.6065
O	−1.6730	7.3152	−3.5582
N	−2.7352	5.4929	−2.6667
C	−3.2551	5.0049	−3.9342
C	−2.7256	3.6081	−4.2860
C	−1.2031	3.5043	−4.4702
C	−0.7852	2.0353	−4.5995
C	−0.7036	4.3445	−5.6508
C	3.6863	2.3534	−1.9934
C	3.8686	3.7524	−1.9786
C	4.9475	4.3396	−2.6295
C	5.8876	3.5535	−3.3031
C	5.7443	2.1658	−3.2892
C	4.6643	1.5732	−2.6391
C	2.5000	1.8042	−1.3228
O	1.9710	2.6340	−0.3474
C	4.2871	0.3121	0.5589
O	5.1905	1.0283	0.3830
O	3.3573	−0.3700	0.7451
H	2.9416	6.9236	−1.4243
H	2.6391	6.6271	−3.1401
H	−1.4157	6.0420	−0.5455
H	−1.9707	7.7042	−0.7610
H	−3.0475	2.9072	−3.5061
H	−3.2267	3.2830	−5.2095
H	−0.7190	3.9007	−3.5745
H	0.3769	4.2208	−5.7783
H	−1.1890	4.0406	−6.5877
H	−0.8966	5.4093	−5.4906
H	−1.2319	1.5725	−5.4896
H	−1.1021	1.4563	−3.7262
H	0.3020	1.9374	−4.6777
H	−2.9857	5.7438	−4.6896
H	3.1269	4.3571	−1.4741
H	5.0535	5.4211	−2.6191
H	6.7229	4.0142	−3.8221
H	6.4858	1.5379	−3.7746
H	4.6107	0.4923	−2.5938
C	−5.1679	−3.2973	3.2904
C	−3.6566	−3.4257	3.3136
O	−2.9793	−2.8505	4.1626
C	−5.6920	−2.0006	2.6281
C	−5.4856	−1.9862	1.1285
N	−6.4251	−2.5792	0.3663
O	−4.4669	−1.4841	0.6196
N	−3.0912	−4.2730	2.4106
C	−1.6728	−4.5584	2.5333
C	−0.8053	−3.3346	2.2097
O	−1.1397	−2.4632	1.3995
C	−1.4651	−5.7024	1.5231
C	−2.4829	−5.3762	0.4258
C	−3.6977	−4.8717	1.2148
N	0.3773	−3.3703	2.8378
C	1.4552	−2.4468	2.6082
C	2.7382	−3.1341	3.0709
O	2.7233	−4.2446	3.6048
N	3.8541	−2.4158	2.8356
C	5.1928	−2.8308	3.1973
C	5.8014	−2.0096	4.3558
O	7.0147	−2.0597	4.5636
C	6.1062	−2.7580	1.9801
O	6.0324	−1.4292	1.4695
N	4.9353	−1.2848	5.0880
C	5.3944	−0.5541	6.2551
H	−6.7612	−1.9039	2.8417

```
H  -5.1947  -1.1209   3.0361
H  -5.6388  -4.1692   2.8242
H  -1.7137  -6.6542   2.0061
H  -0.4381  -5.7645   1.1638
H  -2.7243  -6.2266  -0.2117
H  -2.1083  -4.5712  -0.2107
H  -4.3660  -5.7012   1.4911
H  -4.2708  -4.1408   0.6464
H  -1.4527  -4.8652   3.5604
H   1.3275  -1.5329   3.1919
H   1.5141  -2.1825   1.5491
H   7.1238  -2.9997   2.3006
H   5.7732  -3.4844   1.2258
H   6.7431  -1.3141   0.8213
H   5.1224  -3.8693   3.5350
H   6.1788   0.1557   5.9745
H   2.2309   8.2104  -2.4169
H  -4.3512   4.9659  -3.8824
H  -2.8014   4.8606  -1.8675
H   0.1219   8.3649  -1.4761
H   5.8141  -1.2285   7.0111
H   4.5400  -0.0203   6.6751
H   3.9204  -1.3890   4.9933
H   0.6236  -4.1326   3.4598
H  -7.2568  -3.0328   0.7312
H  -6.2284  -2.7019  -0.6197
H   3.7596  -1.5379   2.3483
H  -5.4705  -3.2773   4.3404
C -10.9954  -4.9662   1.4348
C -10.0178  -4.2553   0.5111
O  -8.8086  -4.2131   0.7563
C -11.3582  -4.0806   2.6370
N -10.5660  -3.6519  -0.5707
C  -9.7835  -2.8598  -1.5069
C  -8.7928   6.9727   3.7990
C  -8.5796   5.7226   2.9554
C  -8.3277   6.0061   1.4745
N  -8.0141   4.9025   0.7455
O  -8.4153   7.1329   0.9921
H -10.4540  -3.7898   3.1781
H -12.0169  -4.6149   3.3269
H -11.8683  -3.1672   2.3160
H -11.9007  -5.2611   0.8914
H  -8.9082  -3.4262  -1.8366
H  -7.7413   5.1341   3.3404
H  -9.4548   5.0666   3.0225
H  -7.9085   3.9624   1.1418
H  -7.8358   5.0374  -0.2380
H  -9.6245   7.5657   3.4102
H  -9.0061   6.7040   4.8382
H  -7.9069   7.6137   3.7822
H -10.5019  -5.8791   1.7787
H  -9.4283  -1.9276  -1.0548
H -10.4001  -2.6226  -2.3744
H -11.5668  -3.6857  -0.6835
```

Int4

```
C  11.5246   4.9600  -0.9347
C  10.6614   3.8448  -1.5522
C   9.7100   3.1749  -0.5991
C   8.3454   3.0132  -0.7248
N  10.1479   2.5526   0.5580
C   9.0651   2.0289   1.1101
N   7.9473   2.2779   0.3730
H  10.8998   5.7833  -0.5729
H  11.3314   3.0816  -1.9715
H  10.0862   4.2443  -2.3965
H   7.6393   3.3477  -1.4679
H   9.0205   1.4643   2.0319
H   7.0202   1.8850   0.5489
C  -9.4526   2.1366  -4.4859
C  -8.2169   2.6926  -3.7584
C  -7.3200   1.6212  -3.1737
C  -6.7058   0.6696  -4.0012
C  -7.0705   1.5427  -1.7993
C  -5.8752  -0.3156  -3.4798
C  -6.2267   0.5702  -1.2576
C  -5.6151  -0.3620  -2.1035
O  -4.7733  -1.3402  -1.6628
H -10.0749   1.5533  -3.7994
H  -8.5394   3.3659  -2.9559
H  -7.6419   3.3092  -4.4630
H  -6.8724   0.7037  -5.0754
H  -7.5319   2.2427  -1.1095
H  -5.3963  -1.0470  -4.1222
H  -6.0530   0.5599  -0.1866
H  -4.5642  -1.2390  -0.6963
C  -0.4933  -6.9456  -4.7160
C  -0.4191  -6.0220  -3.4901
C  -1.7675  -5.6491  -2.9100
C  -2.0521  -5.8539  -1.5568
C  -2.7711  -5.0678  -3.7013
C  -3.2855  -5.5023  -1.0083
C  -4.0040  -4.7031  -3.1691
C  -4.2689  -4.9259  -1.8147
O  -5.5127  -4.6018  -1.3454
H  -1.0380  -6.4745  -5.5400
H   0.1790  -6.5034  -2.7088
H   0.1233  -5.1070  -3.7620
H  -1.2993  -6.3017  -0.9146
H  -2.5842  -4.8876  -4.7562
H  -3.4857  -5.6878   0.0429
H  -4.7671  -4.2341  -3.7801
H  -5.4975  -4.5637  -0.3777
C   4.7880  -4.7003  -5.3066
C   5.0754  -5.2987  -3.9499
O   6.2178  -5.5396  -3.5604
C   4.8653  -3.1639  -5.2400
H   5.5369  -5.0846  -6.0038
H   5.8525  -2.8494  -4.8898
H   4.1176  -2.7541  -4.5532
H   4.6960  -2.7210  -6.2257
N   3.9832  -5.4827  -3.1675
C   4.1326  -5.8018  -1.7619
C   2.8567  -5.5250  -0.9770
C   2.3353  -4.0786  -1.0424
C   1.1363  -3.9468  -0.1020
C   3.4209  -3.0490  -0.6996
H   3.0840  -5.1655  -3.4964
H   4.9586  -5.2111  -1.3590
H   3.0575  -5.7724   0.0726
H   2.0629  -6.2096  -1.3085
H   1.9791  -3.8688  -2.0630
H   1.4510  -4.0889   0.9374
H   0.6810  -2.9624  -0.1814
H   0.3586  -4.6814  -0.3368
H   4.2305  -3.0420  -1.4361
H   3.8496  -3.2621   0.2846
H   2.9988  -2.0408  -0.6685
C   9.0751  -5.6786  -0.5517
C   7.7917  -4.8376  -0.4793
C   8.0244  -3.3716  -0.7766
C   7.4585  -2.7750  -1.9099
C   8.8316  -2.5842   0.0571
C   7.6919  -1.4286  -2.2014
C   9.0801  -1.2451  -0.2391
C   8.5078  -0.6593  -1.3713
H   9.8289  -5.3242   0.1589
H   7.3463  -4.9433   0.5191
H   7.0765  -5.2374  -1.2021
H   6.8551  -3.3847  -2.5749
H   9.2715  -3.0261   0.9479
H   7.2371  -0.9849  -3.0825
H   9.7113  -0.6473   0.4102
H   8.7037   0.3856  -1.5899
C  -2.3978   0.6979   0.5400
C  -1.2621   0.9178  -0.1968
C  -1.2044   2.1517  -0.9183
N  -0.1164   2.5342  -1.5788
N  -2.2706   2.9979  -0.9378
C  -3.3386   2.7130  -0.2113
N  -3.4235   1.5959   0.5395
S   1.6592  -0.5411  -3.6696
C   1.6042  -0.0035  -1.9663
C  -4.4760   3.6805  -0.1827
N   0.4590  -0.5385  -1.3594
C  -0.2820  -1.4205  -2.1766
C  -1.5377  -2.0382  -1.6458
C   0.2205  -1.5589  -3.4212
C  -0.2802  -2.3729  -4.5675
C  -0.0858  -0.0259  -0.0953
H   0.7151   1.9503  -1.6079
H  -0.0561   3.4339  -2.0600
H  -0.3921  -0.8663   0.5270
H   0.7317   0.4725   0.4193
H  -5.4283   3.1784  -0.0099
H  -4.3281   4.3900   0.6395
H  -4.4970   4.2480  -1.1137
H  -2.5424  -0.1870   1.1495
H  -1.4321  -2.3729  -0.6123
H  -1.8206  -2.9005  -2.2472
H  -2.3720  -1.3329  -1.6885
H  -1.1790  -2.9221  -4.2841
H   0.4699  -3.0972  -4.9106
C   1.8033  -2.4410   7.5480
C   1.3108  -1.0045   7.3921
C   1.9611  -0.3045   6.1986
O   2.2540  -1.0225   5.1980
O   2.1608   0.9424   6.2797
C  -8.2324   3.1411   3.9473
C  -6.7449   2.8955   3.6537
C  -6.4228   2.3008   2.2673
O  -7.1139   2.6688   1.2824
O  -5.4189   1.5173   2.2179
H  -8.3697   3.8510   4.7688
H  -8.7321   3.5244   3.0587
H  -6.2809   2.2662   4.4177
H  -6.2042   3.8530   3.6826
C  -0.7476   2.2722   5.3569
C  -0.6019   1.6174   3.9799
C   0.6439   2.0218   3.2599
C   0.8455   2.5124   1.9882
N   1.9080   1.8676   3.7930
C   2.8020   2.2305   2.8436
N   2.1968   2.6263   1.7230
H   0.0865   1.9981   6.0077
H  -0.6206   0.5257   4.0953
H  -1.4592   1.8666   3.3449
H   0.1032   2.7896   1.2569
H   3.8701   2.1925   2.9936
H   2.6787   1.6506   0.3727
C   5.6298   7.1141   2.5401
C   5.8001   5.8352   3.3608
C   6.2041   4.5902   2.5510
C   6.3357   3.3857   3.4934
C   5.2210   4.3224   1.4025
H   4.8064   7.0266   1.8249
H   4.8578   5.6153   3.8827
H   6.5498   6.0037   4.1455
H   7.1934   4.7743   2.1070
H   5.4068   3.2240   4.0533
H   6.5670   2.4617   2.9608
H   7.1282   3.5489   4.2320
H   4.1838   4.3189   1.7498
H   5.4067   3.3513   0.9393
H   5.3051   5.0819   0.6187
H  -0.5321  -1.7384  -5.4262
H  -8.7310   2.2058   4.2257
H  12.0853   4.5684  -0.0817
H  12.2331   5.3598  -1.6678
H -10.0643   2.9448  -4.9005
H  -9.1622   1.4773  -5.3099
H  -0.7693   3.3636   5.2674
H  -1.6790   1.9496   5.8325
H   6.5390   7.3424   1.9709
H   5.4170   7.9736   3.1851
H   0.2240  -0.9890   7.2262
H   1.4952  -0.4072   8.2897
H   1.6292  -3.0093   6.6318
H   1.3030  -2.9506   8.3794
H   2.8813  -2.4648   7.7377
H   8.8661  -6.7299  -0.3274
H   9.5096  -5.6248  -1.5547
H   4.4235  -6.8541  -1.6506
H   0.5099  -7.1923  -5.0787
H  -1.0075  -7.8796  -4.4694
H   3.8005  -5.0125  -5.6683
H   2.1240   1.5079   4.7673
H  -4.2992   1.4679   1.1740
C   2.2895   6.6749  -3.2433
C   1.0343   5.9798  -2.7651
O   0.8530   4.7714  -2.9440
```

N	0.1136	6.7577	-2.1382
C	-1.2330	6.2441	-1.9450
C	-1.8803	5.9281	-3.3059
O	-1.7076	6.6778	-4.2636
N	-2.6298	4.8054	-3.3336
C	-3.2304	4.3402	-4.5782
C	-3.4528	2.8299	-4.5804
C	-2.1866	1.9841	-4.3748
C	-2.5317	0.4956	-4.4309
C	-1.0574	2.3373	-5.3485
C	3.6295	1.3981	-2.2866
C	3.2611	2.3922	-3.2099
C	4.2213	3.0259	-3.9963
C	5.5680	2.6817	-3.8730
C	5.9460	1.6937	-2.9614
C	4.9882	1.0572	-2.1749
C	2.6100	0.7495	-1.4293
O	2.8411	0.7569	-0.0552
H	3.1372	6.0007	-3.1092
H	2.1834	6.8821	-4.3132
H	-1.2121	5.3699	-1.2913
H	-1.8221	7.0179	-1.4459
H	-4.1913	2.5607	-3.8139
H	-3.9120	2.5663	-5.5428
H	-1.8110	2.1877	-3.3701
H	-0.2032	1.6697	-5.1936
H	-1.3847	2.2305	-6.3909
H	-0.7013	3.3616	-5.2041
H	-2.9003	0.2136	-5.4258
H	-3.3090	0.2408	-3.7067
H	-1.6523	-0.1087	-4.2054
H	-2.5732	4.6469	-5.3963
H	2.2249	2.6973	-3.2997
H	3.9120	3.7959	-4.6972
H	6.3190	3.1768	-4.4819
H	6.9891	1.4163	-2.8606
H	5.2983	0.2844	-1.4825
C	-5.0804	-2.8991	3.7254
C	-3.5906	-2.5889	3.9232
O	-3.2282	-1.5147	4.3952
C	-5.8030	-1.8078	2.9071
C	-5.4711	-1.8853	1.4322
N	-6.3165	-2.5839	0.6400
O	-4.4405	-1.3630	0.9733
N	-2.7039	-3.5881	3.6523
C	-1.2852	-3.4115	3.9394
C	-0.5906	-2.5249	2.8975
O	-1.0712	-2.3888	1.7644
C	-0.7372	-4.8470	3.8047
C	-1.5682	-5.4040	2.6373
C	-2.9647	-4.7983	2.8621
N	0.6031	-2.0494	3.2846
C	1.5306	-1.4045	2.3859
C	2.9531	-1.9107	2.5986
O	3.2192	-3.1011	2.7449
N	3.8707	-0.9172	2.5248
C	5.3033	-1.0836	2.6132
C	5.9079	-0.4122	3.8706
O	7.0982	-0.0887	3.8887
C	5.9723	-0.5655	1.3395
O	5.5424	0.7681	1.0380
N	5.0580	-0.2599	4.9083
C	5.5004	0.2234	6.2090
H	-6.8833	-1.9157	3.0420
H	-5.4929	-0.8178	3.2509
H	-5.2561	-3.8839	3.2869
H	-0.9376	-5.4026	4.7270
H	0.3388	-4.8563	3.6187
H	-1.5918	-6.4964	2.6084
H	-1.1620	-5.0390	1.6914
H	-3.6162	-5.4896	3.4126
H	-3.4490	-4.5420	1.9156
H	-1.1544	-2.9886	4.9385
H	1.4807	-0.3204	2.4893
H	1.2588	-1.6651	1.3667
H	7.0493	-0.5322	1.4894
H	5.7526	-1.2381	0.5035
H	4.6744	0.7246	0.5921
H	5.4893	-2.1597	2.7035
H	6.3610	0.8789	6.0650
H	2.4784	7.6149	-2.7194
H	-4.1878	4.8530	-4.7414
H	-2.5847	4.1768	-2.5318
H	0.2058	7.7554	-2.2573
H	5.8023	-0.6042	6.8641
H	4.6779	0.7698	6.6735
H	4.0903	-0.5832	4.8310
H	0.9196	-2.0569	4.2550
H	-7.2283	-2.9054	0.9624
H	-6.1404	-2.5523	-0.3592
H	3.5250	0.0283	2.4557
H	-5.5110	-2.9111	4.7323
C	-10.8236	-4.7944	2.1892
C	-9.9116	-4.1143	1.1868
O	-8.7767	-3.7504	1.5073
C	-11.2747	-3.7647	3.2314
N	-10.4430	-3.8725	-0.0325
C	-9.7321	-3.1203	-1.0520
C	-8.7128	7.3440	2.8556
C	-8.4343	6.0597	2.0873
C	-8.0613	6.2860	0.6225
N	-7.5979	5.1685	0.0039
O	-8.1899	7.3716	0.0625
H	-10.4026	-3.3091	3.7060
H	-11.8881	-4.2328	4.0062
H	-11.8630	-2.9674	2.7664
H	-11.6876	-5.2553	1.6971
H	-8.8537	-3.6687	-1.4089
H	-7.6372	5.4814	2.5635
H	-9.3180	5.4102	2.1009
H	-7.4758	4.2796	0.4969
H	-7.3509	5.2416	-0.9710
H	-9.4887	7.9301	2.3572
H	-9.0371	7.1213	3.8768
H	-7.8191	7.9727	2.9078
H	-10.2491	-5.5911	2.6704
H	-9.3923	-2.1594	-0.6554
H	-10.4016	-2.9400	-1.8934
H	-11.3767	-4.1972	-0.2255

TS5

C	11.5123	5.0490	-0.7538
C	10.6644	3.9603	-1.4379
C	9.6841	3.2589	-0.5370
C	8.3355	3.0419	-0.7374
N	10.0806	2.6607	0.6465
C	8.9906	2.0979	1.1433
N	7.9063	2.2983	0.3436
H	10.8800	5.8626	-0.3836
H	11.3458	3.2113	-1.8652
H	10.1160	4.3914	-2.2844
H	7.6581	3.3434	-1.5216
H	8.9174	1.5416	2.0685
H	6.9785	1.8942	0.4894
C	-9.4244	2.1666	-4.4917
C	-8.2093	2.7738	-3.7660
C	-7.2569	1.7424	-3.1957
C	-6.5046	0.9143	-4.0418
C	-7.0894	1.5817	-1.8157
C	-5.6288	-0.0399	-3.5337
C	-6.2101	0.6347	-1.2851
C	-5.4743	-0.1840	-2.1484
O	-4.6111	-1.1497	-1.7142
H	-10.0142	1.5490	-3.8064
H	-8.5588	3.4238	-2.9559
H	-7.6692	3.4222	-4.4692
H	-6.5970	1.0241	-5.1201
H	-7.6339	2.2026	-1.1107
H	-5.0439	-0.6745	-4.1912
H	-6.0957	0.5703	-0.2071
H	-4.4839	-1.1285	-0.7314
C	-0.4072	-6.8543	-4.8409
C	-0.4134	-6.2634	-3.4245
C	-1.7635	-5.7849	-2.9245
C	-2.0856	-5.8807	-1.5653
C	-2.7211	-5.2086	-3.7725
C	-3.3061	-5.4288	-1.0676
C	-3.9438	-4.7457	-3.2908
C	-4.2460	-4.8586	-1.9315
O	-5.4787	-4.4454	-1.5144
H	-0.6731	-6.1059	-5.5940
H	-0.0288	-7.0106	-2.7208
H	0.3032	-5.4306	-3.3828
H	-1.3709	-6.3247	-0.8780
H	-2.5162	-5.1174	-4.8345
H	-3.5349	-5.5336	-0.0114
H	-4.6728	-4.2902	-3.9516
H	-5.4882	-4.3377	-0.5506
C	4.8635	-4.5657	-5.3545
C	5.1111	-5.2673	-4.0391
O	6.2160	-5.6952	-3.7106
C	5.1418	-3.0572	-5.2251
H	5.5333	-5.0075	-6.0965
H	6.1756	-2.8862	-4.9134
H	4.4851	-2.5932	-4.4816
H	4.9834	-2.5471	-6.1796
N	4.0317	-5.3295	-3.2175
C	4.1930	-5.7398	-1.8361
C	2.9061	-5.5692	-1.0393
C	2.3218	-4.1444	-1.0113
C	1.1536	-4.1145	-0.0238
C	3.3739	-3.0831	-0.6602
H	3.1894	-4.8387	-3.4758
H	4.9957	-5.1548	-1.3777
H	3.1189	-5.8735	-0.0072
H	2.1415	-6.2615	-1.4175
H	1.9155	-3.9048	-2.0074
H	1.5217	-4.2321	1.0009
H	0.6061	-3.1755	-0.0710
H	0.4351	-4.9142	-0.2337
H	4.1515	-3.0018	-1.4256
H	3.8516	-3.3212	0.2948
H	2.9156	-2.0952	-0.5569
C	9.1270	-5.6097	-0.5925
C	7.7929	-4.8502	-0.5852
C	7.9245	-3.3718	-0.8900
C	7.2186	-2.8032	-1.9571
C	8.7497	-2.5371	-0.1224
C	7.3286	-1.4419	-2.2499
C	8.8777	-1.1812	-0.4211
C	8.1647	-0.6270	-1.4868
H	9.8245	-5.2130	0.1521
H	7.3115	-4.9774	0.3944
H	7.1331	-5.3036	-1.3298
H	6.5995	-3.4463	-2.5724
H	9.2985	-2.9522	0.7188
H	6.7633	-1.0216	-3.0769
H	9.5217	-0.5461	0.1789
H	8.2678	0.4306	-1.7045
C	-2.4177	0.6839	0.5196
C	-1.2630	0.9138	-0.1682
C	-1.1197	2.1830	-0.8482
N	0.0167	2.5490	-1.3862
N	-2.2202	3.0228	-0.9079
C	-3.3100	2.7263	-0.2315
N	-3.4335	1.5972	0.5068
S	1.8013	-0.7857	-3.4571
C	1.5429	-0.0493	-1.9095
C	-4.4594	3.6853	-0.2255
N	0.4172	-0.5476	-1.3443
C	-0.2533	-1.5321	-2.0938
C	-1.5315	-2.1227	-1.5977
C	0.3621	-1.7785	-3.2800
C	-0.0791	-2.6629	-4.4003
C	-0.1122	-0.0421	-0.0536
H	1.1805	1.8958	-1.3840
H	0.0247	3.4680	-1.8314
H	-0.4182	-0.9012	0.5423
H	0.7264	0.4343	0.4462
H	-5.4092	3.1763	-0.0599
H	-4.3254	4.4059	0.5892
H	-4.4785	4.2437	-1.1622
H	-2.5972	-0.2126	1.1011
H	-1.4859	-2.3748	-0.5365
H	-1.7589	-3.0359	-2.1457
H	-2.3666	-1.4336	-1.7479
H	-0.9387	-3.2603	-4.0956
H	0.7161	-3.3474	-4.7172
C	1.7849	-2.5746	7.5223
C	1.2512	-1.1517	7.3905
C	1.9266	-0.4026	6.2432
O	2.2320	-1.0797	5.2167

O	2.1328	0.8370	6.3834
C	−8.2628	3.0147	3.9669
C	−6.7870	2.7620	3.6228
C	−6.5157	2.2154	2.2044
O	−7.2047	2.6698	1.2534
O	−5.5630	1.3784	2.0957
H	−8.3677	3.6712	4.8365
H	−8.7740	3.4654	3.1173
H	−6.3128	2.0970	4.3496
H	−6.2351	3.7120	3.6736
C	−0.7816	2.1645	5.4068
C	−0.5996	1.4919	4.0453
C	0.6470	1.9266	3.3483
C	0.8580	2.4347	2.0860
N	1.9031	1.8031	3.9061
C	2.8058	2.2002	2.9789
N	2.2105	2.5886	1.8512
H	0.0450	1.9167	6.0774
H	−0.5907	0.4018	4.1747
H	−1.4506	1.7103	3.3915
H	0.1270	2.6936	1.3378
H	3.8710	2.1929	3.1508
H	2.6423	1.6846	0.4576
C	5.5826	7.0992	2.7247
C	5.7938	5.7896	3.4852
C	6.1882	4.5845	2.6125
C	6.3624	3.3446	3.5006
C	5.1748	4.3492	1.4841
H	4.7411	7.0286	2.0289
H	4.8701	5.5352	4.0243
H	6.5640	5.9355	4.2546
H	7.1615	4.7997	2.1473
H	5.4678	3.1721	4.1112
H	6.5450	2.4389	2.9207
H	7.2010	3.4687	4.1942
H	4.1495	4.3028	1.8621
H	5.3682	3.4064	0.9682
H	5.2161	5.1479	0.7367
H	−0.3748	−2.0707	−5.2739
H	−8.7737	2.0723	4.1953
H	12.0430	4.6248	0.1026
H	12.2454	5.4697	−1.4502
H	−10.0754	2.9493	−4.8952
H	−9.1067	1.5279	−5.3224
H	−0.8208	3.2535	5.2984
H	−1.7153	1.8346	5.8736
H	6.4722	7.3657	2.1412
H	5.3750	7.9273	3.4111
H	0.1717	−1.1666	7.1810
H	1.3819	−0.5756	8.3107
H	1.6732	−3.1149	6.5800
H	1.2659	−3.1296	8.3119
H	2.8532	−2.5687	7.7626
H	8.9699	−6.6709	−0.3732
H	9.6064	−5.5327	−1.5732
H	4.5213	−6.7847	−1.8115
H	0.5882	−7.2324	−5.0929
H	−1.1201	−7.6803	−4.9257
H	3.8328	−4.7311	−5.6920
H	2.1037	1.4482	4.8837
H	−4.3167	1.4477	1.0870
C	2.2812	6.7518	−3.0871
C	0.9911	6.0751	−2.6786
O	0.7717	4.8878	−2.9287
N	0.0813	6.8446	−2.0246
C	−1.2717	6.3398	−1.8534
C	−1.9104	6.0344	−3.2203
O	−1.7548	6.7991	−4.1691
N	−2.6420	4.8986	−3.2540
C	−3.2157	4.4097	−4.5020
C	−3.2320	2.8816	−4.5488
C	−1.8585	2.2083	−4.3984
C	−2.0183	0.6857	−4.4101
C	−0.8449	2.6754	−5.4483
C	3.3476	1.6065	−2.3621
C	2.9772	2.5429	−3.3398
C	3.9289	3.0786	−4.2064
C	5.2666	2.6928	−4.1077
C	5.6455	1.7632	−3.1371
C	4.6948	1.2221	−2.2720
C	2.3117	1.0683	−1.4157
O	2.8029	0.8627	−0.0916
H	3.1023	6.0448	−2.9564
H	2.2193	7.0072	−4.1501
H	−1.2647	5.4571	−1.2101
H	−1.8612	7.1143	−1.3557
H	−3.9033	2.5001	−3.7693
H	−3.6830	2.5792	−5.5039
H	−1.4496	2.4839	−3.4240
H	0.1009	2.1348	−5.3314
H	−1.2131	2.4937	−6.4665
H	−0.6230	3.7410	−5.3451
H	−2.4285	0.3397	−5.3679
H	−2.6968	0.3493	−3.6198
H	−1.0539	0.1943	−4.2601
H	−2.6299	4.8348	−5.3211
H	1.9521	2.8881	−3.4010
H	3.6213	3.8105	−4.9477
H	6.0104	3.1157	−4.7768
H	6.6839	1.4605	−3.0499
H	5.0069	0.5094	−1.5187
C	−5.0718	−3.0007	3.6479
C	−3.5918	−2.6678	3.8759
O	−3.2517	−1.5843	4.3420
C	−5.8033	−1.9119	2.8343
C	−5.4490	−1.9315	1.3616
N	−6.3333	−2.5148	0.5159
O	−4.3802	−1.4603	0.9420
N	−2.6871	−3.6610	3.6407
C	−1.2767	−3.4579	3.9465
C	−0.5796	−2.5854	2.8950
O	−1.0480	−2.4744	1.7533
C	−0.7068	−4.8882	3.8564
C	−1.5102	−5.4848	2.6893
C	−2.9177	−4.8916	2.8741
N	0.6044	−2.0893	3.2837
C	1.5186	−1.4364	2.3787
C	2.9502	−1.9103	2.5921
O	3.2420	−3.0967	2.7254
N	3.8464	−0.8978	2.5264
C	5.2801	−1.0491	2.6184
C	5.8772	−0.3953	3.8895
O	7.0643	−0.0616	3.9139
C	5.9475	−0.5320	1.3425
O	5.5023	0.7878	1.0083
N	5.0293	−0.2852	4.9354
C	5.4736	0.1379	6.2585
H	−6.8825	−2.0449	2.9496
H	−5.5227	−0.9214	3.2031
H	−5.2224	−3.9835	3.1959
H	−0.9155	−5.4237	4.7886
H	0.3723	−4.8881	3.6889
H	−1.5193	−6.5778	2.6863
H	−1.0916	−5.1375	1.7421
H	−3.5721	−5.5783	3.4266
H	−3.3866	−4.6616	1.9134
H	−1.1677	−3.0082	4.9364
H	1.4443	−0.3520	2.4655
H	1.2511	−1.7186	1.3636
H	7.0225	−0.4777	1.5036
H	5.7508	−1.2227	0.5156
H	4.6331	0.7353	0.5635
H	5.4730	−2.1246	2.7040
H	6.3095	0.8304	6.1452
H	2.4831	7.6622	−2.5173
H	−4.2401	4.7882	−4.6187
H	−2.5449	4.2512	−2.4693
H	0.2000	7.8449	−2.0757
H	5.8124	−0.7160	6.8597
H	4.6388	0.6277	6.7621
H	4.0640	−0.6144	4.8511
H	0.9205	−2.0789	4.2557
H	−7.2591	−2.8150	0.8196
H	−6.1629	−2.3943	−0.4764
H	3.4860	0.0448	2.4853
H	−5.5202	−3.0329	4.6468
C	−10.7259	−4.8933	2.0642
C	−9.8492	−4.1513	1.0757
O	−8.7557	−3.6932	1.4190
C	−11.2575	−3.8944	3.0981
N	−10.3679	−3.9601	−0.1577
C	−9.6923	−3.1576	−1.1621
C	−8.7627	7.2350	2.9538
C	−8.4587	5.9892	2.1331
C	−8.0520	6.2866	0.6896
N	−7.5828	5.1981	0.0269
O	−8.1616	7.4007	0.1833
H	−10.4234	−3.3835	3.5845
H	−11.8512	−4.4005	3.8644
H	−11.8886	−3.1369	2.6231
H	−11.5503	−5.4111	1.5609
H	−8.7833	−3.6516	−1.5227
H	−7.6688	5.3933	2.5995
H	−9.3371	5.3334	2.0951
H	−7.4712	4.2883	0.4844
H	−7.3012	5.3195	−0.9336
H	−9.5360	7.8365	2.4696
H	−9.1014	6.9635	3.9585
H	−7.8759	7.8684	3.0484
H	−10.1057	−5.6495	2.5542
H	−9.4092	−2.1862	−0.7475
H	−10.3658	−3.0002	−2.0052
H	−11.2638	−4.3660	−0.3749

Int5

C	11.4455	5.0750	−0.4996
C	10.6560	3.9709	−1.2240
C	9.7087	3.2066	−0.3418
C	8.3647	2.9579	−0.5317
N	10.1369	2.5914	0.8218
C	9.0687	1.9863	1.3158
N	7.9701	2.1763	0.5347
H	10.7719	5.8458	−0.1109
H	11.3729	3.2663	−1.6684
H	10.0907	4.4006	−2.0600
H	7.6696	3.2601	−1.3001
H	9.0234	1.4029	2.2258
H	7.0583	1.7329	0.6745
C	−9.4712	2.2452	−4.3869
C	−8.2855	2.8643	−3.6231
C	−7.2888	1.8470	−3.1069
C	−6.3381	1.2659	−3.9593
C	−7.2742	1.4599	−1.7626
C	−5.4194	0.3294	−3.4919
C	−6.3574	0.5278	−1.2723
C	−5.4254	−0.0478	−2.1419
O	−4.5117	−0.9826	−1.7503
H	−10.0289	1.5588	−3.7413
H	−8.6705	3.4481	−2.7787
H	−7.7737	3.5760	−4.2837
H	−6.3051	1.5613	−5.0059
H	−7.9546	1.9073	−1.0441
H	−4.6780	−0.1088	−4.1527
H	−6.3443	0.3198	−0.2060
H	−4.4911	−1.1188	−0.7714
C	−0.4034	−6.7012	−5.1250
C	−0.3104	−5.9060	−3.8144
C	−1.6427	−5.4292	−3.2713
C	−1.9855	−5.6356	−1.9309
C	−2.5726	−4.7530	−4.0779
C	−3.2038	−5.2004	−1.4113
C	−3.7915	−4.3074	−3.5745
C	−4.1189	−4.5385	−2.2343
O	−5.3492	−4.1417	−1.8029
H	−0.8247	−6.0968	−5.9340
H	0.1774	−6.5219	−3.0510
H	0.3544	−5.0443	−3.9681
H	−1.2908	−6.1539	−1.2760
H	−2.3437	−4.5682	−5.1238
H	−3.4478	−5.3857	−0.3700
H	−4.4983	−3.7711	−4.1977
H	−5.3857	−4.1394	−0.8339
C	4.8550	−4.3628	−5.5303
C	5.1310	−5.0790	−4.2293
O	6.2673	−5.3847	−3.8712
C	5.1166	−2.8552	−5.3662
H	5.5241	−4.7761	−6.2897
H	6.1508	−2.6836	−5.0563
H	4.4617	−2.4218	−4.6026
H	4.9449	−2.3214	−6.3051
N	4.0412	−5.2784	−3.4480
C	4.1876	−5.6956	−2.0682

C	2.9093	-5.4568	-1.2721
C	2.4027	-4.0032	-1.2488
C	1.1836	-3.9205	-0.3292
C	3.4951	-3.0166	-0.8138
H	3.1410	-4.9490	-3.7598
H	5.0176	-5.1415	-1.6245
H	3.0988	-5.7725	-0.2392
H	2.1109	-6.1089	-1.6532
H	2.0735	-3.7252	-2.2631
H	1.4762	-4.1188	0.7067
H	0.7246	-2.9339	-0.3563
H	0.4102	-4.6375	-0.6223
H	4.3171	-2.9641	-1.5340
H	3.9041	-3.3108	0.1572
H	3.0921	-2.0056	-0.7001
C	9.1197	-5.5935	-0.8141
C	7.8109	-4.8014	-0.6889
C	7.9687	-3.3208	-0.9655
C	7.2911	-2.7236	-2.0352
C	8.7991	-2.5154	-0.1731
C	7.4293	-1.3596	-2.3008
C	8.9551	-1.1570	-0.4457
C	8.2652	-0.5712	-1.5102
H	9.8820	-5.2253	-0.1198
H	7.3985	-4.9426	0.3193
H	7.0907	-5.2167	-1.3978
H	6.6769	-3.3467	-2.6752
H	9.3275	-2.9558	0.6687
H	6.8863	-0.9186	-3.1323
H	9.6015	-0.5440	0.1739
H	8.3874	0.4888	-1.7056
C	-2.5561	0.6132	0.5716
C	-1.4321	0.9626	-0.1064
C	-1.3876	2.2529	-0.7825
N	-0.2775	2.6546	-1.3075
N	-2.5669	3.0124	-0.8025
C	-3.6097	2.6174	-0.1185
N	-3.6421	1.4508	0.5850
S	1.9474	-0.3942	-3.3198
C	1.5588	0.2590	-1.7891
C	-4.8229	3.4926	-0.0453
N	0.4120	-0.2468	-1.3317
C	-0.2048	-1.1871	-2.1716
C	-1.4921	-1.8333	-1.7874
C	0.5108	-1.3794	-3.3177
C	0.1615	-2.2122	-4.5078
C	-0.1902	0.1302	-0.0188
H	1.7157	2.1089	-0.8901
H	-0.3533	3.5769	-1.7353
H	-0.4081	-0.7992	0.5082
H	0.5924	0.6551	0.5193
H	-5.7425	2.9090	-0.0336
H	-4.7965	4.0791	0.8799
H	-4.8218	4.1870	-0.8856
H	-2.6677	-0.3127	1.1237
H	-1.4875	-2.1614	-0.7455
H	-1.6641	-2.7103	-2.4093
H	-2.3401	-1.1572	-1.9220
H	-0.6506	-2.8969	-4.2636
H	1.0174	-2.7998	-4.8552
C	1.7530	-2.9621	7.4185
C	1.1802	-1.5503	7.3387
C	1.8729	-0.7240	6.2569
O	2.1974	-1.3259	5.1895
O	2.0721	0.5039	6.4858
C	-8.3225	2.7236	4.1024
C	-6.8950	2.4554	3.6098
C	-6.8125	1.8377	2.1982
O	-7.6054	2.2763	1.3236
O	-5.9108	0.9611	2.0089
H	-8.3403	3.4208	4.9465
H	-8.9250	3.1305	3.2914
H	-6.3390	1.8222	4.3064
H	-6.3428	3.4049	3.5490
C	-0.8380	1.8519	5.5122
C	-0.6527	1.3711	4.0723
C	0.6438	1.7977	3.4636
C	0.9391	2.3049	2.2166
N	1.8652	1.6094	4.0801
C	2.8298	1.9623	3.2034
N	2.3100	2.3835	2.0491

H	-0.0425	1.4758	6.1601
H	-0.7188	0.2752	4.0472
H	-1.4646	1.7340	3.4333
H	0.2630	2.6110	1.4322
H	3.8844	1.9025	3.4258
H	2.8327	1.4777	0.7725
C	5.5011	6.9359	3.0592
C	5.7229	5.5967	3.7657
C	6.1837	4.4411	2.8580
C	6.3799	3.1742	3.7036
C	5.2097	4.2096	1.6949
H	4.6883	6.8755	2.3293
H	4.7886	5.2931	4.2590
H	6.4608	5.7285	4.5685
H	7.1600	4.7088	2.4276
H	5.4821	2.9582	4.2952
H	6.5936	2.2945	3.0938
H	7.2053	3.2962	4.4134
H	4.1822	4.0800	2.0459
H	5.4723	3.3099	1.1335
H	5.2189	5.0488	0.9919
H	-0.1698	-1.5783	-5.3377
H	-8.7981	1.7915	4.4288
H	11.9872	4.6490	0.3488
H	12.1647	5.5524	-1.1737
H	-10.1605	3.0183	-4.7430
H	-9.1208	1.6740	-5.2532
H	-0.8257	2.9459	5.5618
H	-1.7975	1.5038	5.9068
H	6.4036	7.2550	2.5240
H	5.2438	7.7230	3.7761
H	0.1107	-1.5871	7.0861
H	1.2595	-1.0188	8.2910
H	1.6935	-3.4530	6.4446
H	1.2213	-3.5736	8.1561
H	2.8104	-2.9376	7.7022
H	8.9546	-6.6549	-0.6015
H	9.5218	-5.5084	-1.8283
H	4.4660	-6.7560	-2.0275
H	0.5874	-7.0394	-5.4453
H	-1.0424	-7.5808	-5.0015
H	3.8242	-4.5356	-5.8639
H	2.0282	1.2006	5.0448
H	-4.5060	1.2034	1.1268
C	2.2073	6.8284	-2.7662
C	0.9049	6.1483	-2.4180
O	0.6743	4.9896	-2.7647
N	0.0039	6.8733	-1.7035
C	-1.3539	6.3685	-1.5739
C	-1.9552	6.0926	-2.9642
O	-1.7322	6.8607	-3.8995
N	-2.7244	4.9863	-3.0276
C	-3.2751	4.5209	-4.2902
C	-2.8798	3.0734	-4.6030
C	-1.3691	2.8006	-4.6749
C	-1.1263	1.3213	-4.9951
C	-0.6467	3.7115	-5.6728
C	3.4710	1.8442	-2.0903
C	3.1234	2.9090	-2.9316
C	4.0616	3.4264	-3.8233
C	5.3502	2.8911	-3.8792
C	5.6967	1.8299	-3.0417
C	4.7598	1.3027	-2.1510
C	2.4301	1.3059	-1.1251
O	2.9962	0.7734	0.0640
H	3.0285	6.1364	-2.5675
H	2.2050	7.0501	-3.8380
H	-1.3666	5.4718	-0.9498
H	-1.9530	7.1336	-1.0733
H	-3.3286	2.4117	-3.8515
H	-3.3467	2.8025	-5.5615
H	-0.9419	2.9919	-3.6876
H	0.4193	3.4624	-5.7126
H	-1.0584	3.5959	-6.6847
H	-0.7248	4.7628	-5.3858
H	-1.5475	1.0544	-5.9735
H	-1.5874	0.6721	-4.2424
H	-0.0542	1.1001	-5.0198
H	-2.9257	5.2114	-5.0594
H	2.1314	3.3471	-2.8791
H	3.7824	4.2549	-4.4676

H	6.0833	3.3006	-4.5681
H	6.6984	1.4155	-3.0737
H	5.0430	0.4867	-1.4970
C	-5.0976	-3.2539	3.5208
C	-3.6275	-2.8644	3.7281
O	-3.3245	-1.7988	4.2573
C	-5.8897	-2.1867	2.7390
C	-5.5325	-2.1131	1.2678
N	-6.4708	-2.5344	0.3858
O	-4.4274	-1.7031	0.8783
N	-2.6822	-3.7927	3.4003
C	-1.2792	-3.5637	3.7172
C	-0.5894	-2.6322	2.7131
O	-1.0327	-2.4861	1.5658
C	-0.6690	-4.9726	3.5639
C	-1.4521	-5.5354	2.3663
C	-2.8755	-4.9845	2.5666
N	0.5735	-2.1230	3.1506
C	1.5271	-1.4683	2.2895
C	2.9296	-2.0344	2.4867
O	3.1555	-3.2413	2.5487
N	3.8774	-1.0689	2.5008
C	5.3018	-1.2804	2.6170
C	5.8816	-0.6874	3.9242
O	7.0769	-0.3937	3.9947
C	6.0228	-0.7231	1.3875
O	5.6270	0.6269	1.1202
N	5.0040	-0.5670	4.9437
C	5.4277	-0.1758	6.2807
H	-6.9595	-2.3846	2.8458
H	-5.6758	-1.1917	3.1409
H	-5.2138	-4.2346	3.0542
H	-0.8669	-5.5556	4.4694
H	0.4105	-4.9344	3.4015
H	-1.4354	-6.6272	2.3166
H	-1.0351	-5.1391	1.4377
H	-3.5184	-5.7158	3.0730
H	-3.3399	-4.7116	1.6151
H	-1.1862	-3.1565	4.7269
H	1.5119	-0.3892	2.4399
H	1.2544	-1.6710	1.2569
H	7.0934	-0.7125	1.5822
H	5.8280	-1.3591	0.5177
H	4.7781	0.6233	0.6367
H	5.4562	-2.3644	2.6614
H	6.2556	0.5309	6.1990
H	2.3728	7.7564	-2.2131
H	-4.3713	4.5881	-4.2636
H	-2.7231	4.3405	-2.2290
H	0.1328	7.8730	-1.6764
H	5.7717	-1.0404	6.8632
H	4.5807	0.2888	6.7878
H	4.0337	-0.8739	4.8352
H	0.8811	-2.1793	4.1242
H	-7.3928	-2.8496	0.6824
H	-6.3240	-2.3123	-0.5910
H	3.5593	-0.1113	2.5158
H	-5.5248	-3.3205	4.5269
C	-10.7829	-5.1065	1.8290
C	-9.8974	-4.3241	0.8820
O	-8.8064	-3.8842	1.2543
C	-11.2776	-4.1566	2.9244
N	-10.4008	-4.0791	-0.3480
C	-9.7125	-3.2229	-1.2971
C	-8.8450	6.9819	3.2769
C	-8.5976	5.7282	2.4491
C	-8.2549	6.0109	0.9862
N	-7.9232	4.8987	0.2812
O	-8.2900	7.1405	0.5031
H	-10.4254	-3.6960	3.4290
H	-11.8772	-4.6901	3.6674
H	-11.8933	-3.3568	2.5012
H	-11.6238	-5.5725	1.3030
H	-8.7509	-3.6563	-1.5909
H	-7.7884	5.1317	2.8807
H	-9.4805	5.0783	2.4635
H	-7.8469	3.9696	0.7102
H	-7.6558	5.0226	-0.6832
H	-9.6482	7.5821	2.8419
H	-9.1171	6.7190	4.3040
H	-7.9531	7.6142	3.3060

```
H -10.1779  -5.9059   2.2670
H  -9.5236  -2.2346  -0.8668
H -10.3312  -3.1083  -2.1876
H -11.3091  -4.4453  -0.5835
```

Int6

```
C  11.2752   5.2542  -0.3285
C  10.5369   4.1137  -1.0525
C   9.5673   3.3501  -0.1931
C   8.2412   3.0560  -0.4420
N   9.9549   2.7760   1.0054
C   8.8797   2.1527   1.4614
N   7.8159   2.2905   0.6233
H  10.5718   6.0217   0.0108
H  11.2870   3.4139  -1.4473
H   9.9999   4.5092  -1.9234
H   7.5774   3.3149  -1.2525
H   8.8040   1.5914   2.3831
H   6.9107   1.8202   0.7379
C  -9.5334   2.1786  -4.5915
C  -8.3589   2.8231  -3.8314
C  -7.3582   1.8215  -3.2931
C  -6.4131   1.2191  -4.1364
C  -7.3408   1.4644  -1.9405
C  -5.4993   0.2861  -3.6524
C  -6.4296   0.5362  -1.4337
C  -5.5097  -0.0642  -2.2965
O  -4.6123  -1.0190  -1.8950
H -10.0883   1.4980  -3.9375
H  -8.7538   3.4175  -2.9993
H  -7.8489   3.5277  -4.5009
H  -6.3819   1.4928  -5.1887
H  -8.0254   1.9232  -1.2338
H  -4.7634  -0.1717  -4.3059
H  -6.4265   0.3301  -0.3674
H  -4.5372  -1.0949  -0.9204
C  -0.2922  -6.5717  -5.5066
C   0.0643  -6.0581  -4.1087
C  -1.1017  -5.5864  -3.2565
C  -0.8667  -5.1327  -1.9490
C  -2.4239  -5.5708  -3.7138
C  -1.8926  -4.6768  -1.1287
C  -3.4670  -5.1194  -2.9037
C  -3.2093  -4.6620  -1.6101
O  -4.2588  -4.2392  -0.8531
H  -0.7721  -5.7931  -6.1080
H   0.6060  -6.8445  -3.5664
H   0.7808  -5.2291  -4.2100
H   0.1461  -5.1319  -1.5597
H  -2.6558  -5.9069  -4.7188
H  -1.6731  -4.2840  -0.1401
H  -4.4881  -5.0936  -3.2694
H  -4.0069  -3.4849  -0.2730
C   4.9269  -4.1259  -5.7622
C   5.2228  -4.8373  -4.4652
O   6.3696  -5.0616  -4.0812
C   4.7612  -2.6147  -5.5166
H   5.7645  -4.3092  -6.4388
H   5.6555  -2.2017  -5.0417
H   3.9159  -2.4114  -4.8511
H   4.5933  -2.0794  -6.4552
N   4.1245  -5.1311  -3.7241
C   4.2379  -5.5873  -2.3562
C   3.1305  -5.0422  -1.4622
C   3.0068  -3.5110  -1.4433
C   2.0215  -3.0763  -0.3586
C   4.3582  -2.8210  -1.2404
H   3.2094  -4.9191  -4.0920
H   5.2151  -5.2694  -1.9956
H   3.3319  -5.3958  -0.4428
H   2.1701  -5.4883  -1.7515
H   2.6059  -3.1846  -2.4128
H   2.3221  -3.4816   0.6138
H   2.0234  -1.9892  -0.2718
H   0.9982  -3.4036  -0.5523
H   5.0418  -2.9715  -2.0783
H   4.8528  -3.2005  -0.3389
H   4.2115  -1.7464  -1.1090
C   9.1538  -5.4380  -1.0324
C   7.8772  -4.5833  -1.0338
C   8.1387  -3.1074  -1.2465
C   7.8315  -2.4980  -2.4701
C   8.7017  -2.3202  -0.2326
C   8.0611  -1.1339  -2.6636
C   8.9459  -0.9612  -0.4282
C   8.6162  -0.3587  -1.6446
H   9.8453  -5.1204  -0.2447
H   7.3493  -4.7254  -0.0812
H   7.2231  -4.9430  -1.8308
H   7.4144  -3.1064  -3.2674
H   8.9419  -2.7760   0.7253
H   7.8071  -0.6771  -3.6168
H   9.3797  -0.3610   0.3646
H   8.7940   0.7029  -1.7824
C  -2.5827   0.5985   0.5583
C  -1.4293   0.9750  -0.0492
C  -1.3648   2.2798  -0.7014
N  -0.2312   2.7035  -1.1492
N  -2.5600   3.0134  -0.7953
C  -3.6295   2.5944  -0.1692
N  -3.6805   1.4227   0.5253
S   2.0328  -0.3551  -3.2257
C   1.6039   0.2970  -1.7008
C  -4.8614   3.4478  -0.1542
N   0.4201  -0.1719  -1.3058
C  -0.2045  -1.0544  -2.1995
C  -1.5742  -1.5657  -1.9330
C   0.5614  -1.2901  -3.3002
C   0.2334  -2.1358  -4.4902
C  -0.1883   0.1383   0.0208
H   1.8883   2.0139  -0.5680
H  -0.3066   3.6197  -1.5922
H  -0.4050  -0.8230   0.4908
H   0.5914   0.6343   0.5921
H  -5.7699   2.8505  -0.0911
H  -4.8380   4.1057   0.7220
H  -4.8811   4.0785  -1.0436
H  -2.7195  -0.3429   1.0807
H  -1.6681  -1.9598  -0.9199
H  -1.8161  -2.3677  -2.6270
H  -2.3225  -0.7810  -2.0574
H  -0.4358  -2.9514  -4.2061
H   1.1370  -2.5745  -4.9243
C   1.6327  -3.2223   7.1837
C   0.7837  -1.9930   7.5401
C   1.4005  -0.7034   7.0289
O   1.3108  -0.5992   5.6971
O   1.9244   0.1318   7.7418
C  -8.5039   2.3903   3.9241
C  -7.0408   2.1851   3.5129
C  -6.8513   1.6562   2.0774
O  -7.5910   2.1337   1.1778
O  -5.9196   0.8078   1.8972
H  -8.5945   3.0376   4.8024
H  -9.0636   2.8287   3.0993
H  -6.5133   1.5256   4.2072
H  -6.5128   3.1496   3.5414
C  -1.0230   1.6086   5.4036
C  -0.5877   2.6122   4.3338
C   0.7052   2.2368   3.6794
C   1.0787   2.3777   2.3627
N   1.7589   1.6709   4.3886
C   2.7378   1.4931   3.5084
N   2.3782   1.9090   2.2746
H  -1.1186   0.6018   4.9867
H  -1.3578   2.7002   3.5588
H  -0.4955   3.6071   4.7914
H   0.5612   2.7785   1.5033
H   3.6842   1.0271   3.7221
H   2.8379   1.5643   1.4038
C   5.2516   6.8855   3.2102
C   5.4279   5.5200   3.8756
C   5.8271   4.3753   2.9272
C   5.9771   3.0757   3.7273
C   4.8255   4.2129   1.7763
H   4.4231   6.8819   2.4954
H   4.4910   5.2397   4.3778
H   6.1845   5.5985   4.6677
H   6.8055   4.6176   2.4866
H   5.0548   2.8604   4.2789
H   6.1842   2.2179   3.0863
H   6.7873   3.1539   4.4607
H   3.8076   4.0857   2.1585
H   5.0621   3.3313   1.1753
H   4.8303   5.0798   1.1081
H  -0.2587  -1.5439  -5.2701
H  -8.9741   1.4303   4.1661
H  11.7904   4.8632   0.5527
H  12.0115   5.7267  -0.9874
H -10.2281   2.9383  -4.9653
H  -9.1720   1.5963  -5.4458
H  -0.2981   1.5734   6.2200
H  -1.9933   1.8915   5.8236
H   6.1567   7.1793   2.6649
H   5.0431   7.6643   3.9519
H  -0.2175  -2.0894   7.1035
H   0.6767  -1.8969   8.6229
H   1.8031  -3.2911   6.1050
H   1.1432  -4.1405   7.5223
H   2.6129  -3.1649   7.6675
H   8.9177  -6.4955  -0.8728
H   9.6751  -5.3464  -1.9903
H   4.2342  -6.6860  -2.3188
H   0.6082  -6.8965  -6.0366
H  -0.9765  -7.4241  -5.4543
H   4.0211  -4.5325  -6.2294
H   1.5705   0.3366   5.3746
H  -4.5631   1.1567   1.0329
C   2.0359   6.9153  -2.6597
C   0.7430   6.2037  -2.3443
O   0.5513   5.0411  -2.7003
N  -0.1881   6.9046  -1.6413
C  -1.5373   6.3706  -1.5400
C  -2.1245   6.1287  -2.9416
O  -1.9097   6.9278  -3.8519
N  -2.8764   5.0115  -3.0446
C  -3.3824   4.5617  -4.3346
C  -2.9924   3.1079  -4.6208
C  -1.4851   2.8122  -4.5651
C  -1.2354   1.3272  -4.8487
C  -0.6686   3.6995  -5.5106
C   3.5349   1.8461  -1.8997
C   3.1306   2.9530  -2.6594
C   4.0150   3.5397  -3.5628
C   5.3075   3.0298  -3.7171
C   5.7093   1.9247  -2.9660
C   4.8255   1.3337  -2.0606
C   2.5519   1.2076  -0.9190
O   3.1477   0.4981   0.1083
H   2.8686   6.2554  -2.4062
H   2.0730   7.1026  -3.7372
H  -1.5387   5.4542  -0.9456
H  -2.1546   7.1082  -1.0202
H  -3.5113   2.4529  -3.9094
H  -3.3833   2.8447  -5.6145
H  -1.1354   3.0104  -3.5481
H   0.3941   3.4430  -5.4492
H  -0.9912   3.5661  -6.5522
H  -0.7606   4.7569  -5.2523
H  -1.5518   1.0602  -5.8659
H  -1.7874   0.6901  -4.1492
H  -0.1716   1.0895  -4.7534
H  -2.9832   5.2456  -5.0853
H   2.1293   3.3574  -2.5456
H   3.6889   4.3966  -4.1451
H   5.9969   3.4909  -4.4188
H   6.7105   1.5200  -3.0730
H   5.1458   0.4848  -1.4698
C  -5.1611  -3.5049   3.1854
C  -3.7051  -3.1352   3.4679
O  -3.4134  -2.0644   3.9959
C  -5.9189  -2.3380   2.5182
C  -5.5435  -2.2242   1.0615
N  -6.5069  -2.4062   0.1577
O  -4.3504  -2.0231   0.7090
N  -2.7483  -4.0705   3.2060
C  -1.3846  -3.8231   3.6491
C  -0.6187  -2.8808   2.7150
O  -0.8230  -2.8590   1.4944
C  -0.7487  -5.2280   3.6080
C  -1.4657  -5.8972   2.4253
C  -2.9031  -5.3530   2.5061
```

```
N   0.3558  -2.1805   3.3265
C   1.3533  -1.4328   2.6101
C   2.7733  -1.8809   2.9636
O   3.0019  -2.7150   3.8499
N   3.7237  -1.2247   2.2721
C   5.1673  -1.3418   2.4800
C   5.7011  -0.7478   3.8150
O   6.8305  -0.2662   3.8718
C   5.9028  -0.6668   1.3155
O   5.5200   0.6933   1.1577
N   4.8633  -0.8117   4.8794
C   5.2688  -0.3314   6.1895
H  -6.9971  -2.4837   2.6138
H  -5.6438  -1.3931   2.9949
H  -5.2580  -4.4181   2.5936
H  -0.9735  -5.7496   4.5444
H   0.3371  -5.1841   3.4957
H  -1.4382  -6.9889   2.4696
H  -1.0040  -5.5837   1.4875
H  -3.5495  -6.0309   3.0794
H  -3.3468  -5.2126   1.5163
H  -1.4003  -3.3929   4.6541
H   1.2582  -0.3664   2.8247
H   1.1827  -1.5741   1.5486
H   6.9669  -0.6907   1.5385
H   5.7308  -1.2280   0.3947
H   4.6707   0.7233   0.6290
H   5.4367  -2.4077   2.4930
H   5.8971   0.5506   6.0598
H   2.1513   7.8645  -2.1303
H  -4.4771   4.6498  -4.3597
H  -2.8298   4.3346  -2.2753
H  -0.0886   7.9081  -1.6256
H   5.8480  -1.0832   6.7415
H   4.3792  -0.0661   6.7651
H   4.0759  -1.4503   4.8208
H   0.4248  -2.1434   4.3354
H  -7.4716  -2.6028   0.4215
H  -6.2440  -2.3875  -0.8188
H   3.4088  -0.6234   1.5046
H  -5.6168  -3.7005   4.1628
C -10.3322  -5.2490   1.5713
C  -9.7283  -4.2490   0.6084
O  -9.0164  -3.3272   1.0158
C -11.3147  -4.4987   2.4755
N -10.0956  -4.3682  -0.6875
C  -9.7129  -3.3944  -1.6908
C  -9.0944   6.6637   3.2348
C  -8.8026   5.4575   2.3528
C  -8.4245   5.8207   0.9164
N  -8.0112   4.7576   0.1795
O  -8.5087   6.9652   0.4770
H -10.8003  -3.6779   2.9793
H -11.7413  -5.1630   3.2325
H -12.1381  -4.0762   1.8912
H -10.8304  -6.0705   1.0443
H  -8.7812  -3.6698  -2.1988
H  -7.9981   4.8499   2.7769
H  -9.6764   4.7966   2.3066
H  -7.8936   3.8207   0.5795
H  -7.7227   4.9338  -0.7706
H  -9.8877   7.2777   2.8012
H  -9.4002   6.3456   4.2364
H  -8.2113   7.3020   3.3297
H  -9.5188  -5.6757   2.1657
H  -9.5660  -2.4296  -1.2030
H -10.5057  -3.3017  -2.4360
H -10.6488  -5.1648  -0.9611
```

TS7

```
C  11.4906   5.3100  -0.3759
C  10.7457   4.1495  -1.0687
C   9.6876   3.4607  -0.2447
C   8.3867   3.1563  -0.5970
N   9.9583   2.9488   1.0125
C   8.8410   2.3544   1.4038
N   7.8570   2.4527   0.4663
H  10.8048   6.1292  -0.1359
H  11.4916   3.4004  -1.3703
H  10.2848   4.5081  -1.9975
H   7.8020   3.3727  -1.4786
H   8.6810   1.8318   2.3381
H   6.9424   1.9898   0.5228
C  -9.3660   2.3394  -4.4761
C  -8.3466   2.9543  -3.5009
C  -7.3246   1.9494  -3.0194
C  -6.3091   1.4971  -3.8749
C  -7.3646   1.4208  -1.7245
C  -5.3857   0.5416  -3.4637
C  -6.4461   0.4633  -1.2909
C  -5.4536   0.0097  -2.1688
O  -4.5393  -0.9414  -1.8402
H  -9.9140   1.5222  -3.9956
H  -8.8742   3.3802  -2.6397
H  -7.8383   3.7904  -3.9987
H  -6.2351   1.9065  -4.8798
H  -8.1054   1.7887  -1.0192
H  -4.5960   0.2003  -4.1250
H  -6.4653   0.1323  -0.2591
H  -4.5204  -1.1501  -0.8692
C  -0.1917  -6.4838  -5.3658
C  -0.3243  -6.2632  -3.8552
C  -1.6691  -5.7524  -3.3677
C  -2.0089  -5.8791  -2.0135
C  -2.5979  -5.1209  -4.2058
C  -3.2159  -5.4010  -1.5109
C  -3.8146  -4.6416  -3.7211
C  -4.1331  -4.7862  -2.3696
O  -5.3577  -4.3502  -1.9500
H  -0.2827  -5.5446  -5.9213
H  -0.1029  -7.2030  -3.3350
H   0.4595  -5.5648  -3.5297
H  -1.3166  -6.3675  -1.3341
H  -2.3792  -5.0015  -5.2620
H  -3.4474  -5.5078  -0.4540
H  -4.5264  -4.1521  -4.3767
H  -5.4030  -4.3688  -0.9820
C   5.0421  -4.0774  -5.6778
C   5.2928  -4.8586  -4.4096
O   6.3942  -5.3201  -4.1182
C   5.5418  -2.6316  -5.5116
H   5.5904  -4.5693  -6.4861
H   6.6016  -2.6316  -5.2442
H   4.9948  -2.1140  -4.7162
H   5.4159  -2.0610  -6.4360
N   4.2187  -4.9569  -3.5868
C   4.3667  -5.5008  -2.2539
C   3.0400  -5.5271  -1.5128
C   2.3987  -4.1455  -1.3075
C   1.1004  -4.3163  -0.5174
C   3.3810  -3.1663  -0.6546
H   3.3831  -4.4373  -3.8049
H   5.1078  -4.9199  -1.6940
H   3.2160  -5.9761  -0.5276
H   2.3376  -6.1887  -2.0380
H   2.1196  -3.7335  -2.2904
H   1.2995  -4.7601   0.4648
H   0.5836  -3.3740  -0.3453
H   0.4063  -4.9762  -1.0455
H   4.2688  -3.0132  -1.2752
H   3.7210  -3.5522   0.3133
H   2.9564  -2.1743  -0.4921
C   9.2890  -5.3734  -0.9631
C   8.1272  -4.5542  -0.3740
C   8.1519  -3.1069  -0.8139
C   7.4503  -2.6926  -1.9538
C   8.9097  -2.1587  -0.1141
C   7.4920  -1.3600  -2.3683
C   8.9640  -0.8289  -0.5324
C   8.2463  -0.4240  -1.6586
H  10.2542  -4.9386  -0.6830
H   8.1630  -4.6030   0.7208
H   7.1812  -5.0144  -0.6822
H   6.8936  -3.4277  -2.5271
H   9.4514  -2.4641   0.7777
H   6.9320  -1.0543  -3.2473
H   9.5424  -0.1012   0.0281
H   8.2744   0.6168  -1.9628
C  -2.5720   0.6474   0.4343
C  -1.3369   0.9788  -0.0522
C  -1.1709   2.3352  -0.5398
N   0.0199   2.8926  -0.6259
N  -2.3071   3.0519  -0.8613
C  -3.4768   2.6451  -0.4121
N  -3.6237   1.5093   0.3214
S   1.9439  -0.7280  -3.1958
C   1.5711   0.0227  -1.7021
C  -4.7034   3.4442  -0.7051
N   0.3970  -0.4155  -1.2352
C  -0.2436  -1.3914  -2.0273
C  -1.5509  -1.9793  -1.6142
C   0.4746  -1.6733  -3.1492
C   0.1260  -2.5878  -4.2787
C  -0.1738   0.0283   0.0704
H   2.0183   1.6995  -0.5137
H   0.0060   3.8157  -1.0621
H  -0.4729  -0.8736   0.6075
H   0.6566   0.4698   0.6074
H  -5.3994   3.3995   0.1403
H  -4.4312   4.4765  -0.9254
H  -5.2093   3.0202  -1.5784
H  -2.7878  -0.2882   0.9400
H  -1.5449  -2.2887  -0.5656
H  -1.7653  -2.8635  -2.2143
H  -2.3711  -1.2744  -1.7664
H  -0.7232  -3.2184  -4.0149
H   0.9638  -3.2434  -4.5393
C   1.8375  -3.0244   7.2788
C   1.0918  -1.6884   7.1224
C   1.8769  -0.6216   6.3305
O   2.1005  -0.9116   5.0824
O   2.2257   0.4217   6.8927
C  -8.2798   2.6258   4.0300
C  -7.3823   1.5168   3.4667
C  -6.5393   1.8961   2.2338
O  -6.6380   3.0581   1.7684
O  -5.7788   0.9746   1.7857
H  -8.8250   2.2702   4.9116
H  -7.6887   3.4966   4.3214
H  -7.9781   0.6367   3.1913
H  -6.6780   1.1609   4.2285
C  -0.7954   1.8048   5.4707
C  -0.6240   0.7658   4.3701
C   0.2629   1.3412   3.3285
C  -0.0211   2.2578   2.3591
N   1.6217   1.1008   3.2359
C   2.1177   1.8111   2.2018
N   1.1308   2.5262   1.6405
H   0.1670   2.0094   5.9480
H  -0.1722  -0.1410   4.7690
H  -1.5863   0.4860   3.9294
H  -0.9636   2.7121   2.1061
H   3.1367   1.7600   1.8581
H   0.8129   2.7812   0.4623
C   5.5014   7.0173   3.1851
C   5.2149   5.6249   3.7595
C   5.4978   4.4435   2.8118
C   5.3360   3.1114   3.5568
C   4.6101   4.4710   1.5595
H   4.8566   7.2448   2.3299
H   4.1650   5.5708   4.0816
H   5.8155   5.4865   4.6679
H   6.5439   4.5179   2.4799
H   4.3179   3.0065   3.9526
H   5.5320   2.2629   2.8975
H   6.0247   3.0394   4.4047
H   3.5486   4.4234   1.8325
H   4.8286   3.6145   0.9134
H   4.7616   5.3781   0.9655
H  -0.1417  -2.0180  -5.1754
H  -9.0073   2.9543   3.2857
H  11.9341   4.9623   0.5608
H  12.2855   5.7007  -1.0197
H -10.0916   3.0858  -4.8163
H  -8.8629   1.9271  -5.3568
H  -1.1971   2.7395   5.0681
H  -1.4824   1.4484   6.2461
H   6.5404   7.0940   2.8441
H   5.3377   7.7963   3.9375
H   0.1435  -1.8682   6.5969
H   0.8489  -1.2670   8.1016
H   2.1507  -3.4039   6.3011
```

H	1.2092	-3.7777	7.7657
H	2.7406	-2.8932	7.8838
H	9.2615	-6.4083	-0.6057
H	9.2276	-5.3872	-2.0557
H	4.7774	-6.5124	-2.3411
H	0.7870	-6.9109	-5.6026
H	-0.9621	-7.1669	-5.7374
H	3.9779	-4.0856	-5.9431
H	2.1015	0.4509	3.9008
H	-4.5483	1.3106	0.8478
C	2.2482	7.0135	-2.6642
C	0.9637	6.3539	-2.2191
O	0.7915	5.1380	-2.3156
N	0.0014	7.1737	-1.7094
C	-1.3508	6.6535	-1.5918
C	-1.9168	6.3221	-2.9833
O	-1.6671	7.0500	-3.9423
N	-2.6996	5.2209	-3.0203
C	-3.1971	4.6818	-4.2816
C	-2.8215	3.2018	-4.4250
C	-1.3269	2.8910	-4.2397
C	-1.1001	1.3767	-4.2684
C	-0.4373	3.6142	-5.2561
C	3.4912	1.5813	-2.0159
C	3.0378	2.7303	-2.6770
C	3.8508	3.3641	-3.6145
C	5.1224	2.8571	-3.8979
C	5.5758	1.7143	-3.2374
C	4.7631	1.0761	-2.2973
C	2.6040	0.8961	-0.9735
O	3.2566	0.0863	-0.0909
H	3.0839	6.3551	-2.4212
H	2.2154	7.1340	-3.7522
H	-1.3537	5.7782	-0.9395
H	-1.9757	7.4218	-1.1285
H	-3.3955	2.6194	-3.6940
H	-3.1524	2.8577	-5.4153
H	-1.0281	3.2445	-3.2487
H	0.6151	3.3649	-5.0853
H	-0.6932	3.3241	-6.2837
H	-0.5336	4.7000	-5.1718
H	-1.3872	0.9490	-5.2380
H	-1.6903	0.8732	-3.4951
H	-0.0460	1.1409	-4.0996
H	-2.7696	5.2928	-5.0788
H	2.0615	3.1450	-2.4485
H	3.4895	4.2576	-4.1155
H	5.7590	3.3540	-4.6246
H	6.5658	1.3223	-3.4487
H	5.1125	0.2017	-1.7611
C	-4.9833	-3.2992	3.3276
C	-3.5953	-2.7039	3.6067
O	-3.4458	-1.5003	3.8056
C	-5.8746	-2.3214	2.5424
C	-5.5050	-2.2167	1.0779
N	-6.4134	-2.6695	0.1832
O	-4.4232	-1.7381	0.7001
N	-2.5686	-3.5879	3.7188
C	-1.2319	-3.1429	4.0883
C	-0.4318	-2.6802	2.8551
O	-0.8853	-2.7983	1.7064
C	-0.6083	-4.4268	4.6661
C	-1.1672	-5.4993	3.7183
C	-2.6045	-5.0259	3.4264
N	0.8043	-2.2514	3.1404
C	1.7752	-1.9976	2.0998
C	3.1972	-2.1871	2.6260
O	3.5534	-3.2015	3.2181
N	3.9893	-1.1199	2.3493
C	5.4311	-1.1250	2.5188
C	5.9410	-0.5111	3.8450
O	7.1175	-0.1455	3.9304
C	6.0986	-0.4791	1.2987
O	5.5455	0.7965	0.9714
N	5.0725	-0.5039	4.8767
C	5.4877	-0.1703	6.2340
H	-6.9216	-2.6218	2.6352
H	-5.7487	-1.3102	2.9423
H	-4.9210	-4.2617	2.8138
H	-0.9684	-4.5798	5.6888
H	0.4815	-4.3913	4.6804
H	-1.1418	-6.5073	4.1393
H	-0.5876	-5.5037	2.7909
H	-3.3318	-5.5334	4.0721
H	-2.8834	-5.2091	2.3829
H	-1.3000	-2.3263	4.8097
H	1.6706	-1.0037	1.6681
H	1.6135	-2.7154	1.3016
H	7.1526	-0.3405	1.5320
H	6.0145	-1.1553	0.4407
H	4.7066	0.6304	0.4447
H	5.7542	-2.1738	2.5660
H	6.3141	0.5406	6.1839
H	2.4045	7.9937	-2.2072
H	-4.2885	4.7886	-4.3311
H	-2.6164	4.5671	-2.2397
H	0.0825	8.1549	-1.9332
H	5.8324	-1.0621	6.7740
H	4.6361	0.2662	6.7583
H	4.1067	-0.7982	4.7470
H	1.1191	-1.9887	4.0895
H	-7.3085	-3.0736	0.4580
H	-6.2310	-2.4955	-0.7975
H	3.6187	-0.4743	1.6514
H	-5.4452	-3.4921	4.3034
C	-10.7837	-5.1757	1.4957
C	-9.8363	-4.4297	0.5809
O	-8.6928	-4.1454	0.9513
C	-11.1480	-4.2568	2.6664
N	-10.3334	-4.0356	-0.6117
C	-9.5655	-3.2040	-1.5206
C	-8.8453	6.8966	3.3035
C	-8.7190	5.6619	2.4180
C	-8.6086	5.9750	0.9273
N	-8.1174	4.9362	0.1904
O	-8.9735	7.0393	0.4352
H	-10.2403	-3.9296	3.1783
H	-11.7884	-4.7726	3.3871
H	-11.6797	-3.3667	2.3157
H	-11.6814	-5.5088	0.9629
H	-8.6474	-3.7124	-1.8325
H	-7.8659	5.0424	2.7034
H	-9.6036	5.0232	2.5417
H	-7.6268	4.1695	0.6528
H	-7.9285	5.1225	-0.7832
H	-9.6716	7.5284	2.9678
H	-9.0173	6.6093	4.3455
H	-7.9356	7.5039	3.2638
H	-10.2620	-6.0649	1.8615
H	-9.2869	-2.2561	-1.0495
H	-10.1671	-2.9935	-2.4052
H	-11.2813	-4.2803	-0.8490

Int7

C	11.3668	5.2960	-0.3214
C	10.5605	4.2092	-1.0575
C	9.6358	3.3989	-0.1884
C	8.3082	3.0854	-0.4046
N	10.0781	2.7779	0.9663
C	9.0333	2.1080	1.4291
N	7.9372	2.2594	0.6366
H	10.7027	6.0549	0.1054
H	11.2720	3.5270	-1.5446
H	9.9737	4.6631	-1.8657
H	7.6094	3.3696	-1.1764
H	9.0043	1.5018	2.3248
H	7.0467	1.7596	0.7555
C	-9.4672	2.2371	-4.4714
C	-8.4175	2.8705	-3.5395
C	-7.3674	1.8935	-3.0538
C	-6.3331	1.4653	-3.9010
C	-7.3832	1.3892	-1.7493
C	-5.3667	0.5616	-3.4696
C	-6.4176	0.4899	-1.2945
C	-5.4021	0.0626	-2.1586
O	-4.4280	-0.8127	-1.7984
H	-10.0031	1.4319	-3.9582
H	-8.9241	3.3141	-2.6743
H	-7.9301	3.6986	-4.0706
H	-6.2775	1.8532	-4.9159
H	-8.1240	1.7361	-1.0356
H	-4.5662	0.2383	-4.1271
H	-6.4235	0.1925	-0.2524
H	-4.4859	-1.0858	-0.8455
C	-0.2362	-6.5175	-5.4482
C	-0.1324	-5.9259	-4.0360
C	-1.4400	-5.4305	-3.4504
C	-1.7784	-5.7182	-2.1235
C	-2.3413	-4.6489	-4.1904
C	-2.9604	-5.2532	-1.5505
C	-3.5280	-4.1767	-3.6356
C	-3.8458	-4.4849	-2.3100
O	-5.0422	-4.0436	-1.8227
H	-0.5728	-5.7726	-6.1758
H	0.2886	-6.6776	-3.3597
H	0.5976	-5.1046	-4.0483
H	-1.1056	-6.3225	-1.5224
H	-2.1159	-4.3997	-5.2233
H	-3.1965	-5.4933	-0.5171
H	-4.2134	-3.5602	-4.2063
H	-5.0733	-4.1609	-0.8625
C	4.9827	-4.0744	-5.7299
C	5.2239	-4.8771	-4.4728
O	6.3264	-5.3384	-4.1838
C	5.3977	-2.6080	-5.5167
H	5.5831	-4.5189	-6.5281
H	6.4529	-2.5506	-5.2381
H	4.8145	-2.1444	-4.7136
H	5.2481	-2.0211	-6.4274
N	4.1473	-4.9950	-3.6548
C	4.3117	-5.5391	-2.3217
C	3.0176	-5.5032	-1.5223
C	2.4137	-4.1040	-1.3069
C	1.2096	-4.2273	-0.3701
C	3.4492	-3.1056	-0.7742
H	3.3051	-4.4831	-3.8670
H	5.0921	-4.9819	-1.7955
H	3.2295	-5.9411	-0.5392
H	2.2700	-6.1525	-1.9979
H	2.0392	-3.7295	-2.2740
H	1.5429	-4.4532	0.6492
H	0.6224	-3.3112	-0.3279
H	0.5331	-5.0235	-0.6956
H	4.2665	-2.9433	-1.4837
H	3.8839	-3.4771	0.1599
H	3.0223	-2.1224	-0.5628
C	9.2353	-5.3942	-1.0253
C	7.8968	-4.6545	-0.9074
C	8.0149	-3.1649	-1.1470
C	7.3440	-2.5627	-2.2174
C	8.8058	-2.3571	-0.3182
C	7.4511	-1.1900	-2.4492
C	8.9266	-0.9886	-0.5530
C	8.2445	-0.3982	-1.6191
H	9.9691	-5.0147	-0.3070
H	7.4712	-4.8324	0.0893
H	7.2020	-5.0780	-1.6374
H	6.7544	-3.1868	-2.8798
H	9.3249	-2.8023	0.5267
H	6.9125	-0.7430	-3.2801
H	9.5353	-0.3718	0.1002
H	8.3351	0.6705	-1.7807
C	-2.5715	0.6588	0.6199
C	-1.3723	1.0449	0.0754
C	-1.3508	2.3283	-0.5564
N	-0.2086	2.8964	-0.9374
N	-2.5007	3.0078	-0.7921
C	-3.6303	2.5555	-0.2670
N	-3.6817	1.4343	0.4818
S	2.0945	-0.3217	-3.0223
C	1.6718	0.3345	-1.4977
C	-4.8843	3.3281	-0.5141
N	0.4994	-0.1635	-1.0788
C	-0.0922	-1.1171	-1.9358
C	-1.3935	-1.7637	-1.5981
C	0.6596	-1.3154	-3.0528
C	0.3531	-2.1612	-4.2454
C	-0.1266	0.2090	0.2191
H	2.2125	1.9208	-0.1060
H	-0.1844	3.8115	-1.3915
H	-0.3715	-0.7182	0.7388
H	0.6305	0.7342	0.7966

H	−5.7122	2.9774	0.1016
H	−4.7010	4.3906	−0.3378
H	−5.1714	3.2038	−1.5636
H	−2.7083	−0.2642	1.1746
H	−1.4272	−2.0928	−0.5571
H	−1.5337	−2.6447	−2.2235
H	−2.2387	−1.0960	−1.7837
H	−0.4394	−2.8729	−4.0151
H	1.2337	−2.7215	−4.5762
C	1.7609	−3.1818	7.2347
C	0.9534	−1.8820	7.1043
C	1.7189	−0.7875	6.3389
O	1.9199	−1.0279	5.0800
O	2.0955	0.2267	6.9402
C	−8.3896	2.4386	4.0383
C	−6.9097	2.2054	3.7152
C	−6.6184	1.7988	2.2620
O	−7.3179	2.2951	1.3453
O	−5.6222	1.0165	2.0908
H	−8.5168	3.0447	4.9406
H	−8.8816	2.9354	3.2044
H	−6.4545	1.4707	4.3843
H	−6.3482	3.1391	3.8646
C	−0.9012	1.6497	5.4764
C	−0.8551	1.1230	4.0399
C	0.3108	1.6778	3.2947
C	0.4113	2.6783	2.3531
N	1.6056	1.2435	3.4972
C	2.4134	1.9584	2.6668
N	1.7245	2.8396	1.9483
H	0.0094	1.3880	6.0228
H	−0.8139	0.0286	4.0446
H	−1.7745	1.3946	3.5090
H	−0.3855	3.2896	1.9526
H	3.4778	1.7950	2.6060
H	0.6611	2.5903	−0.5226
C	5.3640	6.9267	3.2526
C	5.3131	5.5130	3.8398
C	5.8111	4.3894	2.9108
C	5.8230	3.0572	3.6748
C	4.9786	4.2937	1.6232
H	4.7046	7.0263	2.3845
H	4.2813	5.2807	4.1383
H	5.9081	5.4899	4.7628
H	6.8478	4.6188	2.6219
H	4.8333	2.8374	4.0910
H	6.1038	2.2196	3.0324
H	6.5268	3.0898	4.5138
H	3.9126	4.1638	1.8382
H	5.2977	3.4424	1.0147
H	5.0884	5.1924	1.0063
H	0.0182	−1.5428	−5.0861
H	−8.9048	1.4852	4.2017
H	11.9299	4.8480	0.5014
H	12.0679	5.7918	−1.0012
H	−10.2000	2.9795	−4.8044
H	−8.9919	1.8053	−5.3584
H	−0.9941	2.7407	5.4802
H	−1.7576	1.2307	6.0156
H	6.3796	7.1819	2.9268
H	5.0529	7.6736	3.9913
H	0.0164	−2.0857	6.5680
H	0.6937	−1.4890	8.0910
H	2.0556	−3.5480	6.2461
H	1.1849	−3.9642	7.7411
H	2.6767	−3.0096	7.8096
H	9.1074	−6.4662	−0.8414
H	9.6544	−5.2691	−2.0285
H	4.6770	−6.5682	−2.4093
H	0.7378	−6.8878	−5.7826
H	−0.9468	−7.3494	−5.4724
H	3.9296	−4.1304	−6.0328
H	1.8767	0.4833	4.1588
H	−4.5992	1.1989	1.0661
C	2.1156	6.9651	−2.5991
C	0.8139	6.3207	−2.1937
O	0.6276	5.1079	−2.3444
N	−0.1426	7.1211	−1.6616
C	−1.4977	6.6076	−1.5455
C	−2.0477	6.2556	−2.9396
O	−1.7935	6.9800	−3.8994

N	−2.8149	5.1445	−2.9825
C	−3.3136	4.6164	−4.2462
C	−2.8731	3.1662	−4.4712
C	−1.3592	2.9220	−4.3827
C	−1.0594	1.4262	−4.5189
C	−0.5595	3.7485	−5.3952
C	3.6275	1.8571	−1.6994
C	3.1847	3.0070	−2.3650
C	4.0056	3.6439	−3.2928
C	5.2825	3.1437	−3.5595
C	5.7266	1.9997	−2.8971
C	4.9018	1.3541	−1.9724
C	2.7182	1.1315	−0.6978
O	3.3215	0.1963	0.0775
H	2.9407	6.3648	−2.2102
H	2.1760	6.9537	−3.6916
H	−1.5149	5.7481	−0.8713
H	−2.1220	7.3894	−1.1056
H	−3.3799	2.5241	−3.7407
H	−3.2428	2.8505	−5.4573
H	−1.0277	3.2310	−3.3879
H	0.5110	3.5442	−5.2905
H	−0.8526	3.5026	−6.4242
H	−0.7046	4.8213	−5.2437
H	−1.3702	1.0492	−5.5019
H	−1.5868	0.8421	−3.7567
H	0.0116	1.2342	−4.4091
H	−2.9448	5.2808	−5.0291
H	2.2000	3.4191	−2.1738
H	3.6438	4.5289	−3.8078
H	5.9265	3.6426	−4.2781
H	6.7186	1.6094	−3.0972
H	5.2411	0.4678	−1.4513
C	−5.0548	−3.4574	3.2748
C	−3.6244	−2.9736	3.5575
O	−3.4152	−1.8346	3.9676
C	−5.8916	−2.3574	2.5910
C	−5.5574	−2.1751	1.1238
N	−6.4814	−2.5998	0.2351
O	−4.4857	−1.6702	0.7453
N	−2.6223	−3.8851	3.4318
C	−1.2558	−3.5272	3.7915
C	−0.5607	−2.7205	2.6858
O	−1.0194	−2.6798	1.5320
C	−0.5678	−4.9024	3.9028
C	−1.2340	−5.6860	2.7604
C	−2.6950	−5.1974	2.7782
N	0.6164	−2.2073	3.0563
C	1.6006	−1.7314	2.1141
C	2.9940	−2.2256	2.5040
O	3.2147	−3.3829	2.8581
N	3.9103	−1.2372	2.3990
C	5.3389	−1.3998	2.5594
C	5.8785	−0.8465	3.8969
O	7.0885	−0.6452	4.0306
C	6.0756	−0.7637	1.3739
O	5.6745	0.5887	1.1613
N	4.9750	−0.6679	4.8817
C	5.3936	−0.2934	6.2239
H	−6.9551	−2.5883	2.6949
H	−5.6793	−1.3979	3.0716
H	−5.0815	−4.3840	2.6964
H	−0.8012	−5.3506	4.8742
H	0.5164	−4.8235	3.8016
H	−1.1606	−6.7700	2.8787
H	−0.7695	−5.4110	1.8101
H	−3.3401	−5.8798	3.3461
H	−3.0983	−5.1052	1.7642
H	−1.2474	−2.9618	4.7259
H	1.6050	−0.6472	2.0624
H	1.3607	−2.1192	1.1274
H	7.1410	−0.7666	1.5983
H	5.9012	−1.3595	0.4705
H	4.7980	0.5678	0.6738
H	5.5523	−2.4756	2.5732
H	6.1656	0.4773	6.1619
H	2.2151	7.9946	−2.2478
H	−4.4103	4.6659	−4.2609
H	−2.7856	4.5088	−2.1850
H	−0.0387	8.1155	−1.7960
H	5.8149	−1.1501	6.7660

H	4.5202	0.0856	6.7567
H	3.9871	−0.8626	4.7306
H	0.9036	−2.0694	4.0390
H	−7.3580	−3.0423	0.5085
H	−6.3089	−2.4261	−0.7464
H	3.6034	−0.4210	1.8730
H	−5.5025	−3.6777	4.2505
C	−10.7395	−5.3528	1.4564
C	−9.8393	−4.5434	0.5475
O	−8.7375	−4.1481	0.9390
C	−11.2129	−4.4470	2.5980
N	−10.3400	−4.2229	−0.6662
C	−9.6338	−3.3390	−1.5758
C	−8.9818	6.7133	3.3573
C	−8.7204	5.5151	2.4546
C	−8.4653	5.8796	0.9920
N	−8.0489	4.8275	0.2373
O	−8.6454	7.0087	0.5441
H	−10.3508	−4.0300	3.1232
H	−11.8225	−5.0037	3.3151
H	−11.8127	−3.6151	2.2158
H	−11.5906	−5.7748	0.9102
H	−8.6738	−3.7724	−1.8747
H	−7.8710	4.9278	2.8165
H	−9.5789	4.8330	2.4736
H	−7.8354	3.9158	0.6469
H	−7.8466	5.0009	−0.7351
H	−9.8190	7.3059	2.9802
H	−9.2102	6.3859	4.3762
H	−8.1112	7.3744	3.3949
H	−10.1507	−6.1848	1.8536
H	−9.4381	−2.3692	−1.1079
H	−10.2421	−3.1852	−2.4675
H	−11.2550	−4.5602	−0.9185

TS8

C	11.3844	5.2559	−0.5022
C	10.6233	4.1229	−1.2071
C	9.9332	3.1870	−0.2575
C	8.6284	2.7402	−0.2753
N	10.6102	2.6089	0.8010
C	9.7238	1.8322	1.4016
N	8.5081	1.8756	0.7930
H	10.6959	5.8981	0.0568
H	11.3354	3.5529	−1.8206
H	9.8826	4.5377	−1.9017
H	7.7956	2.9597	−0.9261
H	9.9018	1.2231	2.2771
H	7.7018	1.2776	1.0130
C	−9.4925	2.2336	−4.4588
C	−8.5608	2.9333	−3.4544
C	−7.4812	2.0064	−2.9472
C	−6.3482	1.7306	−3.7266
C	−7.6018	1.3471	−1.7187
C	−5.3819	0.8236	−3.3042
C	−6.6462	0.4314	−1.2789
C	−5.5283	0.1625	−2.0772
O	−4.5516	−0.7108	−1.7303
H	−9.9827	1.3725	−3.9929
H	−9.1502	3.3112	−2.6106
H	−8.1064	3.8081	−3.9363
H	−6.2200	2.2353	−4.6815
H	−8.4517	1.5667	−1.0776
H	−4.5058	0.6122	−3.9075
H	−6.7403	−0.0272	−0.3022
H	−4.6598	−1.0938	−0.8310
C	−0.3072	−6.5746	−5.3829
C	−0.9802	−7.3524	−4.2407
C	−1.8976	−6.4765	−3.4199
C	−1.5155	−5.9864	−2.1675
C	−3.1406	−6.0748	−3.9274
C	−2.3255	−5.1124	−1.4405
C	−3.9584	−5.1987	−3.2252
C	−3.5475	−4.7013	−1.9831
O	−4.3802	−3.8221	−1.3671
H	0.2998	−5.7550	−4.9833
H	−1.5453	−8.1929	−4.6632
H	−0.2104	−7.7887	−3.5940
H	−0.5607	−6.2906	−1.7490
H	−3.4663	−6.4446	−4.8969

H	−1.9956	−4.7220	−0.4814
H	−4.9114	−4.8699	−3.6263
H	−3.9716	−3.3489	−0.6154
C	4.9200	−4.1589	−5.7358
C	5.1633	−4.9476	−4.4712
O	6.2588	−5.4325	−4.1924
C	5.2860	−2.6783	−5.5341
H	5.5444	−4.5937	−6.5204
H	6.3337	−2.5829	−5.2379
H	4.6721	−2.2217	−4.7503
H	5.1345	−2.1097	−6.4561
N	4.1003	−5.0225	−3.6313
C	4.2695	−5.5741	−2.3030
C	2.9916	−5.5116	−1.4774
C	2.4035	−4.1054	−1.2635
C	1.2528	−4.1888	−0.2578
C	3.4583	−3.0886	−0.8078
H	3.2495	−4.5283	−3.8514
H	5.0697	−5.0350	−1.7878
H	3.2184	−5.9465	−0.4961
H	2.2243	−6.1544	−1.9316
H	1.9853	−3.7541	−2.2181
H	1.6395	−4.3248	0.7576
H	0.6435	−3.2863	−0.2586
H	0.5866	−5.0264	−0.4850
H	4.2330	−2.9216	−1.5622
H	3.9418	−3.4292	0.1130
H	3.0019	−2.1173	−0.6005
C	9.1997	−5.4333	−1.0448
C	7.8564	−4.6965	−1.0321
C	7.9681	−3.2129	−1.3123
C	7.1751	−2.6226	−2.3025
C	8.8517	−2.3943	−0.5954
C	7.2550	−1.2546	−2.5678
C	8.9460	−1.0303	−0.8641
C	8.1450	−0.4544	−1.8522
H	9.8835	−5.0519	−0.2801
H	7.3714	−4.8460	−0.0569
H	7.2059	−5.1470	−1.7863
H	6.5078	−3.2518	−2.8786
H	9.4702	−2.8265	0.1861
H	6.6212	−0.8204	−3.3351
H	9.6314	−0.4086	−0.2988
H	8.2236	0.6099	−2.0465
C	−2.9444	0.8292	0.5039
C	−1.7051	1.1193	0.0011
C	−1.5526	2.3985	−0.6242
N	−0.3638	2.8499	−1.0090
N	−2.6337	3.2011	−0.8264
C	−3.8143	2.8257	−0.3557
N	−3.9855	1.6865	0.3463
S	1.8972	−0.2358	−2.7929
C	1.3134	0.4846	−1.3419
C	−5.0079	3.6746	−0.6348
N	0.1805	−0.1677	−1.0226
C	−0.2854	−1.1880	−1.8779
C	−1.5889	−1.8610	−1.6276
C	0.5674	−1.3772	−2.9205
C	0.4370	−2.3401	−4.0594
C	−0.5322	0.2116	0.2214
H	2.6060	2.5263	0.1618
H	−0.2586	3.7618	−1.4516
H	−0.8441	−0.7000	0.7314
H	0.2062	0.7214	0.8427
H	−5.6884	3.6574	0.2197
H	−4.6953	4.6904	−0.8769
H	−5.5446	3.2570	−1.4937
H	−3.1590	−0.0827	1.0547
H	−1.6561	−2.2263	−0.6001
H	−1.7022	−2.7085	−2.3015
H	−2.4231	−1.1788	−1.8087
H	−0.1741	−3.2021	−3.7769
H	1.4178	−2.7116	−4.3747
C	1.7971	−3.0753	7.2384
C	0.9829	−1.7806	7.1175
C	1.7655	−0.6949	6.3651
O	1.9504	−0.9182	5.0987
O	2.1842	0.2950	6.9780
C	−8.3520	2.5463	4.0393
C	−7.5284	1.4017	3.4536
C	−6.7271	1.7954	2.2118
O	−6.8570	2.9496	1.7455
O	−5.9491	0.8926	1.7422
H	−8.8777	2.2243	4.9453
H	−7.7131	3.3929	4.2982
H	−8.1670	0.5509	3.1818
H	−6.8131	1.0085	4.1855
C	−0.8567	1.7437	5.4337
C	−0.7570	1.3652	3.9484
C	0.4975	1.8989	3.3401
C	0.7424	2.9292	2.4562
N	1.7475	1.4562	3.7272
C	2.6685	2.2088	3.0724
N	2.1023	3.1182	2.2913
H	0.0082	1.3818	5.9959
H	−0.8076	0.2763	3.8387
H	−1.6131	1.7693	3.3968
H	0.0186	3.5470	1.9422
H	3.7288	2.0406	3.1753
H	0.4604	2.2400	−0.9789
C	5.4150	6.9618	3.0926
C	5.5017	5.5558	3.6950
C	6.1743	4.4931	2.8042
C	6.3368	3.1869	3.5961
C	5.4001	4.2668	1.4971
H	4.7922	6.9762	2.1927
H	4.4908	5.2040	3.9416
H	6.0486	5.6091	4.6460
H	7.1806	4.8514	2.5398
H	5.3834	2.8779	4.0373
H	6.6945	2.3650	2.9710
H	7.0446	3.3144	4.4225
H	4.3510	4.0183	1.6924
H	5.8499	3.4510	0.9227
H	5.4165	5.1584	0.8608
H	−0.0272	−1.8674	−4.9329
H	−9.0931	2.8991	3.3210
H	12.0975	4.8352	0.2111
H	11.9284	5.8769	−1.2222
H	−10.2690	2.9133	−4.8262
H	−8.9253	1.8649	−5.3195
H	−0.8928	2.8319	5.5477
H	−1.7621	1.3201	5.8813
H	6.4082	7.3352	2.8149
H	4.9794	7.6698	3.8058
H	0.0501	−1.9834	6.5751
H	0.7214	−1.3935	8.1059
H	2.0842	−3.4348	6.2455
H	1.2296	−3.8635	7.7454
H	2.7159	−2.8999	7.8077
H	9.0566	−6.5027	−0.8584
H	9.6897	−5.3212	−2.0169
H	4.6121	−6.6108	−2.3957
H	0.3446	−7.2219	−5.9792
H	−1.0555	−6.1348	−6.0498
H	3.8735	−4.2480	−6.0528
H	1.9327	0.6473	4.3571
H	−4.9430	1.4147	0.8759
C	2.1241	6.9351	−2.7354
C	0.8301	6.3154	−2.2726
O	0.6602	5.0919	−2.3117
N	−0.1335	7.1599	−1.8277
C	−1.4949	6.6678	−1.6945
C	−2.0480	6.2791	−3.0771
O	−1.7768	6.9649	−4.0618
N	−2.8332	5.1816	−3.0755
C	−3.3271	4.5883	−4.3108
C	−2.7353	3.1912	−4.5387
C	−1.2007	3.1258	−4.5635
C	−0.7360	1.6684	−4.6470
C	−0.5922	3.9759	−5.6844
C	3.9999	2.2516	−1.5226
C	3.3305	3.1864	−2.3224
C	3.8314	3.5107	−3.5805
C	5.0119	2.9169	−4.0327
C	5.6886	1.9927	−3.2300
C	5.1812	1.6504	−1.9798
C	3.4369	1.9125	−0.2026
O	3.9153	1.0499	0.5323
H	2.9571	6.3153	−2.3990
H	2.1275	6.9414	−3.8303
H	−1.5189	5.8292	−0.9958
H	−2.1091	7.4699	−1.2771
H	−3.1050	2.5269	−3.7482
H	−3.1387	2.7984	−5.4832
H	−0.8323	3.5341	−3.6182
H	0.4993	3.8829	−5.6806
H	−0.9515	3.6487	−6.6689
H	−0.8355	5.0350	−5.5623
H	−1.0847	1.1945	−5.5738
H	−1.1170	1.0781	−3.8065
H	0.3552	1.6026	−4.6254
H	−3.0710	5.2766	−5.1176
H	2.4146	3.6528	−1.9751
H	3.2968	4.2207	−4.2038
H	5.4075	3.1722	−5.0115
H	6.6092	1.5400	−3.5835
H	5.6914	0.9275	−1.3528
C	−5.0485	−3.3743	3.3322
C	−3.6292	−2.8119	3.5322
O	−3.4551	−1.6236	3.7962
C	−5.9344	−2.3794	2.5576
C	−5.5970	−2.3886	1.0864
N	−6.5379	−2.8151	0.2323
O	−4.4674	−2.0254	0.6765
N	−2.5953	−3.6958	3.5042
C	−1.2424	−3.2549	3.8302
C	−0.5587	−2.5581	2.6466
O	−0.9604	−2.7275	1.4859
C	−0.5078	−4.5816	4.1097
C	−1.1567	−5.5334	3.0925
C	−2.6324	−5.0951	3.0576
N	0.5327	−1.8545	2.9885
C	1.5524	−1.4433	2.0490
C	2.8909	−2.0932	2.4084
O	2.9808	−3.2874	2.6925
N	3.9396	−1.2367	2.3510
C	5.3135	−1.6285	2.6071
C	5.8746	−1.0984	3.9439
O	7.0903	−1.1196	4.1407
C	6.2496	−1.2242	1.4630
O	6.3130	0.1903	1.2931
N	4.9798	−0.6509	4.8493
C	5.4349	−0.2171	6.1621
H	−6.9905	−2.6246	2.6989
H	−5.7450	−1.3648	2.9195
H	−5.0478	−4.3541	2.8490
H	−0.7118	−4.9026	5.1364
H	0.5722	−4.4832	3.9797
H	−1.0476	−6.5879	3.3576
H	−0.7040	−5.3783	2.1102
H	−3.2451	−5.7018	3.7371
H	−3.0545	−5.1771	2.0509
H	−1.2661	−2.5831	4.6909
H	1.6502	−0.3571	2.0331
H	1.2677	−1.7808	1.0536
H	7.2549	−1.5557	1.7168
H	5.9363	−1.7107	0.5321
H	5.4778	0.4994	0.8881
H	5.3080	−2.7220	2.6796
H	6.2207	0.5349	6.0511
H	2.2605	7.9584	−2.3778
H	−4.4210	4.5136	−4.2659
H	−2.8006	4.5764	−2.2511
H	−0.0299	8.1336	−2.0724
H	5.8525	−1.0532	6.7365
H	4.5782	0.2041	6.6898
H	3.9771	−0.7771	4.7158
H	0.8236	−1.7205	3.9710
H	−7.3885	−3.2802	0.5485
H	−6.2346	−2.9871	−0.7193
H	3.7591	−0.2782	2.0754
H	−5.4678	−3.5120	4.3356
C	−10.9475	−5.2573	1.5125
C	−9.9716	−4.5506	0.5970
O	−8.8109	−4.3377	0.9581
C	−11.2157	−4.3457	2.7137
N	−10.4633	−4.1031	−0.5797
C	−9.6623	−3.3017	−1.4863
C	−8.9304	6.8139	3.3043
C	−8.8682	5.5698	2.4260
C	−8.8287	5.8655	0.9287
N	−8.4193	4.8009	0.1761

O	−9.1770	6.9377	0.4438
H	−10.2743	−4.0997	3.2096
H	−11.8753	−4.8313	3.4382
H	−11.6883	−3.4103	2.3981
H	−11.8791	−5.5115	0.9948
H	−8.8253	−3.8813	−1.8892
H	−8.0094	4.9423	2.6740
H	−9.7534	4.9443	2.6017
H	−7.9328	4.0224	0.6184
H	−8.2752	4.9700	−0.8082
H	−9.7679	7.4522	3.0115
H	−9.0448	6.5378	4.3570
H	−8.0181	7.4103	3.2057
H	−10.4815	−6.1905	1.8422
H	−9.2530	−2.4258	−0.9750
H	−10.2881	−2.9669	−2.3141
H	−11.4211	−4.3063	−0.8162

EP

C	11.3975	5.2836	−0.3473
C	10.6058	4.1936	−1.0902
C	9.7538	3.3313	−0.2000
C	8.4379	2.9499	−0.3696
N	10.2672	2.7401	0.9404
C	9.2751	2.0205	1.4408
N	8.1465	2.1117	0.6867
H	10.7217	6.0017	0.1287
H	11.3208	3.5533	−1.6266
H	9.9684	4.6479	−1.8587
H	7.6996	3.1964	−1.1177
H	9.3097	1.4203	2.3399
H	7.2860	1.5709	0.8377
C	−9.4586	2.2672	−4.4164
C	−8.5475	2.9902	−3.4078
C	−7.4398	2.0973	−2.8970
C	−6.3018	1.8511	−3.6802
C	−7.5342	1.4453	−1.6625
C	−5.3066	0.9760	−3.2577
C	−6.5477	0.5630	−1.2221
C	−5.4288	0.3208	−2.0252
O	−4.4174	−0.5080	−1.6646
H	−9.9268	1.3922	−3.9534
H	−9.1469	3.3523	−2.5643
H	−8.1176	3.8778	−3.8891
H	−6.1958	2.3501	−4.6409
H	−8.3762	1.6503	−1.0079
H	−4.4295	0.7839	−3.8655
H	−6.6166	0.1133	−0.2387
H	−4.5840	−0.9797	−0.8208
C	−0.2437	−6.4960	−5.4655
C	−1.0092	−7.2104	−4.3386
C	−1.9212	−6.2808	−3.5687
C	−1.6730	−5.9312	−2.2371
C	−3.0363	−5.7053	−4.1951
C	−2.4825	−5.0230	−1.5507
C	−3.8550	−4.8013	−3.5308
C	−3.5746	−4.4453	−2.2060
O	−4.3908	−3.5267	−1.6277
H	0.3972	−5.7085	−5.0547
H	−1.5944	−8.0330	−4.7701
H	−0.2919	−7.6705	−3.6496
H	−0.8287	−6.3724	−1.7148
H	−3.2613	−5.9640	−5.2270
H	−2.2423	−4.7300	−0.5317
H	−4.7059	−4.3414	−4.0222
H	−4.0412	−3.1650	−0.7847
C	4.9771	−4.0588	−5.7614
C	5.2308	−4.8763	−4.5158
O	6.3290	−5.3580	−4.2446
C	5.2884	−2.5738	−5.5042
H	5.6282	−4.4454	−6.5495
H	6.3323	−2.4524	−5.2040
H	4.6612	−2.1697	−4.7022
H	5.1167	−1.9751	−6.4033
N	4.1652	−4.9760	−3.6825
C	4.3211	−5.5350	−2.3556
C	3.0291	−5.4523	−1.5583
C	2.4722	−4.0325	−1.3497
C	1.2210	−4.1293	−0.4766
C	3.5169	−3.0767	−0.7623
H	3.3179	−4.4739	−3.8979
H	5.1228	−5.0065	−1.8324
H	3.2155	−5.8985	−0.5739
H	2.2610	−6.0729	−2.0413
H	2.1571	−3.6307	−2.3255
H	1.4837	−4.4109	0.5487
H	0.6737	−3.1906	−0.4270
H	0.5383	−4.8764	−0.8890
H	4.3748	−2.9443	−1.4285
H	3.8838	−3.4580	0.1956
H	3.1026	−2.0815	−0.5824
C	9.2472	−5.4011	−1.0807
C	7.9088	−4.6715	−0.9182
C	8.0001	−3.1863	−1.1922
C	7.2672	−2.6091	−2.2350
C	8.8229	−2.3560	−0.4185
C	7.3417	−1.2385	−2.4904
C	8.9112	−0.9894	−0.6773
C	8.1648	−0.4239	−1.7134
H	10.0093	−4.9991	−0.4057
H	7.5291	−4.8324	0.0998
H	7.1870	−5.1178	−1.6065
H	6.6541	−3.2493	−2.8588
H	9.3921	−2.7820	0.4037
H	6.7534	−0.8115	−3.2974
H	9.5437	−0.3557	−0.0644
H	8.2299	0.6433	−1.8948
C	−2.7429	0.7728	0.5968
C	−1.5418	1.1064	0.0253
C	−1.4776	2.3866	−0.6104
N	−0.3403	2.8708	−1.0966
N	−2.5852	3.1622	−0.7341
C	−3.7210	2.7521	−0.1893
N	−3.8170	1.6020	0.5084
S	1.9758	−0.2874	−2.9700
C	1.4802	0.4445	−1.5036
C	−4.9437	3.5845	−0.3831
N	0.3362	−0.1301	−1.1024
C	−0.2038	−1.1324	−1.9343
C	−1.5091	−1.7735	−1.6229
C	0.5923	−1.3593	−3.0148
C	0.3682	−2.3271	−4.1359
C	−0.3151	0.2499	0.1803
H	2.2910	2.1382	0.0316
H	−0.2719	3.8063	−1.4976
H	−0.5673	−0.6705	0.7084
H	0.4338	0.7865	0.7644
H	−5.6987	3.3716	0.3752
H	−4.6739	4.6419	−0.3861
H	−5.3792	3.3395	−1.3586
H	−2.9005	−0.1511	1.1472
H	−1.5432	−2.1380	−0.5930
H	−1.6586	−2.6223	−2.2873
H	−2.3374	−1.0788	−1.7805
H	−0.1979	−3.1972	−3.7924
H	1.3191	−2.6834	−4.5440
C	1.8137	−3.2088	7.2203
C	0.9893	−1.9190	7.1117
C	1.7557	−0.8185	6.3610
O	1.9359	−1.0334	5.0937
O	2.1646	0.1723	6.9794
C	−8.3433	2.4365	4.0891
C	−7.4553	1.3073	3.5677
C	−6.7744	1.6322	2.2341
O	−7.2122	2.5863	1.5558
O	−5.7973	0.8651	1.9140
H	−8.8568	2.1390	5.0102
H	−7.7520	3.3288	4.3082
H	−8.0409	0.3893	3.4192
H	−6.6714	1.0444	4.2856
C	−0.8497	1.6325	5.4913
C	−0.7933	1.1980	4.0209
C	0.4209	1.7347	3.3388
C	0.5989	2.7447	2.4161
N	1.6952	1.2955	3.6382
C	2.5657	2.0256	2.8910
N	1.9416	2.9195	2.1358
H	0.0393	1.3047	6.0370
H	−0.8169	0.1048	3.9553
H	−1.6809	1.5563	3.4874
H	−0.1614	3.3597	1.9531
H	3.6314	1.8598	2.9153
H	0.5128	2.3317	−1.0516
C	5.4127	6.9092	3.2590
C	5.4262	5.4917	3.8398
C	6.0008	4.3990	2.9174
C	6.0862	3.0725	3.6874
C	5.1877	4.2506	1.6221
H	4.7670	6.9768	2.3777
H	4.4028	5.2030	4.1160
H	6.0037	5.4965	4.7742
H	7.0246	4.6898	2.6376
H	5.1168	2.8157	4.1285
H	6.3874	2.2411	3.0459
H	6.8055	3.1417	4.5110
H	4.1281	4.0619	1.8253
H	5.5672	3.4194	1.0199
H	5.2521	5.1525	1.0033
H	−0.1889	−1.8636	−4.9579
H	−9.0910	2.7116	3.3437
H	12.0047	4.8292	0.4395
H	12.0553	5.8291	−1.0325
H	−10.2516	2.9272	−4.7837
H	−8.8798	1.9155	−5.2766
H	−0.9013	2.7237	5.5648
H	−1.7322	1.2125	5.9857
H	6.4195	7.2212	2.9560
H	5.0455	7.6338	3.9939
H	0.0547	−2.1259	6.5733
H	0.7296	−1.5408	8.1041
H	2.1002	−3.5591	6.2238
H	1.2542	−4.0047	7.7242
H	2.7335	−3.0304	7.7870
H	9.1382	−6.4696	−0.8669
H	9.6178	−5.2955	−2.1049
H	4.6497	−6.5763	−2.4474
H	0.3897	−7.1950	−6.0212
H	−0.9323	−6.0243	−6.1734
H	3.9390	−4.1756	−6.0967
H	1.9199	0.5047	4.2802
H	−4.7668	1.3192	1.0533
C	2.1386	6.9732	−2.5782
C	0.8261	6.3624	−2.1631
O	0.6355	5.1453	−2.2628
N	−0.1310	7.1959	−1.6885
C	−1.4941	6.7060	−1.5655
C	−2.0432	6.3244	−2.9522
O	−1.7792	7.0218	−3.9298
N	−2.8181	5.2185	−2.9642
C	−3.3009	4.6376	−4.2098
C	−2.6475	3.2779	−4.4897
C	−1.1146	3.2955	−4.5908
C	−0.5707	1.8652	−4.6677
C	−0.6117	4.1527	−5.7576
C	3.7048	1.9731	−1.6271
C	3.2151	3.0900	−2.3147
C	3.9311	3.6293	−3.3802
C	5.1541	3.0683	−3.7569
C	5.6511	1.9629	−3.0652
C	4.9272	1.4105	−2.0070
C	2.9316	1.3960	−0.4667
O	3.4542	0.4802	0.2440
H	2.9524	6.3479	−2.2062
H	2.1849	6.9727	−3.6719
H	−1.5234	5.8634	−0.8708
H	−2.1083	7.5074	−1.1474
H	−2.9435	2.5863	−3.6914
H	−3.0762	2.8750	−5.4187
H	−0.7228	3.7452	−3.6748
H	0.4814	4.1137	−5.8149
H	−1.0092	3.7920	−6.7153
H	−0.8989	5.2006	−5.6347
H	−0.9179	1.3558	−5.5760
H	−0.8953	1.2700	−3.8071
H	0.5232	1.8625	−4.6780
H	−3.0913	5.3610	−4.9988
H	2.2763	3.5504	−2.0258
H	3.5319	4.4863	−3.9143
H	5.7179	3.4919	−4.5830
H	6.6049	1.5306	−3.3498
H	5.3100	0.5547	−1.4640
C	−5.0197	−3.4611	3.2896

C	−3.5920	−2.9350	3.5020
O	−3.3916	−1.7564	3.7904
C	−5.8806	−2.4308	2.5310
C	−5.5552	−2.4182	1.0560
N	−6.5118	−2.8197	0.2070
O	−4.4273	−2.0549	0.6390
N	−2.5802	−3.8415	3.4582
C	−1.2199	−3.4278	3.7817
C	−0.5520	−2.6723	2.6267
O	−0.9750	−2.7606	1.4628
C	−0.4897	−4.7714	3.9808
C	−1.1656	−5.6658	2.9302
C	−2.6394	−5.2218	2.9579
N	0.5627	−2.0244	2.9893
C	1.5752	−1.5978	2.0521
C	2.9339	−2.1858	2.4407
O	3.0801	−3.3728	2.7321
N	3.9168	−1.2587	2.4029
C	5.3278	−1.5296	2.5793
C	5.8940	−0.9969	3.9123
O	7.1133	−0.8825	4.0565
C	6.1379	−0.9674	1.4059
O	5.9183	0.4329	1.2339
N	4.9973	−0.7198	4.8796
C	5.4458	−0.3216	6.2040
H	−6.9423	−2.6504	2.6728
H	−5.6638	−1.4276	2.9106
H	−5.0414	−4.4322	2.7898
H	−0.6776	−5.1431	4.9935
H	0.5884	−4.6714	3.8373
H	−1.0538	−6.7337	3.1335
H	−0.7382	−5.4561	1.9465
H	−3.2337	−5.8532	3.6315
H	−3.0932	−5.2623	1.9626
H	−1.2209	−2.8016	4.6767
H	1.6386	−0.5131	2.0063
H	1.3169	−1.9684	1.0631
H	7.1944	−1.1058	1.6271
H	5.8910	−1.5099	0.4865
H	5.0534	0.5524	0.7742
H	5.4473	−2.6191	2.6042
H	6.2029	0.4619	6.1157
H	2.2700	7.9967	−2.2195
H	−4.3887	4.5084	−4.1492
H	−2.7948	4.6068	−2.1461
H	−0.0121	8.1817	−1.8670
H	5.8970	−1.1638	6.7438
H	4.5798	0.0495	6.7540
H	4.0012	−0.8808	4.7404
H	0.8516	−1.9010	3.9739
H	−7.3597	−3.2887	0.5244
H	−6.2350	−2.9551	−0.7589
H	3.6671	−0.3645	1.9925
H	−5.4461	−3.6055	4.2889
C	−10.9034	−5.3300	1.4212
C	−9.9281	−4.6051	0.5191
O	−8.7669	−4.3967	0.8814
C	−11.1820	−4.4402	2.6364
N	−10.4192	−4.1422	−0.6520
C	−9.6198	−3.3192	−1.5404
C	−8.9328	6.7144	3.4262
C	−8.8040	5.5402	2.4592
C	−8.6113	5.9567	1.0019
N	−8.1876	4.9432	0.1943
O	−8.8511	7.0883	0.5909
H	−10.2440	−4.1953	3.1396
H	−11.8400	−4.9428	3.3507
H	−11.6608	−3.5031	2.3352
H	−11.8316	−5.5812	0.8959
H	−8.7561	−3.8764	−1.9163
H	−7.9850	4.8727	2.7376
H	−9.7094	4.9201	2.4979
H	−7.8754	4.0529	0.5751
H	−8.0001	5.1665	−0.7708
H	−9.7313	7.3885	3.1079
H	−9.1486	6.3584	4.4382
H	−8.0085	7.2994	3.4590
H	−10.4335	−6.2659	1.7374
H	−9.2507	−2.4276	−1.0248
H	−10.2328	−3.0094	−2.3874
H	−11.3822	−4.3262	−0.8833

OC1

C	11.5049	5.3282	−0.3546
C	11.0289	4.1798	−1.2562
C	9.8532	3.4389	−0.6920
C	8.6168	3.2180	−1.2598
N	9.9082	2.8469	0.5566
C	8.7265	2.2796	0.7310
N	7.9070	2.4769	−0.3401
H	10.7248	6.0894	−0.2488
H	11.8611	3.4774	−1.4009
H	10.7689	4.5619	−2.2509
H	8.1899	3.5156	−2.2055
H	8.4059	1.7028	1.5889
H	6.9572	2.1070	−0.4212
C	−9.3509	2.3864	−4.4786
C	−7.9584	2.7553	−3.9416
C	−7.1964	1.5628	−3.4067
C	−6.8220	0.5173	−4.2632
C	−6.8646	1.4490	−2.0517
C	−6.1386	−0.5968	−3.7925
C	−6.1719	0.3390	−1.5624
C	−5.7920	−0.6862	−2.4378
O	−5.0877	−1.7791	−2.0409
H	−9.9628	1.9401	−3.6879
H	−8.0584	3.5041	−3.1477
H	−7.3824	3.2345	−4.7446
H	−7.0593	0.5825	−5.3226
H	−7.1517	2.2249	−1.3468
H	−5.8260	−1.3915	−4.4622
H	−5.9304	0.2743	−0.5074
H	−4.8053	−1.6829	−1.0931
C	−0.1922	−6.4571	−5.3295
C	−0.2062	−5.3536	−4.2620
C	−1.5409	−5.2332	−3.5631
C	−1.7445	−5.7687	−2.2884
C	−2.6280	−4.6007	−4.1859
C	−2.9835	−5.6828	−1.6535
C	−3.8701	−4.5029	−3.5672
C	−4.0511	−5.0543	−2.2961
O	−5.3003	−4.9722	−1.7362
H	−0.9472	−6.2655	−6.0991
H	0.5772	−5.5496	−3.5217
H	0.0500	−4.3986	−4.7334
H	−0.9216	−6.2587	−1.7765
H	−2.4941	−4.1623	−5.1718
H	−3.1157	−6.0980	−0.6571
H	−4.6980	−3.9826	−4.0334
H	−5.2475	−5.2223	−0.8037
C	5.0469	−4.0615	−5.6401
C	5.2456	−4.9497	−4.4308
O	6.2340	−5.6679	−4.2842
C	4.7353	−2.5957	−5.2949
H	5.9677	−4.1274	−6.2244
H	5.4701	−2.1964	−4.5917
H	3.7452	−2.4692	−4.8428
H	4.7509	−1.9758	−6.1961
N	4.2344	−4.8906	−3.5316
C	4.3614	−5.4739	−2.2133
C	3.0527	−5.3534	−1.4394
C	2.4902	−3.9248	−1.3194
C	1.2191	−3.9506	−0.4738
C	3.5120	−2.9282	−0.7574
H	3.5387	−4.1705	−3.6465
H	5.1694	−4.9790	−1.6647
H	3.2216	−5.7459	−0.4289
H	2.2939	−5.9988	−1.9025
H	2.1919	−3.5719	−2.3205
H	1.4267	−4.3043	0.5412
H	0.7832	−2.9563	−0.3975
H	0.4587	−4.6084	−0.9053
H	4.4059	−2.8500	−1.3851
H	3.8367	−3.2330	0.2442
H	3.0812	−1.9269	−0.6763
C	9.2855	−5.3524	−0.9150
C	7.9101	−4.8441	−1.3730
C	7.9353	−3.3823	−1.7628
C	7.9004	−2.9815	−3.1040
C	8.0394	−2.3942	−0.7755
C	7.9722	−1.6263	−3.4410
C	8.0972	−1.0414	−1.1045
C	8.0689	−0.6520	−2.4456
H	9.6522	−4.7797	−0.0571
H	7.1953	−4.9848	−0.5516
H	7.5489	−5.4417	−2.2145
H	7.8123	−3.7387	−3.8769
H	8.0772	−2.6937	0.2671
H	7.9629	−1.3336	−4.4882
H	8.1616	−0.2933	−0.3230
H	8.1293	0.4009	−2.7022
C	−3.2228	0.8688	1.0273
C	−1.9859	0.8097	0.4582
C	−1.6148	1.8807	−0.4086
N	−0.4024	1.8828	−0.9853
N	−2.4642	2.9115	−0.6513
C	−3.6621	2.8969	−0.0698
N	−4.0551	1.9117	0.7581
S	1.2386	0.3199	−2.5405
C	0.5346	0.7183	−0.8978
C	−4.6132	4.0188	−0.3471
N	−0.1686	−0.4756	−0.4995
C	−0.6645	−1.2024	−1.6149
C	−1.7641	−2.1836	−1.3840
C	−0.0126	−0.9452	−2.7643
C	−0.1223	−1.6027	−4.0986
C	−0.9937	−0.2890	0.6905
H	1.3480	0.9499	−0.2112
H	−0.1207	2.6834	−1.5450
H	−1.4807	−1.2229	0.9616
H	−0.3328	−0.0182	1.5158
H	−4.8038	4.0755	−1.4221
H	−5.5617	3.8703	0.1721
H	−4.1557	4.9680	−0.0522
H	−3.5978	0.1005	1.6946
H	−1.5153	−2.8670	−0.5675
H	−1.9609	−2.7685	−2.2791
H	−2.6888	−1.6721	−1.1105
H	−0.9375	−2.3284	−4.1026
H	0.8052	−2.1317	−4.3588
C	1.8242	−2.9661	7.3085
C	1.0682	−1.6562	7.5745
C	1.6714	−0.4747	6.8377
O	1.5235	−0.5904	5.5124
O	2.2102	0.4734	7.3766
C	−8.2771	2.6950	4.0281
C	−8.4352	1.8873	2.7283
C	−7.2485	2.1190	1.7932
O	−7.3290	3.0196	0.9205
O	−6.2151	1.3999	1.9979
H	−9.0899	2.4732	4.7274
H	−7.3298	2.4447	4.5150
H	−9.3547	2.1793	2.2121
H	−8.4879	0.8201	2.9663
C	−0.7966	1.8630	5.4827
C	−0.7500	1.4096	4.0221
C	0.5257	1.7874	3.3391
C	0.7171	2.5411	2.2074
N	1.7660	1.3531	3.7943
C	2.6676	1.8227	2.9419
N	2.0766	2.5599	1.9745
H	0.0685	1.5088	6.0442
H	−0.8881	0.3239	3.9640
H	−1.5872	1.8452	3.4664
H	0.0229	3.0572	1.5653
H	3.7282	1.6280	2.9700
H	2.5144	2.8661	1.0975
C	5.5138	7.0572	3.1923
C	5.5135	5.7114	3.9255
C	6.0191	4.5068	3.1082
C	5.9960	3.2412	3.9779
C	5.2067	4.3030	1.8230
H	4.8262	7.0540	2.3409
H	4.4932	5.4891	4.2698
H	6.1259	5.7970	4.8328
H	7.0626	4.6998	2.8190
H	4.9806	3.0443	4.3436
H	6.3321	2.3552	3.4301
H	6.6386	3.3555	4.8580
H	4.1372	4.2451	2.0525
H	5.4981	3.3789	1.3168
H	5.3463	5.1232	1.1125

H	−0.3138	−0.8750	−4.8955
H	−8.2880	3.7702	3.8364
H	11.7319	4.9449	0.6436
H	12.4024	5.8086	−0.7599
H	−9.8763	3.2676	−4.8622
H	−9.2786	1.6572	−5.2919
H	−0.7993	2.9560	5.5483
H	−1.7019	1.4922	5.9741
H	6.5125	7.2979	2.8094
H	5.2081	7.8689	3.8608
H	0.0277	−1.7553	7.2411
H	1.0625	−1.4124	8.6392
H	1.9420	−3.1429	6.2361
H	1.2962	−3.8148	7.7534
H	2.8281	−2.9252	7.7415
H	9.2379	−6.4072	−0.6245
H	10.0220	−5.2529	−1.7191
H	4.6555	−6.5217	−2.3236
H	0.7854	−6.5207	−5.8189
H	−0.4147	−7.4320	−4.8841
H	4.2427	−4.4791	−6.2603
H	1.7863	0.2510	4.9772
H	−5.0629	1.8319	1.2371
C	2.2695	7.0431	−2.6620
C	1.1027	6.1838	−2.2030
O	1.0270	4.9784	−2.4367
N	0.1238	6.8489	−1.5135
C	−1.1767	6.2135	−1.3986
C	−1.8358	6.1107	−2.7849
O	−1.6714	6.9949	−3.6228
N	−2.5884	5.0077	−2.9780
C	−3.1777	4.7174	−4.2810
C	−3.5502	3.2393	−4.3953
C	−2.4052	2.2509	−4.1092
C	−2.9092	0.8144	−4.2554
C	−1.1606	2.5151	−4.9627
C	4.1167	2.0857	−2.5358
C	3.4964	2.9775	−3.4273
C	3.7921	2.9068	−4.7844
C	4.7014	1.9556	−5.2574
C	5.3189	1.0695	−4.3716
C	5.0266	1.1279	−3.0123
C	3.7328	2.1318	−1.1103
O	2.9778	3.0026	−0.6655
C	4.2800	1.0737	−0.1454
O	5.5372	1.0984	0.0003
O	3.4540	0.3475	0.4430
H	3.1909	6.4631	−2.5873
H	2.1115	7.3050	−3.7132
H	−1.0648	5.2315	−0.9375
H	−1.8063	6.8262	−0.7465
H	−4.3854	3.0133	−3.7187
H	−3.9317	3.0665	−5.4107
H	−2.1096	2.3737	−3.0627
H	−0.3869	1.7725	−4.7466
H	−1.3977	2.4639	−6.0332
H	−0.7271	3.4980	−4.7531
H	−3.1578	0.5859	−5.2996
H	−3.8115	0.6549	−3.6619
H	−2.1564	0.1020	−3.9138
H	−2.4562	5.0067	−5.0514
H	2.7697	3.6901	−3.0477
H	3.3045	3.5858	−5.4773
H	4.9263	1.9029	−6.3190
H	6.0264	0.3312	−4.7337
H	5.5089	0.4434	−2.3249
C	−4.9909	−3.2389	3.3477
C	−3.5792	−2.6856	3.5560
O	−3.3189	−1.4851	3.4576
C	−5.9024	−2.2964	2.5392
C	−5.5938	−2.3523	1.0547
N	−6.3849	−3.1301	0.2919
O	−4.6249	−1.7361	0.5729
N	−2.6444	−3.5852	3.9606
C	−1.2901	−3.1360	4.2628
C	−0.5296	−2.7524	2.9879
O	−0.7959	−3.2685	1.9015
C	−0.6290	−4.3837	4.8821
C	−1.3119	−5.5306	4.1238
C	−2.7614	−5.0496	3.9564
N	0.4612	−1.8671	3.2137
C	1.5722	−1.6663	2.3114
C	2.8485	−2.2491	2.9249
O	2.8281	−3.2412	3.6530
N	3.9726	−1.5669	2.5976
C	5.2905	−2.0383	2.9870
C	5.6883	−1.7299	4.4365
O	6.6466	−2.3107	4.9404
C	6.3530	−1.3910	2.0867
O	6.3883	0.0140	2.2627
N	4.9900	−0.7463	5.0492
C	5.4815	−0.1219	6.2612
H	−6.9460	−2.5818	2.7035
H	−5.7621	−1.2627	2.8646
H	−4.9597	−4.2256	2.8768
H	−0.8547	−4.4287	5.9528
H	0.4552	−4.3676	4.7532
H	−1.2509	−6.4903	4.6430
H	−0.8524	−5.6336	3.1383
H	−3.4006	−5.3903	4.7826
H	−3.1977	−5.4070	3.0193
H	−1.3220	−2.2842	4.9474
H	1.7099	−0.6084	2.0887
H	1.3512	−2.1752	1.3755
H	7.3208	−1.8226	2.3659
H	6.1398	−1.6466	1.0427
H	6.0625	0.4219	1.4269
H	5.3387	−3.1268	2.8833
H	6.2142	0.6643	6.0371
H	2.3667	7.9629	−2.0803
H	−4.0657	5.3431	−4.4352
H	−2.5384	4.2698	−2.2795
H	0.0970	7.8489	−1.6568
H	5.9736	−0.8860	6.8645
H	4.6452	0.3091	6.8150
H	4.2768	−0.2845	4.5068
H	0.6385	−1.5845	4.1739
H	−7.2446	−3.5648	0.6212
H	−6.1898	−3.1615	−0.7046
H	3.9056	−0.8325	1.8869
H	−5.4223	−3.3920	4.3450
C	−11.0678	−5.0585	1.4227
C	−10.0077	−4.4764	0.5099
O	−8.8145	−4.5153	0.8249
C	−11.1566	−4.1864	2.6796
N	−10.4539	−3.8657	−0.6095
C	−9.5656	−3.1483	−1.5077
C	−8.8334	6.9642	3.2890
H	−10.1773	−4.1269	3.1599
H	−11.8717	−4.5990	3.3968
H	−11.4748	−3.1693	2.4298
H	−12.0405	−5.1258	0.9226
H	−8.7555	−3.8016	−1.8436
H	−8.4090	6.8968	4.2958
H	−8.9286	8.0159	3.0111
H	−9.8406	6.5345	3.3176
H	−10.7571	−6.0731	1.6882
H	−9.1157	−2.2775	−1.0201
H	−10.1321	−2.8132	−2.3768
H	−11.4466	−3.8372	−0.7791
C	−7.9607	6.2446	2.2669
C	−8.4789	6.4980	0.8483
N	−8.5553	5.3965	0.0619
O	−8.8076	7.6222	0.4739
H	−6.9393	6.6444	2.3065
H	−7.8862	5.1724	2.4565
H	−8.2334	4.4782	0.3752
H	−8.8439	5.5361	−0.8945

OC2

C	11.2468	5.3073	0.4898
C	10.6435	4.2025	−0.3988
C	9.5953	3.3638	0.2802
C	8.3210	3.0512	−0.1485
N	9.8461	2.7403	1.4898
C	8.7433	2.0724	1.7825
N	7.7885	2.2239	0.8198
H	10.4824	6.0354	0.7806
H	11.4589	3.5472	−0.7358
H	10.2116	4.6442	−1.3053
H	7.7575	3.3273	−1.0268
H	8.5739	1.4639	2.6607
H	6.8502	1.8180	0.8441
C	−9.4742	2.4016	−4.2878
C	−8.4028	3.0561	−3.3870
C	−7.2995	2.1243	−2.9191
C	−6.2063	1.8155	−3.7455
C	−7.3151	1.5554	−1.6397
C	−5.1907	0.9586	−3.3264
C	−6.3095	0.6908	−1.2018
C	−5.2445	0.3733	−2.0523
O	−4.2325	−0.4678	−1.7054
H	−9.9788	1.5859	−3.7596
H	−8.8966	3.4893	−2.5091
H	−7.9565	3.8967	−3.9338
H	−6.1470	2.2568	−4.7384
H	−8.1062	1.8055	−0.9389
H	−4.3469	0.7270	−3.9689
H	−6.3406	0.3160	−0.1847
H	−4.3894	−0.9148	−0.8465
C	−0.1081	−6.1052	−5.9356
C	−0.0041	−4.7734	−5.1830
C	−1.1768	−4.5176	−4.2650
C	−1.1049	−4.8351	−2.9052
C	−2.3645	−3.9473	−4.7430
C	−2.1529	−4.5493	−2.0338
C	−3.4270	−3.6647	−3.8870
C	−3.3129	−3.9423	−2.5217
O	−4.3535	−3.6155	−1.6976
H	−1.0125	−6.1348	−6.5516
H	0.9228	−4.7663	−4.5966
H	0.0795	−3.9525	−5.9058
H	−0.1981	−5.2839	−2.5155
H	−2.4462	−3.6883	−5.7961
H	−2.0545	−4.7319	−0.9689
H	−4.3281	−3.1779	−4.2441
H	−4.0257	−3.2121	−0.8647
C	5.0800	−3.5823	−5.8945
C	5.2876	−4.5251	−4.7323
O	6.3714	−5.0616	−4.5029
C	5.1824	−2.1137	−5.4436
H	5.8531	−3.8076	−6.6327
H	6.1401	−1.9265	−4.9517
H	4.3934	−1.8508	−4.7326
H	5.0965	−1.4368	−6.2982
N	4.2021	−4.7070	−3.9313
C	4.3778	−5.3734	−2.6531
C	3.0899	−5.5361	−1.8596
C	2.3307	−4.2435	−1.5003
C	1.3274	−4.5575	−0.3837
C	3.2538	−3.0924	−1.0818
H	3.3983	−4.1093	−4.0582
H	5.1098	−4.8198	−2.0539
H	3.3656	−6.0418	−0.9256
H	2.4061	−6.2147	−2.3877
H	1.7652	−3.9138	−2.3862
H	1.8527	−4.6602	0.5716
H	0.5799	−3.7713	−0.2676
H	0.7936	−5.4939	−0.5827
H	3.9308	−2.7947	−1.8890
H	3.8514	−3.3697	−0.2089
H	2.6589	−2.2146	−0.8126
C	9.2740	−5.2924	−1.2653
C	7.9394	−4.5504	−1.4610
C	8.1515	−3.0723	−1.6960
C	8.2465	−2.5644	−2.9992
C	8.3380	−2.1924	−0.6215
C	8.5420	−1.2164	−3.2162
C	8.6286	−0.8449	−0.8348
C	8.7369	−0.3534	−2.1360
H	9.8264	−4.8885	−0.4106
H	7.3124	−4.6972	−0.5724
H	7.4095	−4.9841	−2.3136
H	8.0844	−3.2373	−3.8369
H	8.2610	−2.5708	0.3952
H	8.6191	−0.8411	−4.2337
H	8.7739	−0.1765	0.0069
H	8.9598	0.6968	−2.2943
C	−2.4825	0.3608	0.7836
C	−1.3269	0.7263	0.1561
C	−1.2714	2.0321	−0.4040
N	−0.1204	2.4696	−0.9304

N	−2.3408	2.8660	−0.3644
C	−3.4349	2.4550	0.2678
N	−3.5259	1.2434	0.8514
S	0.9345	1.8437	−3.3350
C	0.9329	1.5775	−1.4908
C	−4.6087	3.3769	0.3600
N	0.5485	0.1815	−1.3068
C	−0.2112	−0.2995	−2.4172
C	−0.9989	−1.5472	−2.2285
C	−0.1274	0.4292	−3.5413
C	−0.7452	0.1656	−4.8769
C	−0.0695	−0.0718	0.0129
H	2.2590	3.0649	−0.7238
H	−0.0564	3.4459	−1.2328
H	−0.2756	−1.1318	0.1382
H	0.6537	0.2407	0.7622
H	−5.5369	2.8318	0.5371
H	−4.4557	4.0797	1.1871
H	−4.6844	3.9610	−0.5588
H	−2.6392	−0.6090	1.2448
H	−0.4292	−2.3151	−1.7025
H	−1.3057	−1.9583	−3.1863
H	−1.9080	−1.3616	−1.6493
H	−1.2575	−0.7992	−4.8833
H	0.0080	0.1548	−5.6748
C	1.6416	−3.9706	7.0392
C	1.1221	−2.5413	7.1718
C	1.7431	−1.6026	6.1371
O	2.0514	−2.0972	5.0132
O	1.9082	−0.3914	6.4636
C	−8.5343	1.8035	4.2196
C	−7.0242	1.6418	3.9931
C	−6.5981	1.3935	2.5334
O	−7.2504	1.9519	1.6169
O	−5.5563	0.6735	2.3684
H	−8.7503	2.3239	5.1578
H	−8.9776	2.3574	3.3940
H	−6.5997	0.8538	4.6210
H	−6.5076	2.5686	4.2824
C	−1.0594	0.9734	5.7037
C	−0.8046	0.5623	4.2528
C	0.4029	1.2477	3.7125
C	0.5672	2.1635	2.7001
N	1.6648	1.0575	4.2437
C	2.5256	1.8249	3.5377
N	1.8996	2.5151	2.5850
H	−0.2192	0.6837	6.3397
H	−0.6887	−0.5267	4.1885
H	−1.6638	0.8183	3.6244
H	−0.1830	2.5735	2.0414
H	3.5888	1.8380	3.7195
H	2.3738	1.9411	1.1014
C	5.1663	6.5155	4.0997
C	5.4241	5.1022	4.6165
C	5.9333	4.1150	3.5534
C	6.1323	2.7350	4.1919
C	5.0009	4.0721	2.3333
H	4.3541	6.5337	3.3672
H	4.4940	4.7002	5.0428
H	6.1472	5.1393	5.4423
H	6.9169	4.4630	3.2048
H	5.2282	2.4121	4.7212
H	6.3703	1.9669	3.4578
H	6.9395	2.7551	4.9317
H	3.9555	3.9279	2.6227
H	5.2722	3.2558	1.6630
H	5.0567	5.0039	1.7609
H	−1.4795	0.9370	−5.1378
H	−9.0265	0.8251	4.2585
H	11.6517	4.8675	1.4047
H	12.0493	5.8379	−0.0335
H	−10.2293	3.1341	−4.5912
H	−9.0208	1.9815	−5.1919
H	−1.1913	2.0578	5.7779
H	−1.9647	0.4922	6.0881
H	6.0591	6.9281	3.6143
H	4.8891	7.1930	4.9149
H	0.0332	−2.5147	7.0229
H	1.3074	−2.1292	8.1679
H	1.4559	−4.3569	6.0344
H	1.1671	−4.6401	7.7655

H	2.7237	−4.0089	7.2002
H	9.1090	−6.3615	−1.0938
H	9.9059	−5.1824	−2.1523
H	4.8262	−6.3524	−2.8461
H	0.7568	−6.2635	−6.5882
H	−0.1613	−6.9421	−5.2314
H	4.1033	−3.7626	−6.3605
H	1.8849	0.4664	5.0971
H	−4.4348	0.9809	1.4000
C	2.0106	7.0719	−1.7764
C	0.8156	6.2204	−1.4144
O	0.8229	5.0034	−1.6277
N	−0.2287	6.8661	−0.8366
C	−1.5222	6.2150	−0.6812
C	−2.2535	6.2224	−2.0316
O	−2.3350	7.2772	−2.6637
N	−2.7337	5.0281	−2.4314
C	−3.3585	4.8243	−3.7320
C	−2.4521	4.0800	−4.7220
C	−1.1266	4.7746	−5.0814
C	−0.3595	3.9292	−6.1049
C	−1.3303	6.1980	−5.6165
C	3.5210	1.7218	−1.7788
C	4.1440	2.7792	−2.4432
C	5.3074	2.5585	−3.1830
C	5.8652	1.2817	−3.2422
C	5.2499	0.2210	−2.5714
C	4.0772	0.4404	−1.8542
C	2.3270	1.9832	−0.8871
O	2.5610	1.3166	0.3436
H	2.9035	6.6320	−1.3253
H	2.1384	7.0430	−2.8621
H	−1.3796	5.2108	−0.2843
H	−2.1099	6.7908	0.0385
H	−2.2299	3.0856	−4.3128
H	−3.0343	3.9157	−5.6407
H	−0.5203	4.8279	−4.1677
H	−0.3709	6.6283	−5.9260
H	−1.9839	6.1906	−6.4990
H	−1.7674	6.8581	−4.8644
H	−0.9123	3.8691	−7.0512
H	−0.1969	2.9107	−5.7450
H	0.6216	4.3657	−6.3193
H	−3.6326	5.8104	−4.1103
H	3.7115	3.7743	−2.3793
H	5.7854	3.3865	−3.6989
H	6.7828	1.1097	−3.7937
H	5.7019	−0.7650	−2.5928
H	3.5913	−0.3746	−1.3343
C	−5.1057	−3.9512	2.9550
C	−3.6314	−3.6540	3.2468
O	−3.3046	−2.6583	3.8886
C	−5.8303	−2.7017	2.3997
C	−5.4869	−2.4559	0.9468
N	−6.4524	−2.6485	0.0398
O	−4.3313	−2.1126	0.5930
N	−2.7132	−4.5777	2.8500
C	−1.3130	−4.4013	3.2131
C	−0.6279	−3.3230	2.3609
O	−1.0686	−3.0028	1.2497
C	−0.7067	−5.7826	2.8962
C	−1.4940	−6.2131	1.6494
C	−2.9159	−5.6857	1.9070
N	0.5231	−2.8675	2.8878
C	1.3978	−1.9662	2.1816
C	2.8631	−2.3370	2.3480
O	3.2701	−3.4944	2.2856
N	3.6704	−1.2517	2.4657
C	5.1106	−1.3302	2.5500
C	5.6770	−0.9409	3.9391
O	6.8492	−0.5713	4.0425
C	5.7563	−0.5397	1.4095
O	5.2921	0.8092	1.3427
N	4.8289	−1.1033	4.9787
C	5.2493	−0.9543	6.3702
H	−6.9112	−2.8260	2.5034
H	−5.5160	−1.8172	2.9615
H	−5.2473	−4.8071	2.2916
H	−0.8980	−6.4626	3.7332
H	0.3714	−5.7295	2.7325
H	−1.4792	−7.2924	1.4788

H	−1.0722	−5.7226	0.7706
H	−3.5516	−6.4623	2.3522
H	−3.3949	−5.3390	0.9861
H	−1.2341	−4.1317	4.2694
H	1.2244	−0.9344	2.4937
H	1.1732	−2.0477	1.1200
H	6.8314	−0.4986	1.5865
H	5.5740	−1.0548	0.4602
H	4.4432	0.8568	0.8600
H	5.3600	−2.3894	2.4148
H	6.1030	−0.2760	6.4040
H	1.9189	8.1109	−1.4522
H	−4.2794	4.2474	−3.5871
H	−2.5702	4.2231	−1.8243
H	−0.2635	7.8684	−0.9562
H	5.5512	−1.9179	6.8000
H	4.4131	−0.5482	6.9415
H	3.8823	−1.4534	4.8132
H	0.8012	−3.0346	3.8552
H	−7.3485	−3.0698	0.2793
H	−6.1652	−2.6423	−0.9315
H	3.2422	−0.3474	2.6019
H	−5.5573	−4.2078	3.9193
C	−10.5227	−5.7105	0.9636
C	−9.7339	−4.7088	0.1482
O	−8.7901	−4.0955	0.6522
C	−11.2384	−4.9476	2.0823
N	−10.1817	−4.4662	−1.1044
C	−9.6089	−3.4270	−1.9395
C	−9.1751	6.1160	3.9387
C	−8.8262	5.0154	2.9450
C	−8.4780	5.5265	1.5467
N	−7.9712	4.5658	0.7295
O	−8.6712	6.6878	1.1959
H	−10.5089	−4.3953	2.6784
H	−11.7827	−5.6310	2.7401
H	−11.9542	−4.2296	1.6702
H	−11.2361	−6.2662	0.3449
H	−8.6819	−3.7565	−2.4223
H	−7.9933	4.4063	3.3083
H	−9.6708	4.3244	2.8360
H	−7.7604	3.6217	1.0616
H	−7.7125	4.8356	−0.2071
H	−9.9903	6.7354	3.5568
H	−9.4750	5.6874	4.9000
H	−8.3210	6.7781	4.1086
H	−9.8151	−6.4287	1.3883
H	−9.3826	−2.5503	−1.3287
H	−10.3272	−3.1470	−2.7117
H	−10.9458	−5.0175	−1.4609

TS3b

C	11.4215	5.2116	−0.1968
C	10.9133	4.0378	−1.0563
C	9.5708	3.4964	−0.6530
C	8.3415	3.7545	−1.2160
N	9.4233	2.6021	0.3958
C	8.1321	2.3303	0.4610
N	7.4307	3.0090	−0.4946
H	10.7391	6.0654	−0.2610
H	11.6391	3.2180	−1.0047
H	10.8673	4.3523	−2.1061
H	8.0349	4.3732	−2.0425
H	7.6637	1.6295	1.1385
H	6.4434	2.9370	−0.7033
C	−9.4601	2.5999	−4.4113
C	−8.2946	3.0677	−3.5204
C	−7.3507	1.9636	−3.0848
C	−6.5520	1.2834	−4.0168
C	−7.2134	1.6088	−1.7385
C	−5.6543	0.2961	−3.6217
C	−6.3105	0.6280	−1.3209
C	−5.5178	−0.0341	−2.2655
O	−4.6125	−1.0030	−1.9438
H	−10.0801	1.8675	−3.8840
H	−8.6984	3.5606	−2.6284
H	−7.7278	3.8366	−4.0632
H	−6.6184	1.5422	−5.0712
H	−7.7906	2.1101	−0.9674
H	−5.0285	−0.2140	−4.3467

H	−6.2143	0.4243	−0.2596
H	−4.4912	−1.0833	−0.9627
C	−0.4239	−6.3635	−5.3127
C	−0.5492	−6.0471	−3.8149
C	−1.9309	−5.6392	−3.3385
C	−2.5106	−6.2609	−2.2284
C	−2.6539	−4.5953	−3.9399
C	−3.7529	−5.8645	−1.7318
C	−3.8833	−4.1682	−3.4442
C	−4.4391	−4.8095	−2.3345
O	−5.6677	−4.3860	−1.8931
H	−0.6823	−5.4961	−5.9283
H	−0.2267	−6.9196	−3.2356
H	0.1619	−5.2494	−3.5671
H	−1.9817	−7.0727	−1.7366
H	−2.2457	−4.0919	−4.8104
H	−4.1862	−6.3752	−0.8757
H	−4.4095	−3.3275	−3.8815
H	−5.8434	−4.7692	−1.0225
C	4.8493	−4.0391	−5.5859
C	5.0698	−4.8620	−4.3371
O	6.1434	−5.3993	−4.0710
C	5.1932	−2.5583	−5.3462
H	5.4908	−4.4512	−6.3689
H	6.2442	−2.4508	−5.0666
H	4.5826	−2.1322	−4.5432
H	5.0173	−1.9693	−6.2508
N	3.9940	−4.9227	−3.5077
C	4.1332	−5.4721	−2.1737
C	2.7832	−5.6327	−1.4829
C	1.9612	−4.3395	−1.3483
C	0.7264	−4.5947	−0.4847
C	2.7779	−3.1746	−0.7912
H	3.2141	−4.3083	−3.6896
H	4.7898	−4.8346	−1.5698
H	2.9740	−6.0308	−0.4779
H	2.1852	−6.3866	−2.0113
H	1.6025	−4.0414	−2.3442
H	1.0308	−4.8604	0.5346
H	0.0951	−3.7075	−0.4146
H	0.1209	−5.4148	−0.8824
H	3.6063	−2.8880	−1.4417
H	3.1959	−3.4182	0.1926
H	2.1385	−2.2972	−0.6782
C	9.0541	−5.4314	−0.8592
C	7.7347	−4.6481	−0.8598
C	7.8871	−3.1655	−1.1356
C	7.2283	−2.5821	−2.2245
C	8.6577	−2.3373	−0.3069
C	7.3211	−1.2126	−2.4717
C	8.7558	−0.9661	−0.5505
C	8.0826	−0.3971	−1.6356
H	9.7484	−5.0530	−0.1019
H	7.2399	−4.7836	0.1127
H	7.0744	−5.0789	−1.6173
H	6.6502	−3.2196	−2.8828
H	9.1756	−2.7655	0.5475
H	6.7854	−0.7784	−3.3095
H	9.3473	−0.3319	0.1024
H	8.1514	0.6695	−1.8211
C	−2.3496	0.7083	0.6082
C	−1.2214	1.1191	−0.0463
C	−1.2855	2.4171	−0.6701
N	−0.2149	2.9908	−1.1736
N	−2.4723	3.0948	−0.7455
C	−3.5181	2.6404	−0.0853
N	−3.4803	1.4856	0.6159
S	2.1047	−0.4172	−3.4484
C	1.7936	0.4480	−1.9309
C	−4.7633	3.4663	−0.0598
N	0.5870	−0.0300	−1.3877
C	−0.0330	−1.0480	−2.1388
C	−1.3430	−1.6249	−1.6986
C	0.6383	−1.3820	−3.2634
C	0.2869	−2.3555	−4.3367
C	0.0617	0.3101	−0.0424
H	0.7685	2.6680	−0.9168
H	−0.2742	3.8874	−1.6522
H	−0.1050	−0.6124	0.5157
H	0.8473	0.8638	0.4546
H	−5.6486	2.8840	0.1873
H	−4.6551	4.2547	0.6936
H	−4.8889	3.9525	−1.0288
H	−2.4237	−0.2382	1.1313
H	−1.3503	−1.8954	−0.6408
H	−1.5661	−2.5260	−2.2703
H	−2.1661	−0.9268	−1.8715
H	−0.5596	−2.9698	−4.0331
H	1.1249	−3.0284	−4.5648
C	1.6007	−3.0135	7.3621
C	2.2365	−1.7092	7.8427
C	1.9621	−0.5193	6.9407
O	1.9784	−0.8347	5.6447
O	1.7772	0.6084	7.3676
C	−8.4096	2.8175	4.1014
C	−6.9293	2.5721	3.7647
C	−6.6506	2.0043	2.3541
O	−7.3985	2.3842	1.4122
O	−5.6385	1.2457	2.2383
H	−8.5287	3.5335	4.9205
H	−8.9327	3.1896	3.2214
H	−6.4475	1.9265	4.5041
H	−6.3831	3.5263	3.7976
C	−0.9462	1.8681	5.5714
C	−0.8863	1.2878	4.1550
C	0.4104	1.5937	3.4799
C	0.6808	2.3867	2.3949
N	1.6054	1.0374	3.9164
C	2.5521	1.4629	3.0891
N	2.0418	2.3006	2.1608
H	−0.1107	1.5127	6.1798
H	−1.0270	0.1999	4.1940
H	−1.7076	1.6806	3.5451
H	0.0389	2.9805	1.7657
H	3.5881	1.1642	3.1190
H	2.4279	2.4483	1.2079
C	5.4438	6.9930	3.3468
C	5.5119	5.5311	3.7979
C	5.7247	4.4988	2.6743
C	5.8007	3.0917	3.2835
C	4.6262	4.5791	1.6054
H	4.5795	7.1777	2.7013
H	4.5851	5.2745	4.3303
H	6.3216	5.4192	4.5310
H	6.6872	4.7125	2.1869
H	4.8963	2.8794	3.8644
H	5.8950	2.3150	2.5197
H	6.6574	2.9933	3.9582
H	3.6356	4.4668	2.0583
H	4.7325	3.7869	0.8564
H	4.6467	5.5330	1.0690
H	0.0182	−1.8500	−5.2731
H	−8.8972	1.8826	4.4004
H	11.4853	4.9134	0.8539
H	12.4141	5.5382	−0.5249
H	−10.0961	3.4424	−4.7026
H	−9.0902	2.1231	−5.3249
H	−0.8918	2.9617	5.5396
H	−1.8815	1.5880	6.0666
H	6.3425	7.2768	2.7865
H	5.3616	7.6656	4.2072
H	1.9013	−1.4316	8.8452
H	3.3268	−1.8280	7.8928
H	0.5141	−2.9116	7.2748
H	1.8087	−3.8261	8.0656
H	1.9951	−3.2937	6.3824
H	8.8764	−6.4918	−0.6528
H	9.5471	−5.3523	−1.8331
H	4.6382	−6.4377	−2.2623
H	0.6033	−6.6503	−5.5582
H	−1.0891	−7.1838	−5.5998
H	3.8089	−4.1306	−5.9213
H	1.7931	−0.0006	5.0387
H	−4.3332	1.2308	1.1856
C	2.2186	7.0765	−2.5173
C	0.9336	6.3658	−2.1644
O	0.7314	5.2012	−2.5055
N	0.0127	7.0804	−1.4565
C	−1.3450	6.5666	−1.3586
C	−1.9429	6.3453	−2.7607
O	−1.7282	7.1561	−3.6588
N	−2.6998	5.2343	−2.8853
C	−3.2556	4.8421	−4.1739
C	−3.2654	3.3210	−4.3509
C	−1.8919	2.6358	−4.2561
C	−2.0414	1.1194	−4.4222
C	−0.8683	3.2103	−5.2407
C	3.7323	2.0773	−2.2274
C	4.0009	3.4464	−2.0136
C	5.0314	4.0952	−2.6883
C	5.8367	3.3966	−3.5929
C	5.6039	2.0362	−3.7908
C	4.5723	1.3827	−3.1183
C	2.5932	1.5078	−1.4514
O	2.1476	2.2719	−0.4657
C	3.6937	−0.1929	−0.3036
O	3.0507	−0.1646	0.7068
O	4.6253	−0.5644	−0.9489
H	2.4731	7.8675	−1.8068
H	3.0220	6.3411	−2.5645
H	−1.3588	5.6499	−0.7643
H	−1.9500	7.3096	−0.8323
H	−3.9396	2.8712	−3.6110
H	−3.7133	3.1048	−5.3309
H	−1.4980	2.8108	−3.2533
H	0.0763	2.6614	−5.1690
H	−1.2270	3.1333	−6.2756
H	−0.6483	4.2600	−5.0277
H	−2.4184	0.8689	−5.4227
H	−2.7444	0.7060	−3.6922
H	−1.0798	0.6147	−4.2880
H	−2.6602	5.3349	−4.9458
H	3.3613	3.9778	−1.3215
H	5.2076	5.1526	−2.5067
H	6.6373	3.9005	−4.1258
H	6.2379	1.4696	−4.4670
H	4.4665	0.3164	−3.2519
C	−5.2047	−3.1551	3.3779
C	−3.6943	−3.1292	3.5573
O	−3.1556	−2.3539	4.3428
C	−5.7645	−1.9211	2.6315
C	−5.4392	−1.9316	1.1516
N	−6.3163	−2.5452	0.3306
O	−4.3880	−1.4312	0.7132
N	−2.9803	−4.0714	2.8811
C	−1.5661	−4.2339	3.1655
C	−0.7097	−3.1120	2.5592
O	−1.0847	−2.4288	1.6018
C	−1.2519	−5.5941	2.5134
C	−2.1580	−5.5889	1.2747
C	−3.4417	−4.9004	1.7621
N	0.5031	−3.0100	3.1269
C	1.5482	−2.1560	2.6229
C	2.8815	−2.6723	3.1604
O	2.9461	−3.6695	3.8843
N	3.9467	−1.9333	2.7841
C	5.3182	−2.2525	3.1290
C	5.8436	−1.5191	4.3782
O	7.0348	−1.5787	4.6709
C	6.2299	−1.9671	1.9378
O	6.0725	−0.6001	1.5583
N	4.9249	−0.8407	5.1035
C	5.3009	−0.2117	6.3517
H	−6.8514	−1.9002	2.7574
H	−5.3576	−0.9969	3.0454
H	−5.5521	−4.0726	2.8953
H	−1.5393	−6.3971	3.2007
H	−0.1931	−5.7125	2.2764
H	−2.3467	−6.5891	0.8786
H	−1.7096	−4.9907	0.4826
H	−4.1844	−5.6354	2.1021
H	−3.8922	−4.2860	0.9789
H	−1.4115	−4.2356	4.2482
H	1.4066	−1.1179	2.9349
H	1.5546	−2.1648	1.5312
H	7.2611	−2.1753	2.2371
H	5.9561	−2.6275	1.1075
H	6.1872	−0.5551	0.5981
H	5.3657	−3.3208	3.3645
H	6.2185	0.3624	6.2060
H	2.1075	7.5220	−3.5117
H	−4.2809	5.2248	−4.2728
H	−2.7029	4.5518	−2.1251

H	0.1198	8.0838	−1.4563
H	5.4948	−0.9482	7.1425
H	4.5008	0.4563	6.6729
H	3.9385	−0.9836	4.9309
H	0.8001	−3.6388	3.8647
H	−7.2211	−2.8961	0.6339
H	−6.1072	−2.5477	−0.6611
H	3.8028	−1.1590	2.1405
H	−5.6129	−3.1405	4.3925
C	−10.8723	−4.9416	1.5725
C	−9.9393	−4.1929	0.6348
O	−8.7656	−3.9673	0.9423
C	−11.3810	−4.0100	2.6824
N	−10.4917	−3.7635	−0.5246
C	−9.7621	−2.9573	−1.4906
C	−8.9035	7.1011	3.3984
C	−8.6019	5.8504	2.5835
C	−8.2121	6.1359	1.1331
N	−7.7910	5.0386	0.4521
O	−8.2898	7.2567	0.6350
H	−10.5376	−3.5768	3.2261
H	−12.0068	−4.5570	3.3927
H	−11.9735	−3.1885	2.2678
H	−11.7136	−5.3782	1.0219
H	−8.8038	−3.4283	−1.7266
H	−7.8018	5.2667	3.0483
H	−9.4759	5.1890	2.5655
H	−7.7114	4.1064	0.8730
H	−7.5319	5.1655	−0.5142
H	−9.6920	7.6906	2.9241
H	−9.2210	6.8349	4.4114
H	−8.0219	7.7444	3.4703
H	−10.2972	−5.7633	2.0077
H	−9.5588	−1.9514	−1.1082
H	−10.3510	−2.8783	−2.4050
H	−11.4685	−3.9490	−0.6877

Int3b

C	11.4215	5.2116	−0.1968
C	10.9133	4.0378	−1.0563
C	9.5708	3.4964	−0.6530
C	8.3415	3.7545	−1.2160
N	9.4233	2.6021	0.3958
C	8.1321	2.3303	0.4610
N	7.4307	3.0090	−0.4946
H	10.7391	6.0654	−0.2610
H	11.6391	3.2180	−1.0047
H	10.8673	4.3523	−2.1061
H	8.0349	4.3732	−2.0425
H	7.6637	1.6295	1.1385
H	6.4434	2.9370	−0.7033
C	−9.4601	2.5999	−4.4113
C	−8.2946	3.0677	−3.5204
C	−7.3507	1.9636	−3.0848
C	−6.5520	1.2834	−4.0168
C	−7.2134	1.6088	−1.7385
C	−5.6543	0.2961	−3.6217
C	−6.3105	0.6280	−1.3209
C	−5.5178	−0.0341	−2.2655
O	−4.6125	−1.0030	−1.9438
H	−10.0801	1.8675	−3.8840
H	−8.6984	3.5606	−2.6284
H	−7.7278	3.8366	−4.0632
H	−6.6184	1.5422	−5.0712
H	−7.7906	2.1101	−0.9674
H	−5.0285	−0.2140	−4.3467
H	−6.2143	0.4243	−0.2596
H	−4.4912	−1.0833	−0.9627
C	−0.4239	−6.3635	−5.3127
C	−0.5492	−6.0471	−3.8149
C	−1.9309	−5.6392	−3.3385
C	−2.5106	−6.2609	−2.2284
C	−2.6539	−4.5953	−3.9399
C	−3.7529	−5.8645	−1.7318
C	−3.8833	−4.1682	−3.4442
C	−4.4391	−4.8095	−2.3345
O	−5.6677	−4.3860	−1.8931
H	−0.6823	−5.4961	−5.9283
H	−0.2267	−6.9196	−3.2356
H	0.1619	−5.2494	−3.5671
H	−1.9817	−7.0727	−1.7366
H	−2.2457	−4.0919	−4.8104
H	−4.1862	−6.3752	−0.8757
H	−4.4095	−3.3275	−3.8815
H	−5.8434	−4.7692	−1.0225
C	4.8493	−4.0391	−5.5859
C	5.0698	−4.8620	−4.3371
O	6.1434	−5.3993	−4.0710
C	5.1932	−2.5583	−5.3462
H	5.4908	−4.4512	−6.3689
H	6.2442	−2.4508	−5.0666
H	4.5826	−2.1322	−4.5432
H	5.0173	−1.9693	−6.2508
N	3.9940	−4.9227	−3.5077
C	4.1332	−5.4721	−2.1737
C	2.7832	−5.6327	−1.4829
C	1.9612	−4.3395	−1.3483
C	0.7264	−4.5947	−0.4847
C	2.7779	−3.1746	−0.7912
H	3.2141	−4.3083	−3.6896
H	4.7898	−4.8346	−1.5698
H	2.9740	−6.0308	−0.4779
H	2.1852	−6.3866	−2.0113
H	1.6025	−4.0414	−2.3442
H	1.0308	−4.8604	0.5346
H	0.0951	−3.7075	−0.4146
H	0.1209	−5.4148	−0.8824
H	3.6063	−2.8880	−1.4417
H	3.1959	−3.4182	0.1926
H	2.1385	−2.2972	−0.6782
C	9.0541	−5.4314	−0.8592
C	7.7347	−4.6481	−0.8598
C	7.8871	−3.1655	−1.1356
C	7.2283	−2.5821	−2.2245
C	8.6577	−2.3373	−0.3069
C	7.3211	−1.2126	−2.4717
C	8.7558	−0.9661	−0.5505
C	8.0826	−0.3971	−1.6356
H	9.7484	−5.0530	−0.1019
H	7.2399	−4.7836	0.1127
H	7.0744	−5.0789	−1.6173
H	6.6502	−3.2196	−2.8828
H	9.1756	−2.7655	0.5475
H	6.7854	−0.7784	−3.3095
H	9.3473	−0.3319	0.1024
H	8.1514	0.6695	−1.8211
C	−2.3496	0.7083	0.6082
C	−1.2214	1.1191	−0.0463
C	−1.2855	2.4171	−0.6701
N	−0.2149	2.9908	−1.1736
N	−2.4723	3.0948	−0.7455
C	−3.5181	2.6404	−0.0853
N	−3.4803	1.4856	0.6159
S	2.1047	−0.4172	−3.4484
C	1.7936	0.4480	−1.9309
C	−4.7633	3.4663	−0.0598
N	0.5870	−0.0300	−1.3877
C	−0.0330	−1.0480	−2.1388
C	−1.3430	−1.6249	−1.6986
C	0.6383	−1.3820	−3.2634
C	0.2869	−2.3555	−4.3367
C	0.0617	0.3101	−0.0424
H	0.7685	2.6680	−0.9168
H	−0.2742	3.8874	−1.6522
H	−0.1050	−0.6124	0.5157
H	0.8473	0.8638	0.4546
H	−5.6486	2.8840	0.1873
H	−4.6551	4.2547	0.6936
H	−4.8889	3.9525	−1.0288
H	−2.4237	−0.2382	1.1313
H	−1.3503	−1.8954	−0.6408
H	−1.5661	−2.5260	−2.2703
H	−2.1661	−0.9268	−1.8715
H	−0.5596	−2.9698	−4.0331
H	1.1249	−3.0284	−4.5648
C	1.6007	−3.0135	7.3621
C	2.2365	−1.7092	7.8427
C	1.9621	−0.5193	6.9407
O	1.9784	−0.8347	5.6447
O	1.7772	0.6084	7.3676
C	−8.4096	2.8175	4.1014
C	−6.9293	2.5721	3.7647
C	−6.6506	2.0043	2.3541
O	−7.3985	2.3842	1.4122
O	−5.6385	1.2457	2.2383
H	−8.5287	3.5335	4.9205
H	−8.9327	3.1896	3.2214
H	−6.4475	1.9265	4.5041
H	−6.3831	3.5263	3.7976
C	−0.9462	1.8681	5.5714
C	−0.8863	1.2878	4.1550
C	0.4104	1.5937	3.4799
C	0.6808	2.3867	2.3949
N	1.6054	1.0374	3.9164
C	2.5521	1.4629	3.0891
N	2.0418	2.3006	2.1608
H	−0.1107	1.5127	6.1798
H	−1.0270	0.1999	4.1940
H	−1.7076	1.6806	3.5451
H	0.0389	2.9805	1.7657
H	3.5881	1.1642	3.1190
H	2.4279	2.4483	1.2079
C	5.4438	6.9930	3.3468
C	5.5119	5.5311	3.7979
C	5.7247	4.4988	2.6743
C	5.8007	3.0917	3.2835
C	4.6262	4.5791	1.6054
H	4.5795	7.1777	2.7013
H	4.5851	5.2745	4.3303
H	6.3216	5.4192	4.5310
H	6.6872	4.7125	2.1869
H	4.8963	2.8794	3.8644
H	5.8950	2.3150	2.5197
H	6.6574	2.9933	3.9582
H	3.6356	4.4668	2.0583
H	4.7325	3.7869	0.8564
H	4.6467	5.5330	1.0690
H	0.0182	−1.8500	−5.2731
H	−8.8972	1.8826	4.4004
H	11.4853	4.9134	0.8539
H	12.4141	5.5382	−0.5249
H	−10.0961	3.4424	−4.7026
H	−9.0902	2.1231	−5.3249
H	−0.8918	2.9617	5.5396
H	−1.8815	1.5880	6.0666
H	6.3425	7.2768	2.7865
H	5.3616	7.6656	4.2072
H	1.9013	−1.4316	8.8452
H	3.3268	−1.8280	7.8928
H	0.5141	−2.9116	7.2748
H	1.8087	−3.8261	8.0656
H	1.9951	−3.2937	6.3824
H	8.8764	−6.4918	−0.6528
H	9.5471	−5.3523	−1.8331
H	4.6382	−6.4377	−2.2623
H	0.6033	−6.6503	−5.5582
H	−1.0891	−7.1838	−5.5998
H	3.8089	−4.1306	−5.9213
H	1.7931	−0.0006	5.0387
H	−4.3332	1.2308	1.1856
C	2.2186	7.0765	−2.5173
C	0.9336	6.3658	−2.1644
O	0.7314	5.2012	−2.5055
N	0.0127	7.0804	−1.4565
C	−1.3450	6.5666	−1.3586
C	−1.9429	6.3453	−2.7607
O	−1.7282	7.1561	−3.6588
N	−2.6998	5.2343	−2.8853
C	−3.2556	4.8421	−4.1739
C	−3.2654	3.3210	−4.3509
C	−1.8919	2.6358	−4.2561
C	−2.0414	1.1194	−4.4222
C	−0.8683	3.2103	−5.2407
C	3.7323	2.0773	−2.2274
C	4.0009	3.4464	−2.0136
C	5.0314	4.0952	−2.6883
C	5.8367	3.3966	−3.5929
C	5.6039	2.0362	−3.7908
C	4.5723	1.3827	−3.1183
C	2.5932	1.5078	−1.4514
O	2.1476	2.2719	−0.4657
C	3.6937	−0.1929	−0.3036

O	3.0507	−0.1646	0.7068
O	4.6253	−0.5644	−0.9489
H	2.4731	7.8675	−1.8068
H	3.0220	6.3411	−2.5645
H	−1.3588	5.6499	−0.7643
H	−1.9500	7.3096	−0.8323
H	−3.9396	2.8712	−3.6110
H	−3.7133	3.1048	−5.3309
H	−1.4980	2.8108	−3.2533
H	0.0763	2.6614	−5.1690
H	−1.2270	3.1333	−6.2756
H	−0.6483	4.2600	−5.0277
H	−2.4184	0.8689	−5.4227
H	−2.7444	0.7060	−3.6922
H	−1.0798	0.6147	−4.2880
H	−2.6602	5.3349	−4.9458
H	3.3613	3.9778	−1.3215
H	5.2076	5.1526	−2.5067
H	6.6373	3.9005	−4.1258
H	6.2379	1.4696	−4.4670
H	4.4665	0.3164	−3.2519
C	−5.2047	−3.1551	3.3779
C	−3.6943	−3.1292	3.5573
O	−3.1556	−2.3539	4.3428
C	−5.7645	−1.9211	2.6315
C	−5.4392	−1.9316	1.1516
N	−6.3163	−2.5452	0.3306
O	−4.3880	−1.4312	0.7132
N	−2.9803	−4.0714	2.8811
C	−1.5661	−4.2339	3.1655
C	−0.7097	−3.1120	2.5592
O	−1.0847	−2.4288	1.6018
C	−1.2519	−5.5941	2.5134
C	−2.1580	−5.5889	1.2747
C	−3.4417	−4.9004	1.7621
N	0.5031	−3.0100	3.1269
C	1.5482	−2.1560	2.6229
C	2.8815	−2.6723	3.1604
O	2.9461	−3.6695	3.8843
N	3.9467	−1.9333	2.7841
C	5.3182	−2.2525	3.1290
C	5.8436	−1.5191	4.3782
O	7.0348	−1.5787	4.6709
C	6.2299	−1.9671	1.9378
O	6.0725	−0.6001	1.5583
N	4.9249	−0.8407	5.1035
C	5.3009	−0.2117	6.3517
H	−6.8514	−1.9002	2.7574
H	−5.3576	−0.9969	3.0454
H	−5.5521	−4.0726	2.8953
H	−1.5393	−6.3971	3.2007
H	−0.1931	−5.7125	2.2764
H	−2.3467	−6.5891	0.8786
H	−1.7096	−4.9907	0.4826
H	−4.1844	−5.6354	2.1021
H	−3.8922	−4.2860	0.9789
H	−1.4115	−4.2356	4.2482
H	1.4066	−1.1179	2.9349
H	1.5546	−2.1648	1.5312
H	7.2611	−2.1753	2.2371
H	5.9561	−2.6275	1.1075
H	6.1872	−0.5551	0.5981
H	5.3657	−3.3208	3.3645
H	6.2185	0.3624	6.2060
H	2.1075	7.5220	−3.5117
H	−4.2809	5.2248	−4.2728
H	−2.7029	4.5518	−2.1251
H	0.1198	8.0838	−1.4563
H	5.4948	−0.9482	7.1425
H	4.5008	0.4563	6.6729
H	3.9385	−0.9836	4.9309
H	0.8001	−3.6388	3.8647
H	−7.2211	−2.8961	0.6339
H	−6.1072	−2.5477	−0.6611
H	3.8028	−1.1590	2.1405
H	−5.6129	−3.1405	4.3925
C	−10.8723	−4.9416	1.5725
C	−9.9393	−4.1929	0.6348
O	−8.7656	−3.9673	0.9423
C	−11.3810	−4.0100	2.6824
N	−10.4917	−3.7635	−0.5246
C	−9.7621	−2.9573	−1.4906
C	−8.9035	7.1011	3.3984
C	−8.6019	5.8504	2.5835
C	−8.2121	6.1359	1.1331
N	−7.7910	5.0386	0.4521
O	−8.2898	7.2567	0.6350
H	−10.5376	−3.5768	3.2261
H	−12.0068	−4.5570	3.3927
H	−11.9735	−3.1885	2.2678
H	−11.7136	−5.3782	1.0219
H	−8.8038	−3.4283	−1.7266
H	−7.8018	5.2667	3.0483
H	−9.4759	5.1890	2.5655
H	−7.7114	4.1064	0.8730
H	−7.5319	5.1655	−0.5142
H	−9.6920	7.6906	2.9241
H	−9.2210	6.8349	4.4114
H	−8.0219	7.7444	3.4703
H	−10.2972	−5.7633	2.0077
H	−9.5588	−1.9514	−1.1082
H	−10.3510	−2.8783	−2.4050
H	−11.4685	−3.9490	−0.6877
